# Gene expression markers of Tumor Infiltrating Leukocytes

**DOI:** 10.1186/s40425-017-0215-8

**Published:** 2017-02-21

**Authors:** Patrick Danaher, Sarah Warren, Lucas Dennis, Leonard D’Amico, Andrew White, Mary L. Disis, Melissa A. Geller, Kunle Odunsi, Joseph Beechem, Steven P. Fling

**Affiliations:** 1 NanoString Technologies, Inc., 530 Fairview Ave N, Seattle, WA 98109 USA; 2 Tumor Vaccine Group, University of Washington, 850 Republican Street, Box 358050, Seattle, WA 98109 USA; 3 Department of Medicine, Division of Oncology, University of Washington, 825 Eastlake Ave E., Seattle, WA 98109-1023 USA; 4 Department of Obstetrics, Gynecology and Women’s Health, University of Minnesota, Mayo Building, 420 Delaware St SE, Minneapolis, MN 55455 USA; 5 Department of Gynecologic Oncology and Center for Immunotherapy, Roswell Park Cancer Institute, Elm and Carlton Streets, Buffalo, NY 14263 USA

**Keywords:** Gene expression, Tumor infiltrating lymphocytes, Immunotherapies, TILs, Cell types

## Abstract

**Background:**

Assays of the abundance of immune cell populations in the tumor microenvironment promise to inform immune oncology research and the choice of immunotherapy for individual patients. We propose to measure the intratumoral abundance of various immune cell populations with gene expression. In contrast to IHC and flow cytometry, gene expression assays yield high information content from a clinically practical workflow. Previous studies of gene expression in purified immune cells have reported hundreds of genes showing enrichment in a single cell type, but the utility of these genes in tumor samples is unknown. We use co-expression patterns in large tumor gene expression datasets to evaluate previously reported candidate cell type marker genes lists, eliminate numerous false positives and identify a subset of high confidence marker genes.

**Methods:**

Using a novel statistical tool, we use co-expression patterns in 9986 samples from The Cancer Genome Atlas (TCGA) to evaluate previously reported cell type marker genes. We compare immune cell scores derived from these genes to measurements from flow cytometry and immunohistochemistry. We characterize the reproducibility of our cell scores in replicate runs of RNA extracted from FFPE tumor tissue.

**Results:**

We identify a list of 60 marker genes whose expression levels measure 14 immune cell populations. Cell type scores calculated from these genes are concordant with flow cytometry and IHC readings, show high reproducibility in replicate RNA samples from FFPE tissue and enable detailed analyses of the anti-tumor immune response in TCGA. In an immunotherapy dataset, they separate responders and non-responders early on therapy and provide an intricate picture of the effects of checkpoint inhibition. Most genes previously reported to be enriched in a single cell type have co-expression patterns inconsistent with cell type specificity.

**Conclusions:**

Due to their concise gene set, computational simplicity and utility in tumor samples, these cell type gene signatures may be useful in future discovery research and clinical trials to understand how tumors and therapeutic intervention shape the immune response.

**Electronic supplementary material:**

The online version of this article (doi:10.1186/s40425-017-0215-8) contains supplementary material, which is available to authorized users.

## Background

The abundance and composition of the immune cells infiltrating a tumor predict both a patient’s prognosis [1–5] and the optimal immunotherapy for their disease [6]. Therefore techniques to profile tumor infiltrating lymphocytes (TILs) in a clinical setting are needed. While flow cytometry is the current gold standard for quantifying immune cell populations, sample and/or workflow constraints make it infeasible in many research and clinical applications, especially those utilizing FFPE tissue. Immunohistochemistry (IHC) has been shown to be clinically useful [2], but it cannot assay more than a few immune markers without using up excessive tissue. In contrast to these older technologies, gene expression profiling promises a clinically practical way to measure the full diversity of the tumor immune infiltrate in settings and sample types where flow cytometry is unworkable, additionally allowing the simultaneous measurement of hundreds to thousands of other relevant genes. Therefore, we propose to identify genes whose expression levels can be used to measure the abundance of various immune cell populations within the tumor microenvironment.

Previous authors have identified genes specific to purified immune cell populations [7–9] and used these genes to quantify immune populations in tumors [9, 10]. However, these genes were discovered using purified cells and not immune cells taken from the tumor microenvironment, and so any differences between intratumoral and in vitro gene expression patterns will compromise their utility in tumor samples. To address this concern, we propose a novel computational method for testing whether previously reported cell type marker genes are useful in tumor data. We then apply this method to data from The Cancer Genome Atlas (TCGA) to derive a set of 60 marker genes for 14 immune cell populations.

Our final gene list exhibits sufficiently strong cell type specificity to allow measurement of immune cell populations with scores computed as the simple average log expression of their marker genes. In data from ovarian cancer patients, these cell type scores are concordant with both immunohistochemistry (IHC) and flow cytometry. In replicate RNA samples, they display substantially better reproducibility than typical IHC readings. In TCGA data, they enable detailed analyses of anti-tumoral immunity. In an immunotherapy dataset, they provide early discrimination between responders and non-responders and reveal an intricate picture of the immune response to checkpoint inhibition. Due to their concise gene set, computational simplicity and utility in tumor samples, these cell type gene signatures may be useful in future discovery research and clinical trials to understand how tumors and therapeutic intervention shape the immune response.

## Methods

### Derivation of candidate cell type marker genes from the literature

The first step in our marker gene identification process is to identify previously reported cell type markers. Fortunately, the literature is rich in papers measuring gene expression in isolated immune cell populations. A number of authors, most notably [7] and [9], have used meta-analyses of these experiments to discover genes that are predominantly expressed within a single immune cell population. Our list of candidate marker genes is primarily drawn from [7], whose cell type-specific gene lists we took verbatim as the foundation of our candidate markers list. To derive candidate markers for cell types missing in [7], we took genes that [9] reported to be highly enriched in one cell type vs. the maximum seen in all other cell types. We also included well-known markers for exhausted CD8 cells [11–13] and FOXP3 for Tregs. Our full list of candidate marker genes can be found in Additional file 1: Table S3.

### Approach to evaluation of candidate cell type marker genes in tumor gene expression data

The literature on cell type specific gene expression is a powerful source of candidate marker genes, but there are a variety of mechanisms by which poor marker genes may have entered the literature. First, early microarray studies were frequently underpowered, noisy and rife with batch effects and therefore could indicate spurious marker genes [14]. Second, and very significantly, the expression profile of in vitro purified cells may differ substantially from these cells’ gene expression in the tumor microenvironment [15]. Finally, genes that appear specific to one cell type in a microarray experiment may be expressed in cell types omitted from the experiment.

**Fig. 1:**
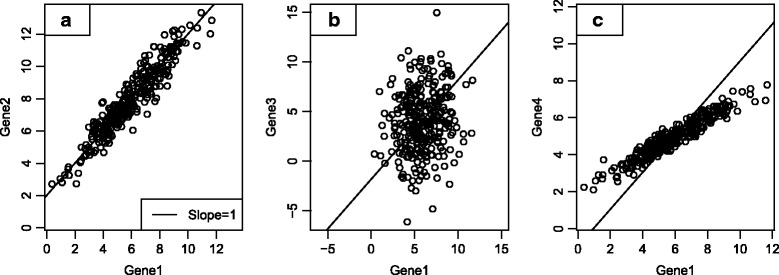
Simulated example of process for evaluating candidate genes for marker-like co-expression in a tumor dataset. **a** Example of marker-like co-expression. Genes 1 and 2 are highly correlated with a slope of 1, a pattern consistent with both genes rising and falling at the same rate. **b** Example of two genes which cannot both be markers. Gene 3 exhibits no co-expression with gene 1, showing that Genes 1 and 3 are not both good markers for the same cell type. **c** Example of two genes which cannot both be markers. Gene 4 is highly correlated with Gene 1, but with a slope different than 1, meaning they are not both markers for the same cell type

Therefore, our literature-derived candidate marker genes require validation in actual tumor expression data. Ideally, we would test whether each gene displays two properties: expression specific to a single cell type, and stable expression within that cell type. Unfortunately, we cannot directly measure a gene’s adherence to these properties in bulk tumor expression data; instead, we look for genes whose expression patterns are consistent with these properties. If two genes are both ideal markers, expressed with perfect specificity to and stability within a cell type, their expression levels will be perfectly correlated, and the ratio between them will be constant across samples. Figure 1 demonstrates this principle. Of the 4 candidate marker genes for a cell population, Genes 1 and 2 rise and fall at the same rate, a co-expression pattern consistent with both genes being driven by abundance of a single cell population. By contrast Gene 3 exhibits no such co-expression with Gene 1 and likely does not serve as a marker gene in the tumor microenvironment. Gene 4 is highly correlated with Genes 1 and 2, but its slope is different than 1. Thus while Genes 1, 2 and 4 may be regulated by the same biological process, that they do not increase at the same rate means they are not all expressed at consistent levels within a single cell type. We quantify genes’ adherence to the marker-like co-expression pattern we seek with a pairwise similarity statistic, defined in the Methods.

For each set of candidate marker genes for a single cell type, we sought a subset of genes that exhibited strong marker-like co-expression patterns in tumor gene expression data. Candidate marker genes with expression patterns like Genes 1 and 2 were selected for our final marker gene list. We discarded candidate markers like Gene 3 that were uncorrelated with other candidate markers, and we discarded candidates like Gene 4 that had non-unit slopes with other candidate markers.

Alone, co-expression patterns are insufficient to establish a group of genes as markers for a single cell type. However, when a set of genes has been previously reported to have cell type-specific expression and also displays marker-like co-expression patterns in tumor data, the cumulative evidence supports their use as cell type markers.

### Pairwise similarity statistic for quantifying marker-like co-expression patterns

If two genes are ideal cell type markers, their log expression values will be perfectly correlated with a slope of 1. The below adaptation of Pearson’s correlation metric measures a pair of genes’ adherence to this pattern:

$$ similarity\left( x, y\right) = \frac{{\displaystyle \sum}\left( x-\overline{x}\right)\left( y-\overline{y}\right)}{\frac{\left( n-1\right)}{2}\left( var(x)+ var(y)\right)}, $$

where x and y are the vectors of log-transformed, normalized expression values of the two genes, $\overline{\mathrm{x}}$ and $\overline{\mathrm{y}}$ are their sample means, and var (x) and var (y) are their sample variances. This function equals 1 when the two genes are perfectly correlated with a slope of 1 and decreases for gene pairs with low correlation or with slope diverging from 1. Since many biologically related genes will exhibit correlation unrelated to a shared cell type, mere correlation is a weak indicator of cell type markers. Similarly, gene pairs that exhibit pairwise differences with low variance are consistent with the hypothesis that they serve as cell type markers, but unless they retain this stable pairwise difference over a range of expression values and thereby achieve high correlation, they provide minimal evidence for their utility as cell type markers.

The Additional file 2: Methods and Results contain further characterization of the pairwise similarity statistic, including a short proof of its relevance (S2.5.), a simulation demonstrating its improved utility over simple Pearson correlation (S2.6.), and several examples of its use in our marker gene selection (S2.7.). Co-expression analyses have long been used to define gene sets [16–19]; this method departs from this earlier work by using co-expression as a test of a priori-derived candidate gene lists.

### Procedure for selecting marker genes with the aid of the pairwise similarity statistic

Our procedure for deriving a full list of marker genes for a cell type was as follows. First, we computed the pairwise similarity statistic between all pairs of the cell type’s candidate marker genes within each of 24 TCGA RNASeq datasets. Second, we defined a similarity matrix in which the similarity between each gene pair was calculated as the pair’s average pairwise similarity statistic across all datasets. For most cell types, hierarchical clustering of this similarity matrix identified an obvious subset of genes sharing marker-like co-expression, and we took these obvious subsets as our final marker genes. In the more challenging cell types we accepted genes with moderate (>0.4) pairwise similarity, and when better genes were available we took only genes with high (>0.6) pairwise similarity statistics. For some cell types, we allowed domain knowledge to overrule the results of this analysis and discard markers with promising co-expression pattern; details of these instances are in the Results. Additional file 3: Figures S10–S38 show these similarity matrices along with the genes we chose.

### Using marker genes to measure cell type abundance

Once we have selected a set of marker genes for a cell type, measuring the cell type’s abundance is straightforward. Assuming each marker gene is present at a fixed but unknown number of copies per cell, the average log-transformed expression of the marker genes is equal to the log-transformed abundance of the cell type, plus an unknown constant. Thus we compute cell type scores with the simple average of their marker genes’ log-transformed expression values. Because of the unknown constant, these scores do not provide absolute quantification of cell types; e.g., we cannot say, “there are 500 CD8 cells in this sample,” or, without performing some calibration exercises, “this sample has more B-cells than T-cells.” But they do allow comparison of cell abundance across samples, e.g., “this tumor has twice as many CD8 cells as the average tumor,” or, “sample 1 has twice as many Treg cells per CD8 T-cell as the average sample,” sufficient information for most scientific and clinical applications. If our scores are calculated from log2 transformed data, each unit increase in a cell score should correspond to a doubling of that cell type’s abundance.

Absolute quantification can be achieved by measuring our marker genes’ expression in a reference sample for which the absolute number of each cell type of interest is known, either through flow cytometry of IHC measurement. Future samples’ cell scores could then be calibrated to this reference sample. For example, if the reference sample had 500 macrophages and a macrophage score of 4, a sample with a macrophage score of 5 could be inferred to have 1000 macrophages. Alternatively, cell scores can be placed on a more intuitive scale by reporting each cell score’s difference from its average in a group of null or baseline samples. We employ this method in Fig. 7c, showing cell scores in a selected patient relative to their average values in immunotherapy-naïve patients.

### Flow cytometry

Whole blood was stained within 30 h of collection with 2 12-color antibody staining panels: a PBMC subset panel and a T cell subset panel. The PBMC subset panel antibody cocktail (CD3-PE CF594, CD4-FITC, CD8-PerCP Cy5.5, CD11c-AlexaFluor 700, CD14-V450, CD16-APC H7, CD19-PE-Cy5, CD45-AmCyan, CD56-PE Cy7, CD122 APC, CD123 PE, HLADR-BV605) was used to stain 100uL whole blood in BD Trucount Tubes to determine absolute numbers of various peripheral immune cell types, including monocytes, CD4 and CD8 T cells, NK cells, NKT cells, B cells, plasmacytoid dendritic cells (pDC) and myeloid dendritic cells (mDC). After staining, cells were incubated with FACs Lysing Solution (BD) for 15 min, and stored at−80 °C until acquisition. For determining activation state, as well as naïve-memory-effector subsets of CD4 and CD8 T cells, 200 uL of whole blood was incubated with the T cell subset panel antibody cocktail (CD3-FITC, CD4-APC-Cy7, CD8-PerCP Cy5.5, CD25-APC, CD28-PE-CF594, CD45-AmCyan, CD45RA-BV650, CD127-BV421, CD197-AlexaFluor 700, CD278-PE, CD279-PE-Cy7, HLADR-BV605), and subsequently lysed with Pharm Lyse solution (BD), washed, fixed with 1% PFA, and suspended in 10% DMSO before storage at−80 °C. Samples from both panels were acquired on a BD LSR II cell analysis machine and analyzed by FlowJo Cell Analysis software.

### TCGA data

Level 3 RSEM-normalized RNASeqV2 data was downloaded from TCGA and log2-transformed prior to analysis. No further preprocessing was applied.

### NanoString data

RNA from PBMC lysates (~60,000 cells per assay) and FFPE tumor biopsy sections (150–300 ng per assay) were evaluated for gene expression using the nCounter PanCancer Immune Profiling panel, which interrogates 770 immune-related genes and associated controls.

NanoString gene expression values were normalized using the best subset of the 40 reference genes included in the panel, as determined by geNorm [20]. Reference gene normalization was performed for each sample by dividing each sample’s raw count profile by the geometric mean of its reference genes. To transform expression back to an intelligible count space, all samples were then multiplied by the geometric mean of all the samples’ reference gene geometric means. The nSolver software was used to perform all normalization.

Cell scores were calculated as the average log2 normalized expression of each cell’s marker genes.

### Statistical methods: comparison to flow cytometry and IHC

We measured concordance between platforms with Pearson correlation and Root Mean Squared Error (RMSE). RMSE between matching measurements from NanoString and either flow or IHC was calculated by mean-centering each separate set of measurements and then taking the square root of the mean squared difference between matching pairs.

### Statistical methods: reproducibility analysis

To measure the proportion of variance due to noise for each cell score, we used the R package lme4 to fit a linear mixed model predicting cell score from sample ID, treating sample ID as a random effect. As a measure of the proportion of variance due to noise, we report the estimated residual variance divided by the sum of the residual variance and the between-sample variance.

### Statistical methods: application to TCGA

Cell scores were calculated as the average log2 normalized expression of each cell’s marker genes. Total TILs score was calculated as the average of all cell scores whose correlations with PTRPC (CD45) exceeded 0.6. This composite score excluded only dendritic cells, Tregs and mast cells. Cell type enrichment scores were calculated as follows. Using all TCGA data at once, simple linear regression was used to predict each cell score from Total TILs score. Cell type enrichment scores were defined as the residuals of these regressions. These scores can be interpreted as measuring the abundance or depletion of each cell population relative to total TILs. For example, a macrophage enrichment score of 1 can be interpreted to mean that a sample has twice as many macrophages as the average sample with the same total TILs.

## Results

### Only a small proportion of previously reported cell type specific genes display marker-like co-expression patterns in the tumor microenvironment

The Cancer Genome Atlas (TCGA) provides ideal data for evaluating candidate cell type marker genes through their co-expression patterns. We evaluated our literature-derived candidate marker genes in TCGA RNASeq data from 9986 samples from 32 tumor types. Details of the TCGA download used are in Additional file 1: Table S1.

TCGA data revealed previously reported cell type marker genes to have widely varying quality, with many candidate cell type marker genes displaying co-expression patterns inconsistent with cell type specificity and stability. However, most cell types had a core subset of genes with strong marker-like co-expression (Additional file 3: Figures S10–S38). These highly concordant gene sets constitute our final selected cell type markers (Table 1).

**Table 1:** Results of TCGA data evaluation of candidate cell type markers

Cell type	# Candidate genes	# Selected markers	Mean pairwise similarity statistic in TCGA	Selected marker genes
B-cells	34	9	0.59	BLK, CD19, FCRL2, MS4A1, KIAA0125, TNFRSF17, TCL1A, SPIB, PNOC
CD45	1	1	^a^NA	PTRPC
Cytotoxic cells	18	10	0.69	PRF1, GZMA, GZMB, NKG7, GZMH, KLRK1, KLRB1, KLRD1, CTSW, GNLY
DC	7	3	0.46	CCL13, CD209, HSD11B1
Exhausted CD8	5	4	0.44	LAG3, CD244, EOMES, PTGER4
Macrophages	33	4	0.71	CD68, CD84, CD163, MS4A4A
Mast cells	31	5	0.74	TPSB2, TPSAB1, CPA3, MS4A2, HDC
Neutrophils	32	7	0.48	FPR1, SIGLEC5, CSF3R, FCAR, FCGR3B, CEACAM3, S100A12
NK CD56dim cells	14	4	0.40	KIR2DL3, KIR3DL1, KIR3DL2, IL21R
NK cells	36	3	0.47	XCL1, XCL2, NCR1
T-cells	13	6	0.81	CD6, CD3D, CD3E, SH2D1A, TRAT1, CD3G
Th1 cells	27	1	^a^NA	TBX21
Treg	18	2	^a^NA	FOXP3
CD8 T cells	35	2	0.51	CD8A, CD8B
CD4 cells	20	0	^b^NA	

^a^Only one marker gene; quality impossible to assess in expression data alone

^b^Calculated as the T-cell score minus the CD8 cell score

Cell types lacking acceptable marker genes are omitted. The mean pairwise similarity statistic is a measurement of how well a gene set adheres to the co-expression patterns expected from a set of perfect marker genes, with a score of 1 indicating perfect marker-like behavior

**Fig. 2:**
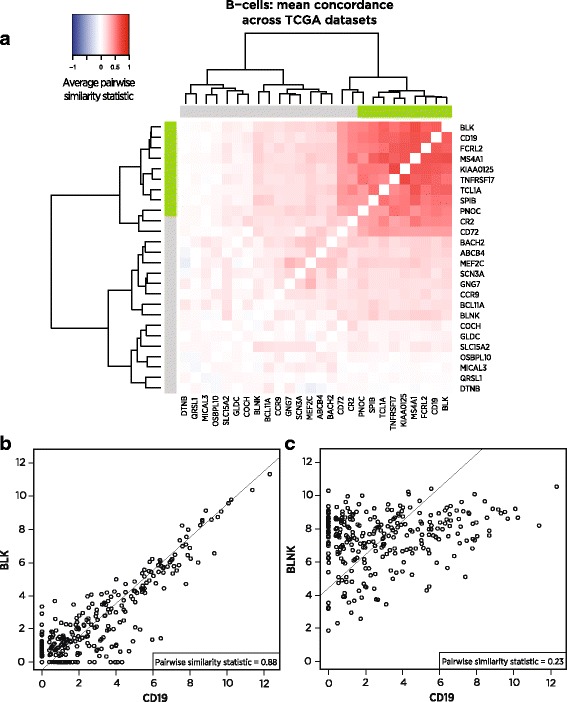
Pairwise similarity, a measure of marker-like co-expression, of candidate B-cell marker genes in TCGA. **a** Pairwise similarity of candidate B-cell marker genes averaged across 24 TCGA RNASeq datasets. Darker *red* indicates co-expression patterns consistent with both genes acting as cell type markers. Values of 1 indicate perfect marker-like co-expression. *Green* sidebars indicate final selected markers. **b** Two of the selected B-cell markers, including CD19, in the bladder cancer dataset, demonstrating strong marker-like co-expression. **c** In bladder cancer, CD19 and the rejected candidate marker BLNK, displaying co-expression inconsistent with both genes acting as B-cell markers

Figure 2 illustrates our selection process: of the 26 genes previously reported as being expressed specifically in B-cells, most have co-expression patterns incompatible with specificity to the same cell type. But a subset of genes, including the canonical B-cell marker CD19, share the co-expression patterns we seek, namely high correlation with a slope near 1 (Fig. 2a). For example, in the TCGA bladder cancer (BLCA) dataset, BLK and CD19 show nearly perfect marker-like co-expression (Fig. 2b), while the putative B-cell marker BLNK is largely uncorrelated with CD19 (Fig. 2c). BLNK’s unsuitability as a B-cell marker is corroborated by [21]’s finding of BLNK expression in murine macrophages.

In some cell types, prior biological knowledge informed our selection process. For example, we discarded a cluster of putative Th2 cell marker genes (BIRC5, HELLS, CDC7, WDHD1, CENPF, NEIL3, DHFR, DC25C) that showed strong marker-like co-expression (Additional file 3: Figure S16) but had many genes that were previously reported to be expressed broadly across cell types [22–24]. The strongest cluster among the Th1 cell candidate genes included genes known to be expressed in a variety of cell types, including IFNG and CTLA4. The strongest cluster in the iDC gene list had several genes known to be expressed in T cells, including CD1B, CD1C, and CD1E, which are associated with T cells. Similarly, the strongest cluster in our CD8 T cell candidate gene list included CD8A and CD8B, which had a very high pairwise similarity statistic (>0.7), but also FLT3LG and GZMM. These other two genes would have made our final list of markers based on their co-expression with CD8A and CD8B, but their known expression in other cell types [25, 26] led us to exclude them. These results suggest that while domain knowledge may be insufficient to identify marker genes on its own, it can serve as a useful referee for the discoveries of purely computational procedures.

**Fig. 3:**
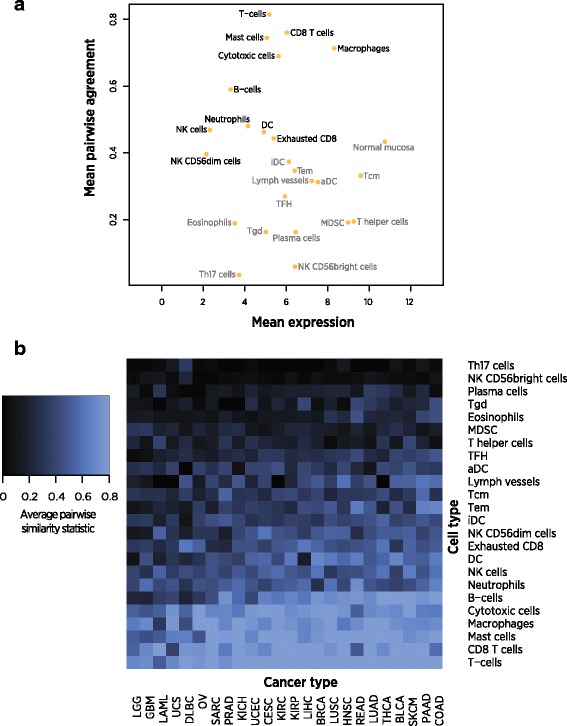
Pairwise similarity, a measure of marker-like co-expression, of selected marker genes in TCGA. Values of 1 indicate perfect marker-like co-expression. **a** Mean log2 expression vs. average pairwise similarity of selected cell type markers across TCGA datasets. Cell types in grey have been discarded from the final panel of markers. **b** Average pairwise similarity of each cell type’s marker genes in each TCGA dataset

Cell types varied in the quality of their selected marker genes (Fig. 3). For example, our selected set of T-cell genes showed very strong marker-like co-expression, while our selected T-helper cell genes displayed weak marker-like co-expression (Additional file 3: Figure S11). Genes with lower average expression were less likely to display expression patterns typical of ideal cell type markers, a pattern consistent with greater measurement error at low expression values. Noting this pattern, two clusters of cell types with respectively successful and unsuccessful marker genes are apparent (Fig. 3a). We discarded cells in the lower cluster, which we demarcated with an average pairwise similarity score threshold of 0.4. This threshold is arbitrary by necessity, as marker genes cannot be partitioned into “good” and “useless” categories but rather occupy a continuum of adherence to marker-like behavior. We additionally discarded the “Normal Mucosa” cell type, which was derived in a colon cancer study and has uncertain interpretation in other cancer types.

The wide range of average pairwise similarity statistics of our admitted cell types is consistent with variability in marker genes’ adherence to ideal marker-like behavior. For example, our T-cell markers’ co-expression pattern suggests they hew closely to the ideal of perfect cell type specificity and stability, while our NK cell markers’ weaker co-expression suggests they experience some degree of variable expression within NK cells, low-level expression in other cell types, or increased measurement error due to low expression.

In three cases, we retained a single gene as a cell type marker. For Th1 cells, we found no clusters of candidate genes with marker-like co-expression; thus we selected TBX21, the gene for the classic Th1 cell marker T-bet. However, [27] reported T-bet expression in B-cells, so this marker gene may be influenced by B-cell abundance. Our Treg candidate genes also lacked highly co-expressed clusters, and so we took FOXP3 as a single marker gene for Tregs. We also use PTRPC as an unvalidated, single-gene marker of CD45+ cells, although it is likely expressed at different levels by different cell types. Given the prominence in the literature of all three of these genes, they seem appropriate to include in a panel of marker genes, although our analysis can neither falsify nor provide additional evidence for their marker status.

The selected marker genes appear to have pan-cancer utility: each set of marker genes showed similar performance across TCGA datasets as measured by the pairwise similarity statistic (Fig. 3b). An important exception is brain and immune tumors, which showed reduced marker-like co-expression for all cell types. Poor performance in immune tumors might be expected to result from tumor-intrinsic expression of immune genes, and poor performance in brain tumors likely results from the blood–brain barrier limiting the dynamic range of immune cell abundance and thereby limiting our ability to resolve marker-like co-expression patterns.

### Comparison of gene expression cell type scores to flow cytometry and IHC

**Fig. 4:**
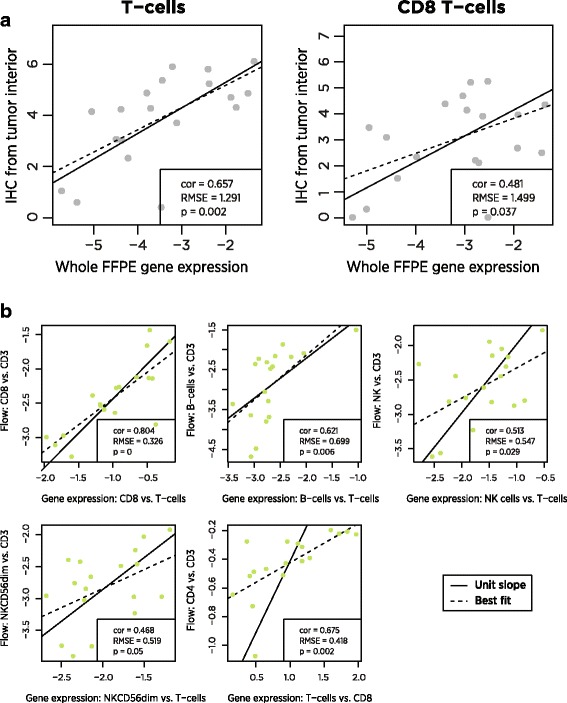
Comparison of gene expression cell scores to alternative biomarkers. **a** In FFPE tumor samples, gene expression cell scores and log2-transformed IHC measurements of cell type abundance. **b** In PBMC samples, gene expression and flow cytometry measurements, normalized to T cell abundance

FFPE tissue and PBMCs were collected from ovarian cancer patients. CD3+ and CD8+ cells were quantified in FFPE samples using IHC, and numerous cell populations (Additional file 1: Table S2) were quantified in PMBCs using flow cytometry. In 19 FFPE and 18 PBMC samples, we measured expression levels of our 60 cell type marker genes and of 670 additional genes relevant to the tumor-immune interaction. Gene expression cell type scores were broadly concordant with both flow cytometry and IHC measurements (Fig. 4, Table 2).

**Table 2:** Reproducibility and concordance with alternative cell type quantification methods

Cell type	Correlation with IHC	Root Mean Squared Error from IHC	Correlation with flow	Root Mean Squared Error from Flow	Mean pairwise similarity statistic in TCGA	SD due to technical noise (log2 scale)	Proportion of variance due to noise
B-cells			0.62	0.064	0.59	0.13	0.0022
CD45					^c^NA	0.1249	0.0024
Cytotoxic cells					0.69	0.0813	0.001
DC					0.46	0.2307	0.0151
Exhausted CD8					0.44	0.1624	0.0062
Macrophages					0.71	0.0828	0.0013
Mast cells					0.74	0.1949	0.0086
Neutrophils					0.48	0.19	0.0026
NK CD56dim cells			0.47	0.071	0.40	0.2347	0.1073
NK cells			0.51	0.118	0.47	0.1938	0.017
T-cells	0.66	1.3	^a^0.78	^a^0.064	0.81	0.1116	0.0021
Th1 cells					^c^NA	0.2212	0.0304
Treg					^c^NA	0.371	0.049
CD8 T cells	0.53	1.5	0.78	0.138	0.51	0.1842	0.0045
CD4 cells			^b^0.65	^b^0.752			

^a^Used to normalize the other cell types; 0.78 and 0.064 are the highest correlation and lowest RMSE observed between gene expression and flow for any T-cells vs. other cell type contrast

^b^Calculated as the T-cell score minus the CD8 cell score

^c^Only one marker gene; quality impossible to assess in expression data alone

Root mean squared errors are calculated from log2-scale abundance measurements. The mean pairwise similarity statistic measures how well a gene set’s co-expression pattern adheres to the co-expression pattern of ideal marker genes, with a value of 1 indicating perfect correlation with a slope of 1. The standard deviation (SD) and proportion of variance due to noise were calculated from triplicate gene expression assays from tumor sample RNA

For comparison of our cell scores to flow cytometry, the normalization of gene expression data in PBMCs required a non-standard method. Changes in the composition of PBMCs can influence the abundance of housekeeping/reference genes, spuriously changing normalized expression values and by extension our cell type scores. We avoided this problem by normalizing our cell type scores not to reference genes but to our T-cells score, which appears to be our most accurate score. This step removes any concern about housekeeping genes, as the contrast between any two cell type scores will be independent of each sample’s normalization factor. Several of our cell type scores lacked an exact counterpart in the flow cytometry data and thus could not be validated by this method.

A notable finding from the flow cytometry data is the ability of our cell type scores to predict CD4 abundance. Although we have no explicit CD4 cell score – our analysis of TCGA data cast doubt on the utility of all the reported T helper cell genes – the difference between our T cell and CD8 cell scores correlates (*r* = 0.65) with the difference between flow cytometry CD4 and CD3 log2 counts.

The between platform correlations in cell type measurements were in general moderately strong but statistically significant. The near-unit slopes of the lines of best fit in these plots is important: these slopes mean that a 2-fold increase in T-cells as measured by gene expression predicts a 2-fold increase in T-cells as measured by IHC. Note that each platform returns results on a different scale, and so it is necessary to mean-center their measurements before comparing them.

The low reproducibility of IHC measurements [28] and the variable spatial distribution of immune cells within a tumor sample place strong upper bounds on the correlation between IHC and gene expression measurements of cell type abundance. For example, spatial sampling factors appear to explain the low outlier in Fig. 4: in this IHC sample, CD3 and CD8 cells were nearly absent from the tumor interior but were highly abundant in the invasive margin.

The correlation between gene expression and flow cytometry is limited by the relatively constant proportions of immune cell populations in PBMCs. The most variable comparison, CD4 cells vs. CD3 cells, changed by less than 30% between its minimum and maximum. In contrast, IHC T-cell measurements increased by 20-fold between their minimum and maximum. The very small root mean squared errors (RMSE) between gene expression and flow measurements are consistent with high concordance but low variance. Further discordance between gene expression and flow cytometry can be attributed to measurement errors in both platforms, gating decisions in flow analysis, and genuine differences in the biology captured by the two platforms.

### Reproducibility of gene expression cell type scores

**Fig. 5:**
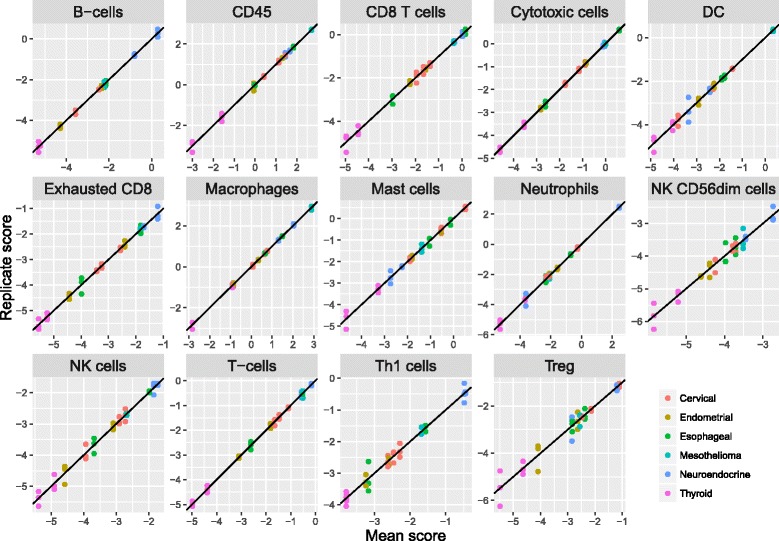
Reproducibility of cell scores derived from triplicate runs of 12 tumor samples. For each cell type, each sample’s individual replicate cell scores are plotted against its average score. Color denotes tumor type

To evaluate our cell scores’ technical reproducibility, we assayed RNA extracted from 12 tumor FFPE samples (Asterand) in triplicate using the nCounter PanCancer Immunology Panel (NanoString Technologies). These 12 samples included 2 endometrial carcinomas, 3 cervical carcinomas, 2 thyroid carcinomas, 2 neuroendocrine carcinomas, 2 esophageal tumors, and 1 mesothelioma. Reproducibility for most cell scores was extremely high (Fig. 5, Table 2), with the median cell score having a negligible 0.5% of variance explained by technical noise. Our NK CD56 Dim cell score had notably worse reproducibility than the rest, with 10% of variance due to noise.

### Application of cell type marker genes to TCGA RNASeq data

#### Results in TCGA: pan-cancer patterns in TIL abundance

**Fig. 6:**
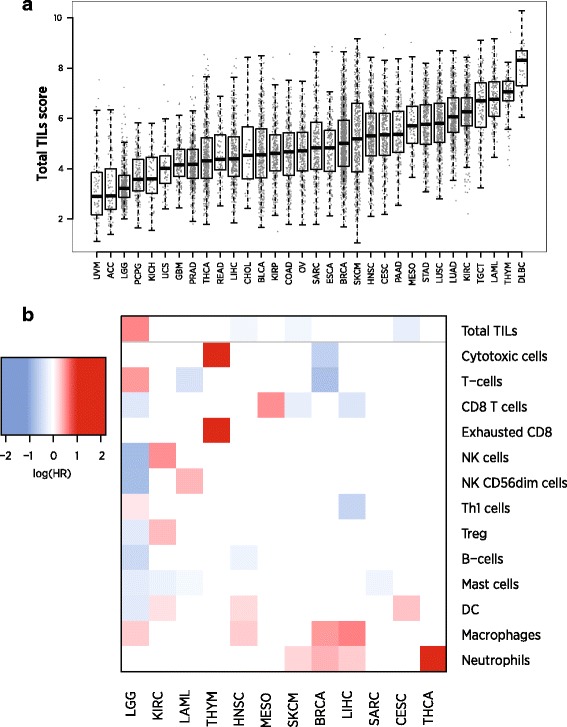
Results of analyses of cell scores in TCGA RNASeq data. **a** Boxplot of Total TILs score across TCGA datasets. Datasets are ordered according to median score. The vertical axis is log2-scale. **b** Prognostic information in Total TILs score and in cell type enrichment scores, the residuals of each cell score when regressed on the Total TILs score. *Red* indicates cell types whose enrichment within the total infiltrate is associated with poor outcome; *blue* indicates association with good prognosis. Only results with FDR < 0.05 are shown, and cancers without any statistically significant cell types are not shown

We used our immune cell marker genes to calculate cell abundance scores in 9986 TCGA RNASeq samples from 24 tumor types. The majority of immune cell scores tended to rise and fall together, with the average pair of cell scores having a correlation of 0.61 over the solid tumor TCGA datasets. This finding suggests that the primary component of variance in most cell types’ abundance is driven by the amount of infiltrate rather than its makeup. To capture this primary axis of information, we defined a “Total TILs” signature as the average of all cell scores with correlations with PTRPC (CD45) greater than 0.6, which excluded only dendritic cells, Tregs and mast cells. Our Total TILs score explained 60% of the variance in our cell scores in TCGA data. Total TILs score varied widely between and within tumor types (Fig. 6a).

#### Results in TCGA: prognostic significance of cell types

In each TCGA dataset, we tested the prognostic utility of Total TILs score and of each cell type’s enrichment or depletion relative to Total TILs. We first defined cell type enrichment scores as the residuals of linear regressions predicting each cell type from our Total TILs score. These cell type enrichment scores do not measure absolute abundance of a cell type but rather its enrichment or depletion within the immune infiltrate. We found cell type enrichment scores to provide a more interpretable picture of this data than our raw cell scores, whose high correlation concealed their distinct prognostic relevance in univariate analyses. We ran univariate Cox regressions predicting survival in each dataset from these cell type enrichment scores (Fig. 6b.) Eleven tumor types had statistically significant (FDR < 0.1) [29] associations between survival and at least one feature of TIL abundance and makeup.

As others have shown [3, 30], we see that immune cell populations have different prognostic implications in different tumor types, though some patterns are apparent. High Total TILs score predicts longer survival in melanoma (SKCM) and head and neck (HNSC) tumors but worse prognosis in lower grade gliomas (LGG) and kidney renal clear cell tumors (KIRC). Enrichment of T-cells, CD8 T-cells and mast cells also tends to predict good prognosis. Enrichment of DCs, neutrophils and macrophages generally indicates poor prognosis, suggesting that these cell types mount a less effective immune response or can serve as suppressor cells.

The melanoma (SKCM) results best match the standard theory of immunotherapy: increased TILs and an infiltrate enriched for CD8 T-cells and Th1-induced IFN-gamma signaling indicate an effective immune response. The glioma (LGG) and kidney renal clear cell carcinoma (KIRC) results are striking: overall TIL abundance is associated with shorter survival, and most individual immune cell populations hold further prognostic importance. The LGG results can be explained by the danger of inflammation in the brain and by the role of macrophages in suppressive signaling. The thymoma (THYM) results are also interesting: although Total TILs score is not prognostic, there is rich prognostic information in the enrichment of various cell populations within the total infiltrate.

Of the 21 tumor types without evidence for a prognostic role of TILs, 10 lacked statistical power, with fewer than 33 events. The BLCA, CESC, COAD, ESCA, GBM, KIRP, LUAD, LUSC, OV, PAAD and STAD datasets all had at least 49 events, but lacked evidence for a prognostic role of TILs. The negative result in the colon cancer (COAD) dataset is a notable divergence from the prognostic relevance of the Immunoscore [2], though with only 49 events the dataset had modest power to establish an association.

Section S2 in the Additional file 2 contains further analyses of our cell scores in TCGA, including analyses of total immune abundance across tumor types, immune cell co-occurrence, correlation between immune populations and key immune oncology genes, relationships between tumor type and the makeup of the immune infiltrate, and associations between mutation burden and total immune infiltrate.

### Application of cell type marker genes to an immunotherapy dataset

A recent clinical trial collected biopsies from melanoma patients through checkpoint inhibitor therapy, first before and during anti-CTLA4 therapy and then before and during anti-PD1 therapy [31]. We calculated cell type scores using publically available NanoString data from 54 of these biopsies. As the study only included 22 of our cell type marker genes, we calculated each cell type’s score as the average of its available marker genes. Additional file 1: Table S6 details which marker genes were available.

**Fig. 7:**
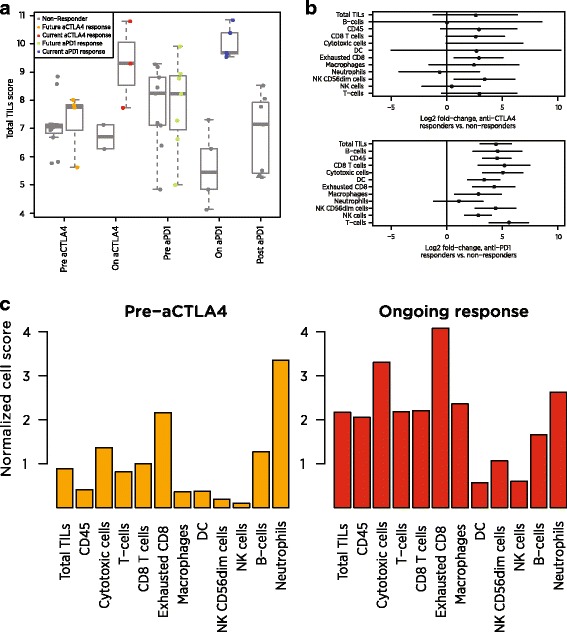
Application of cell scores to an immunotherapy dataset. **a** Total TILs score of each biopsy at each timepoint. Total TILs score was calculated as the average of all cell scores with >0.6 correlations with CD45, a metric that excluded only NK cells and neutrophils. Grey points denote non-responders; colored points denote responders. **b** Estimates and 95% confidence intervals for each cell score’s log2 fold-change between responders and non-responders on anti-CTLA4 (top) and anti-PD1 (bottom). **c** Cell scores from a single anti-CTLA4 responder before and during therapy. Scores are given as log2 fold changes from the average patient’s pre-treatment score

Our Total TILs score mirrored the original study’s conclusions. Before treatment, responders and non-responders had similar scores. During anti-CTLA4 treatment, the average responder had a 2.58-unit increase in Total TILs score relative to the average non-responder, which can be interpreted as a 6-fold increase in total TILs abundance (since 2^2.58^ = 5.97). During anti-PD1 treatment, the average responder’s score was 4.41 units higher than the average non-responder’s, equivalent to a 21-fold difference in TIL abundance (Fig. 7a). These on-treatment biopsies were taken before clinical benefit was evident, suggesting our Total TILs score could provide an early measure of checkpoint inhibitor success.

Examination of individual cell scores shows that the individual cell populations do not all increase equally during clinical response (Fig. 7b). In patients on anti-CTLA4, most cell scores were between 2.4 and 3.4 units higher in responders than in non-responders, indicating 5 to 11-fold increases in cell abundance. Exceptions from this trend were B-cells, NK cells and neutrophils, which showed negligible associations with response. Under anti-PD1 therapy, most cell types were more than 4.2 units higher (18-fold more abundant) in the average responder than in the average non-responder, and neutrophils were the only cell type that was not statistically significantly associated with response. Unlike anti-CTLA4 responders, anti-PD1 responders showed increased NK cell abundance and greatly increased (>20-fold) B-cell abundance. Figure 7c demonstrates the application of our scores to pre- and on-treatment samples from a single patient. To highlight how this patient compares to the broader population, we report each cell score relative to its average in the immunotherapy-naïve samples at the pre-CTLA4 timepoint. All cells but neutrophils become more abundant during the patient’s clinical response, with the greatest increases seen in the T cell lineage, macrophages and cytotoxic cells.

## Discussion

It is unknown whether our cell type scores track pure cell type abundance like flow and IHC or whether they track the product of cell type abundance and activity. For example, our data cannot rule out the possibility that highly active CD8 cells have increased expression of our CD8 marker genes relative to inactive CD8 cells. Whether cell type abundance or cell type activity levels have greater clinical relevance cannot be assessed by the data in this study.

CD4 subpopulations appear to lack sufficiently specific marker genes: for neither CD4 cells nor for any CD4 subpopulation did we find co-expression patterns among candidate genes consistent with cell type specificity. Quantification of these populations may require a deconvolution approach such as that employed by [9]. The Additional file 2 section S3.1 contains a discussion of the relative merits of deconvolution methods versus the marker gene approach we have taken. Alternatively, CD4 population functions could be measured with their canonical genes, similar to our use of TBX21 to measure Th1 cells and FOXP3 to measure Tregs.

Normalizing gene expression data for application of this method becomes complicated in non-tumor samples like PBMCs and cultured cells. In PBMCs, for example, it is likely that the standard reference genes have different expression levels in different cell types; thus a PBMC sample with abundant T-cells might be normalized to a different level than a PBMC sample with depleted T-cells. A workaround to this problem is to normalize not to reference genes but to a single immune cell population, yielding relative measurements of cell types like CD8 cells/CD3 cells and B-cells/CD3 cells. We apply this approach to our analysis of a flow cytometry dataset. Alternatively, normalizing to the average score of the major PBMC components – T-cells, B-cells, NK cells and macrophages – approximates normalization to the total number of cells in a sample.

The quality of the list of candidate markers is crucial to the success of our method and the derivation of our gene list. We seeded our analysis with candidate markers derived from prominent meta-analyses of separated immune cell populations. It is likely that future datasets and meta-analyses will support superior candidate gene lists which could be used to derive additional markers, including markers of cell types for which our analysis was unsuccessful.

We find in all our analyses that the immune cell populations we measure are correlated, rising and falling together. Nonetheless, our cell scores’ distinct prognostic significance in TCGA in Fig. 6b and their differential responses to checkpoint inhibition in Fig. 7 emphasize that the cell scores provide non-redundant information and that analysis of multiple scores returns more detailed information than a simple measure of total TILs.

To aid other investigators, we provide R code for calculating cell type abundances and for performing QC on marker genes in new datasets. We also list the candidate genes we examined (Additional file 1: Table S3) and the selected genes (Additional file 1: Table S4), and we provide cell type abundance scores on 9986 TCGA samples (Additional file 4: Table S5). All data and code used in these analyses are available as Additional file 5. NanoString Technologies has implemented this immune cell scoring method in a free, open-source analysis tool.

## Conclusions

We have identified a set of marker genes with sufficient cell type specificity that their expression levels can be used to measure immune cell subpopulations in the tumor microenvironment. The quality of available marker genes varies across cell types (Fig. 3, Table 2), with some cell populations (T-cells, cytotoxic cells, mast cells, macrophages) having many well-behaved markers with very high pairwise similarity statistics, with other cell populations possessing only weak markers (exhausted CD8 cells, NK CD56 dim cells), and others lacking any suitable markers (Th17 cells, CD4 cells). Similarly, our 14 cell type gene lists have different levels of evidence. Our T cell and CD8 cell scores have the highest level of evidence: they correlate well with both IHC and flow, and their marker genes show strong marker-like behavior in TCGA data. We lack IHC data that could validate our B, NK, and NK CD56dim scores, but these cell scores correlate with flow cytometry and their markers behaved approximately as well in TCGA as our CD8 cell markers. Our mast cell, cytotoxic cell and macrophage scores have neither IHC nor flow measurements to support them, but their marker genes exhibited very strong marker-like co-expression in TCGA. Finally, our neutrophil and exhausted CD8 cell markers performed approximately as well in TCGA as our CD8 cell markers.

The immune cell scores described here can be implemented in a single assay using any gene expression platform, and any single cell type score can be calculated with an assay of just a handful of genes. Thus these cell scores represent a convenient technique for extracting detailed information about the tumor immune contexture in samples and settings where flow cytometry is unavailable. Furthermore, given their demonstrated prognostic value in TCGA, their association with clinical response to checkpoints in the data of [31], and the increasingly well-understood associations between immune populations and response to immunotherapies [6], these cell scores may hold information useful for monitoring or predicting response to immunotherapy.

## Additional files

Additional file 1: Tables S1-S4, S6. Legend: Tables detail 1) the TCGA download used in our analyses, 2) the markers analyzed in flow cytometry, 3) the candidate marker genes we derived from the literature, 4) the marker genes we ultimately selected, and 5) the genes present in the immunotherapy dataset of [31]. (XLSX 26 kb)

Additional file 2: Supplementary methods, results, and discussion. Legend: Supplementary material: Gene expression markers of Tumor Infiltrating Leukocytes. (PDF 1 kb)

Additional file 3: **Figure S10.** Tem: mean concordance of cadidate cell type markers across TCGA datasets. **Figure S11.** T helper cells: mean concordance. **Figure S12.** Macrophages: mean concordance. **Figure S13.** MDSC: mean concordance. **Figure S14.** Tcm: mean concordance. **Figure S15.** NK cells: mean concordance. **Figure S16.** Th2 cells: mean concordance. **Figure S17.** B − cells: mean concordance. **Figure S18.** Neutrophils: mean concordance. **Figure S19.** Th1 cells: mean concordance. **Figure S20.** Normal mucosa: mean concordance. **Figure S21.** iDC: mean concordance. **Figure S22.** aDC: mean concordance. **Figure S23.** DC: mean concordance. **Figure S24.** Eosinophils: mean concordance. **Figure S25.** Tgd: mean concordance. **Figure S26.** T − cells: mean concordance. **Figure S27.** Exhausted CD8: mean concordance. **Figure S28.** CD8 T cells: mean concordance **Figure S29.** Mast cells: mean concordance. **Figure S30.** Treg: mean concordance. **Figure S31.** Cytotoxic cells: mean concordance. **Figure S32.** TFH: mean concordance. **Figure S33.** NK CD56bright cells: mean concordance. **Figure S34.** SW480 cancer cells: mean concordance. **Figure S35.** NK CD56dim cells: mean concordance. **Figure S36.** Th17 cells: mean concordance. **Figure S37.** Lymph vessels: mean concordance. **Figure S38.** Plasma cells: mean concordance. (PDF 949 kb)

Additional file 4: Table S5. Legend: cell type scores calculated in 9986 TCGA RNASeq samples. (CSV 2618 kb)

Additional file 5: All code and data. (ZIP 493984 kb)

## Abbreviations

CD: Cluster of differentiation
DC: Dendritic cell
IHC: Immunohistochemistry
NK: Natural killer
PBMCs: Peripheral blood mononuclear cells
RMSE: Root mean squared error
SD: Standard deviation
TIL: Tumor infiltrating leukocyte
