# Supplementary material for: Gene expression markers of Tumor Infiltrating Leukocytes
Source: J Immunother Cancer. 2017 Feb 21;5:18. doi: 10.1186/s40425-017-0215-8 (PMC5319024; doi:10.1186/s40425-017-0215-8)
Supplement: Supplementary file 2 — Legend: Supplementary material: Gene expression markers of Tumor Infiltrating Leukocytes. (PDF 1 kb) [file 40425_2017_215_MOESM2_ESM.pdf]

Supplementary material: Gene expression markers of Tumor Infiltrating Leukocytes

S1. Supplemental methods

S1.1. Algorithm for screening marker genes in novel tumor expression data

In any new tumor dataset, there is a risk that our cell type marker genes will fail to measure their putative cell types due to unexpected biology like tumor-intrinsic expression. Fortunately, the same co-expression patterns we used to validate our genes in TCGA can be applied to confirm their marker-like behavior in a new dataset. We use the below algorithm to identify and remove poorly-performing marker genes in new datasets.

1) *Assume there are p genes for a given cell type and n samples. Compute a p*p matrix of similarity scores between the genes. Each gene has similarity of 1 with itself.*
2) *Label all gene pairs with similarity below 0.2 as "discordant."*
3) *Iteratively remove genes: while there are more than 2 genes remaining and while at least one discordant pair of genes remains:*
    a. *Count the number of discordant pairs each gene participates in. Call the maximum of these counts max_discord.*
    b. *Identify the genes with max_discord instances of discordance with another gene. Of these genes, remove the single gene with the lowest average similarity to the other remaining genes.*

S1.2. Calculation of p-values for cell type gene sets

In each new dataset, it is desirable to confirm that our cell type marker genes continue to display cell type specific and stable expression. To assess the utility of a set of marker genes in new data, we apply a permutation test to evaluate whether their marker-like co-expression exceeds that of a random gene list. We first define the below metric of a gene set's marker-like co-expression, or its adherence to the assumption of cell type-specific and consistent expression:

$$concordance(X) = \frac{1}{trace(Cov(X))}\left(p^{-\frac{1}{2}}, \dots, p^{-\frac{1}{2}}\right) Cov(X) \left(p^{-\frac{1}{2}}, \dots, p^{-\frac{1}{2}}\right)^T,$$

where *X* is the matrix of log-transformed, normalized expression values of the gene set, and where *p* is the number of genes. The *concordance()* function evaluates at 1 if all genes are perfectly correlated with a slope of 1, and degrades to 0 as this pattern weakens. The *concordance()* function can also be thought

of as measuring the proportion of variance in a gene set explained by the average expression of its genes.

We perform our permutation test as follows. Assume the given gene set has p genes, of which p_0 survived the iterative gene selection procedure. Call the data from the gene set X, and the data from the reduced gene set X_0.

1) Compute *concordance*(X_0).
2) Choose 1000 random genes sets of size p. Denote the data from a random gene set X'.
3) For each gene set, use the gene selection algorithm to reduce X' to only its best p_0 genes. Call the data from this reduced random gene set X_0', and compute *concordance*(X_0').
4) Return a p-value equal to the proportion of *concordance*(X_0') values greater than *concordance*(X_0).

S1.3. Extraction of RNA from FFPE tissue

For each FFPE sample, RNA was extracted from 3 tissue curls using the Qiagen RNeasy FFPE Kit (50). Samples were deparafinized with 1mL of citrisolve (cat. #1601), vortexed and then centrifuged at ~21000 xg. Supernatant was removed, and 1mL ethanol was added to the pellet. The resulting solutions was spun that down at full speed and its supernatant was discarded. The samples were held at 37°C until the ethanol evaporated. Samples were then allowed to cool, after which 240uL of Buffer PKD was added. Samples were then vortexed and then centrifuged for 1 min @ 11k xg. Finally, 10uL of proteinase K was added, and samples were incubated at 56°C overnight.

Samples were placed at 80°C for 15 minutes to kill the ProK reaction. The samples were immediately transferred to a 2mL Eppendorf tube and placed on ice for three minutes. Next, samples were centrifuged at 20k xg for 15 minutes at room temp. The supernatant was kept and transferred to a fresh tube where 25uL of DNase booster and 10uL of DNase I was input. After a few inversion mixes the samples were left to incubate at room temperature for 15 minutes. 500uL of Buffer RBC was added to the samples and mixed. Then 1200uL of ethanol 100% was added and then mixed the sample. 700uL of that solution was moved to a new column then spun at room temp 15s at 8k xg. The last batch was spun through the column and discarded then added 500uL of RPE and spun at 8k xg for fifteen seconds. The RPE step was then repeated and the flow through was discarded along with the old collection tube. The column was immediately placed onto a new collection tube and spun at full speed and room temperature for 5 minutes. After completion the column was put on a new Eppendorf tube, and 30uL was added to the top of the column and spun down full speed for 1 minutes.

S2. Supplemental results

S2.1. Results in TCGA: co-enrichment patterns of cells in the immune infiltrate

To reveal subtler patterns not driven by total TIL abundance, we fit linear regressions predicting each cell score from the total TILs score, and we analyzed the residuals of these regressions. These residuals measure each cell score's enrichment or depletion relative to what would be expected given the total TILs score; that is, they describe the makeup rather than the amount of the immune infiltrate. Figure S1 displays a correlation matrix of these cell type residuals. Enrichment of the potentially suppressive neutrophil and macrophage populations was negatively correlated with enrichment of the T, NK and Cytotoxic cell populations. Treg and CD8 T-cell residuals were uncorrelated, suggesting that a high proportion of Tregs in the infiltrate does not influence the proportion of CD8s, or vice versa.

Figure S1: Correlation matrix of cell types' enrichment relative to total infiltrate. Orange indicates pairs of cell types that are co-enriched; blue indicates co-depletion. The color key is truncated at +/-0.5.

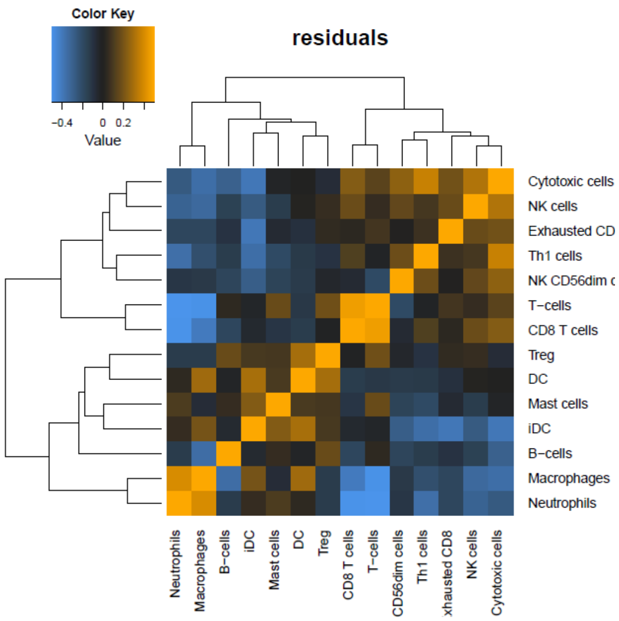

S2.2. Results in TCGA: associations between cell types and prominent immune-oncology genes

Immune cell abundance scores can give context to analyses of other genes involved in the tumor-immune interaction. For each cell type and for 35 genes important in immune oncology, we fit linear regressions predicting cell score from the gene's expression, adjusting for total TILs and for tumor type

(Figure S2). These regressions produced estimates of the association between each gene and each cell score beyond what could be explained by the influence of total TILs. Much expected and some unexpected biology emerged. Genes associated with enrichment of cells involved in adaptive immunity tended to predict depletion of innate immunity cells, and vice versa. Interferon-gamma and cytotoxicity genes predicted enrichment of adaptive cell types, with the exception of a negative association between cytotoxicity genes and Tregs. T cell costimulatory/genes split into two classes: HAVRCR2 (TIM3), C10orf54 (B7-H5), VTCN1 (B7-H4) and CD28 predicted enrichment of innate cell types, while PDCD1 and LAG3 predicted enrichment of adaptive cell types. Costimulatory genes and genes involved in NFκB signaling (HAVCR2, C10orf54, VTCN1, CD28, ICOS, ICOSLG, CD80, NFKB1, TNF, IL6, IL1A, IL12A, IL1B, CASP1, CASP4, CASP5, TGFB1) predicted enriched innate immunity cells. The two inflammation genes we considered had opposing patterns: IL15 predicted enrichment of NK and cytotoxic cells, while IL1B predicted enriched DCs, macrophages and neutrophils. VEGFA and VEGFB, genes involved in infiltration, were not notably associated with either innate or adaptive cell types. The checkpoint molecules IDO1, CTLA4 and CD274 (PDL1) had distinct patterns: IDO1 predicted adaptive cell enrichment, CTLA4 strongly predicted enriched Tregs, and PDL1 predicted enriched macrophages and neutrophils, slightly depleted CD8 T-cells, and slightly enriched Exhausted CD8 cells.

Supplemental Figure S2: Association between cell scores and immune genes. Orange cells indicate positive association after adjusting for total TILs and tumor type; blue indicates negative association

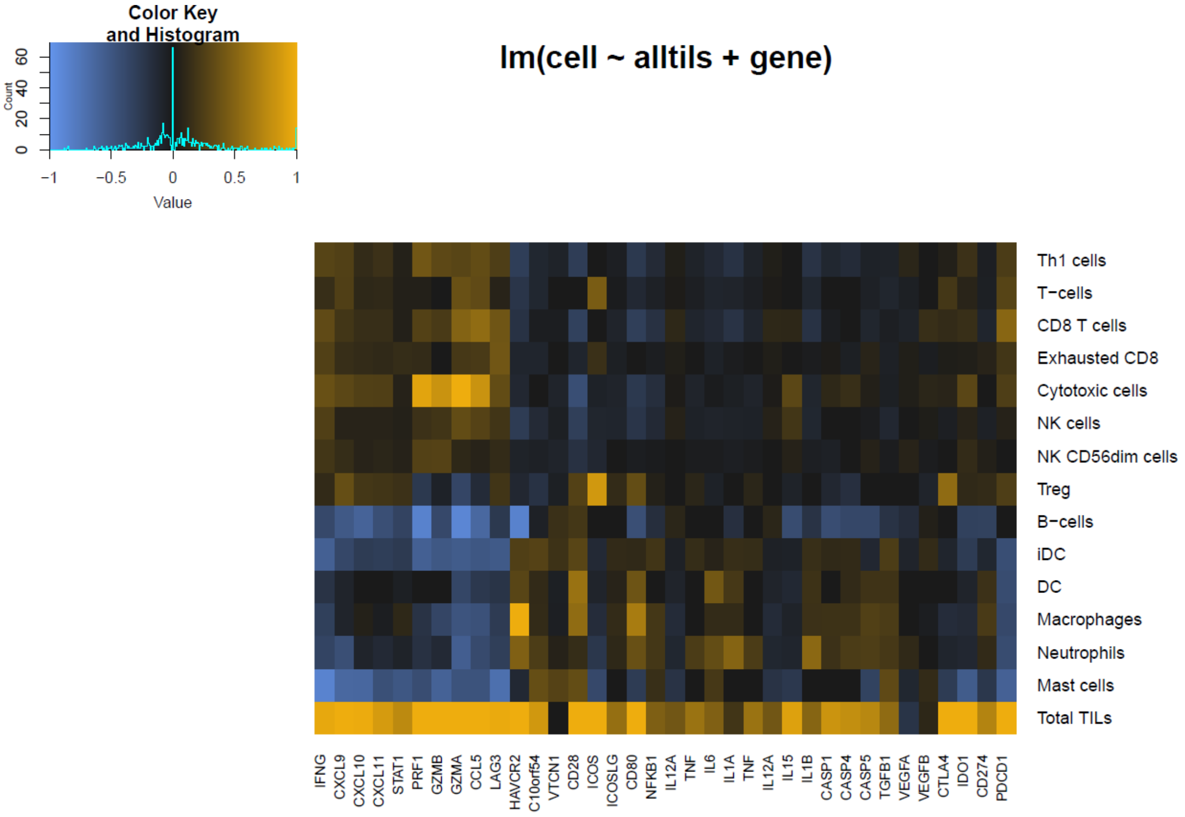

S2.3 Differential TIL landscapes across tumor types

We sought to measure how the composition of the immune infiltrate differs between tumor types. For each cell and each tumor type, we used all our TCGA solid tumor data to fit a linear regression predicting cell score from our total TILs score and from an indicator variable for the tumor type (Figure S3). These regressions allow us to identify cancers in which a cell type has an unusually large or small proportion of the immune infiltrate. Our Treg score displayed the most tumor type dependence. Treg score was suppressed in all 3 kidney cancers measured (KICH, KIRC, KIRP) and unusually high in head and neck cancer (HNSC). Examination of hepatocellular carcinomas (LIHC) revealed a cluster of patients with extremely high Treg score, suggesting Treg abundance up to 250-fold greater than would be predicted from other immune cells (Figure S4.) Other notable results include consistently high Exhausted CD8 scores in uveal melanoma (UVM), low macrophage scores in cervical squamous cell carcinoma (CESC), and high DC scores in sarcomas (SARC) and hepatocellular carcinomas (Figure S5). Our mast cell score displayed considerably greater tumor type dependence than our other cell scores.

Figure S3: Associations between cell scores and tumor type. The heatmap summarizes the results of all cell score vs. tumor type tests after adjusting for total TILs score.

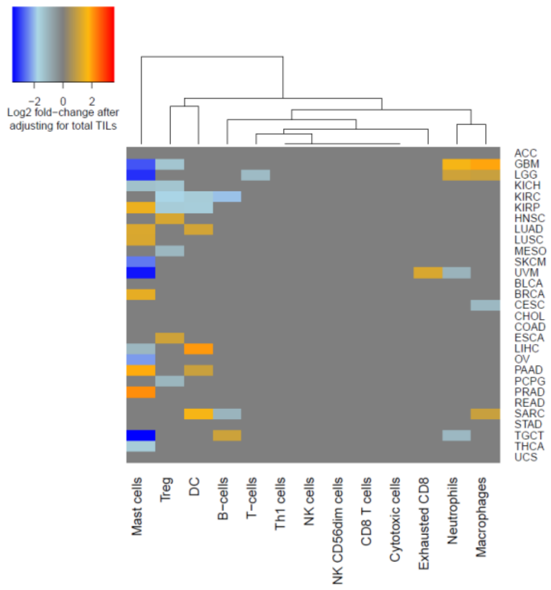

Figure S4: Treg score plotted against total TILs score for all TCGA solid tumors, with liver and kidney cancers highlighted.

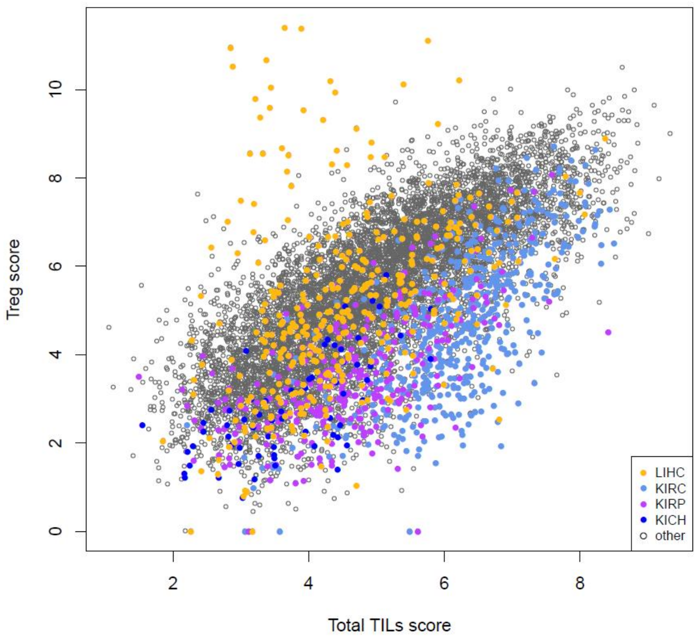

Figure S5: Enrichment of selected cell types in selected TCGA datasets. a) Exhausted CD8 cells plotted vs. total TILs, with uveal melanoma samples highlighted. b) Macrophages vs. total TILs, with cervical squamous carcinoma samples highlighted. c) Dendritic cells vs. total TILs, with sarcoma samples highlighted. d) Dendritic cells vs. total TILs, with hepatocellular carcinoma samples highlighted.

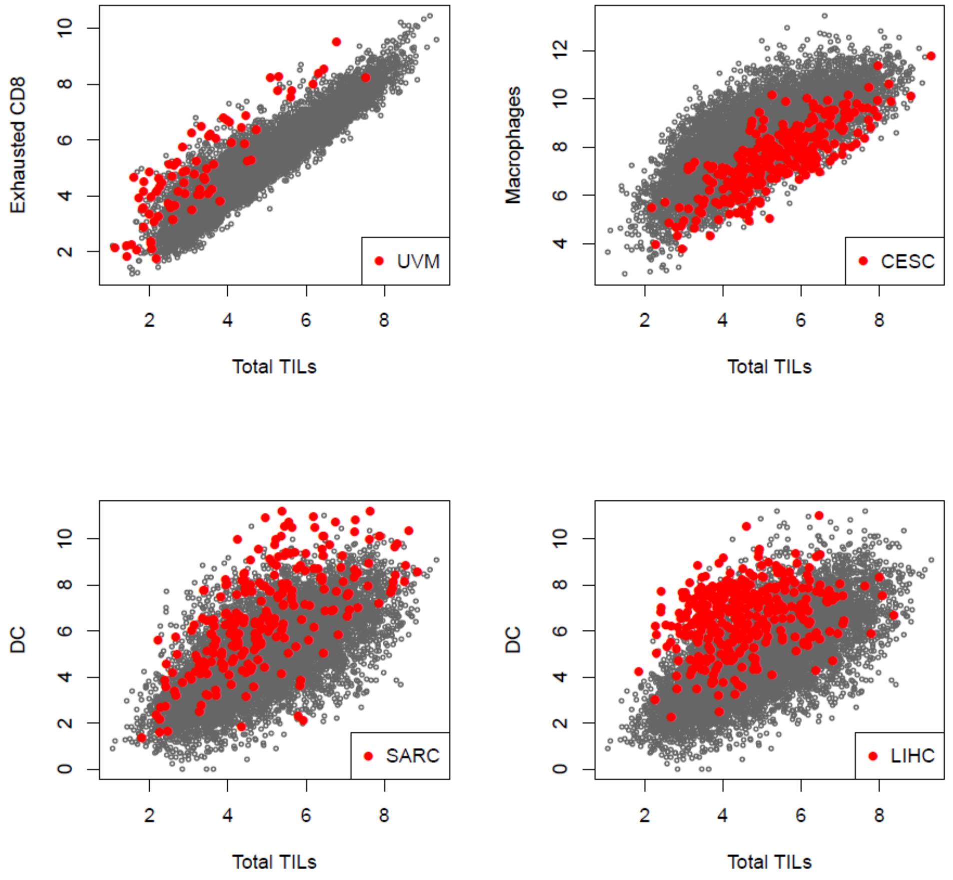

S2.4. Limited association between mutation burden and TIL abundance

A tumor's immunogenicity depends on the number of neoantigens it presents [1]. Thus a tumor's mutation burden might serve as a useful predictor of immunotherapy response. We examined the relationship between mutation burden and TIL abundance in a number of tumors and found that mutation burden explains little of the TIL response. In melanoma (SKCM) and lung adenocarcinoma (LUAD), mutation load was entirely uncorrelated with TIL abundance, perhaps because TIL abundance in these neoantigen-rich tumors is driven by suppressive factors like expression of immune checkpoints rather than availability of neoantigens. Kidney chromophobe (KICH) tumors had low mutation burdens and low TIL levels, though no correlation between these quantities was detectable. Colon adenocarcinomas (COAD) presented a more interesting case: while a low-mutation burden cluster of patients had a wide range of TIL abundance, a cluster of highly mutated (presumably microsatellite unstable) tumors always had at least moderate TIL abundance (Figure S6). Although this finding was statistically significant, most variability in colon tumor TIL abundance cannot be explained by mutation burden alone.

Figure S6: Mutation burden vs. total TILs score in 4 selected tumor types: melanoma, colon, lung adenocarcinoma, and kidney chromaphobe.

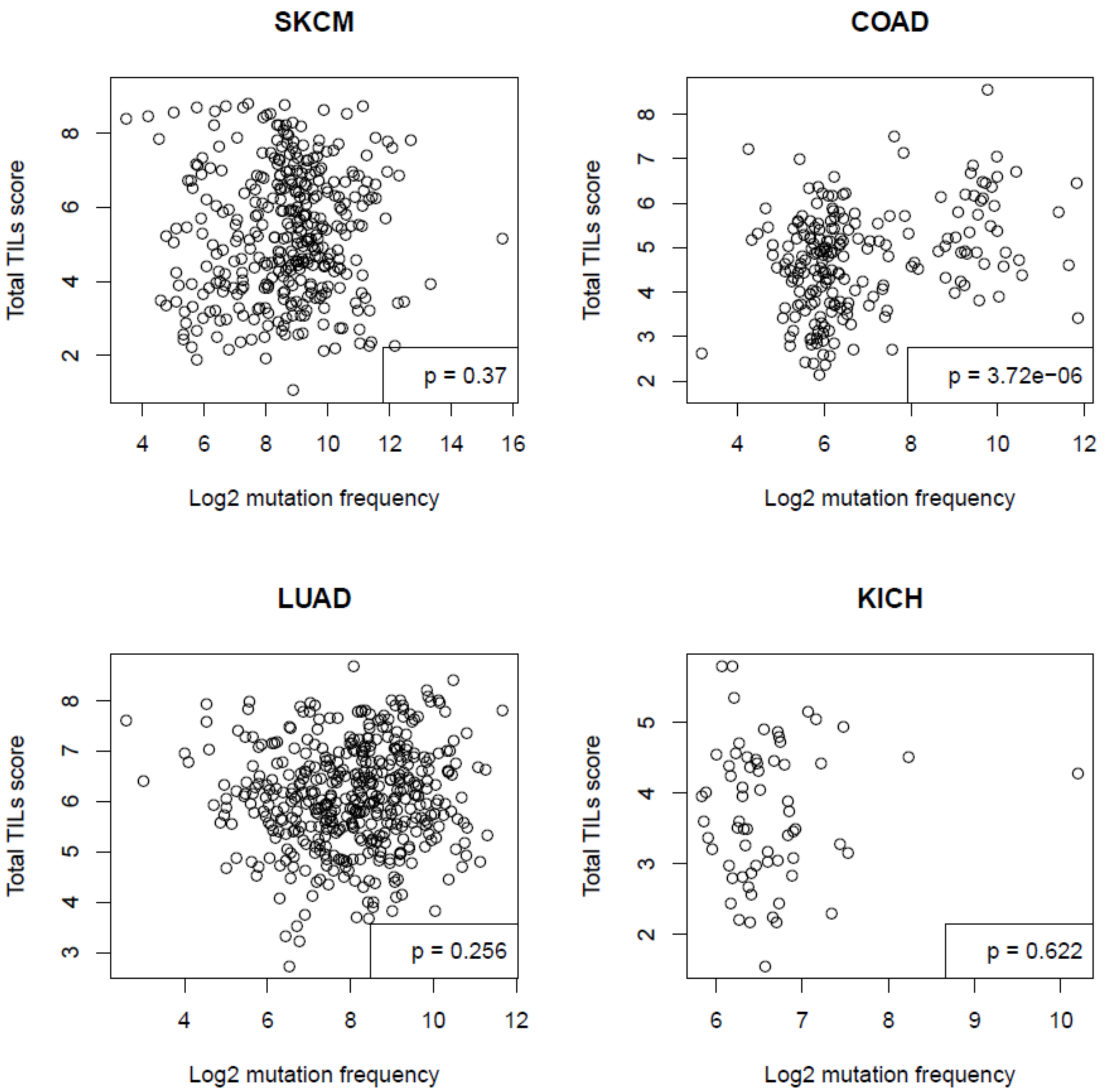

S2.5. Proof that two ideal marker genes will have a slope of 1 in the log space

Call x1 and x2 the expression levels of two genes, and call C the abundance of the cell type in which they are expressed. Assume both marker genes are ideal, namely they are expressed only in one cell type and they are expressed with perfect consistency in that cell type. Call b1 and b2 the number of copies/cell of each gene. Then, $x1 = b1 \cdot C$, and $x2 = b2 \cdot C$. Then $\log(x1) = \log(b1) + \log(C)$, and $\log(x2) = \log(b2) + \log(C)$. Then $\log(x1) = \log(b1) - \log(b2) + \log(x2)$. Therefore the slope between x1 and x2 equals 1.

S2.6. Simulation comparing the pairwise similarity statistic to simple correlation

To confirm that our pairwise similarity statistic is better than Pearson correlation at screening out genes that are correlated but with a slope incompatible with marker status, we ran the following simulation. We simulated 1000 abundance values of a hypothetical cell type. We then simulated data from 100 marker genes that were highly correlated with cell abundance with a slope of 1, and data from 100 genes that were equally correlated with the cell abundance but with non-unit slope. We then calculated a similarity matrix between all the simulated genes using Pearson correlation and using our pairwise similarity statistic (Figures S7,S8). Under the pairwise similarity statistic, the true marker genes form an easily identifiable cluster, whereas the Pearson correlation similarity matrix cannot separate the true markers from the spuriously correlated genes.

Figure S7: Similarity matrices of 100 simulated markers and 100 simulated false markers, with distance defined by Pearson correlation. Green sidebars indicate true markers.

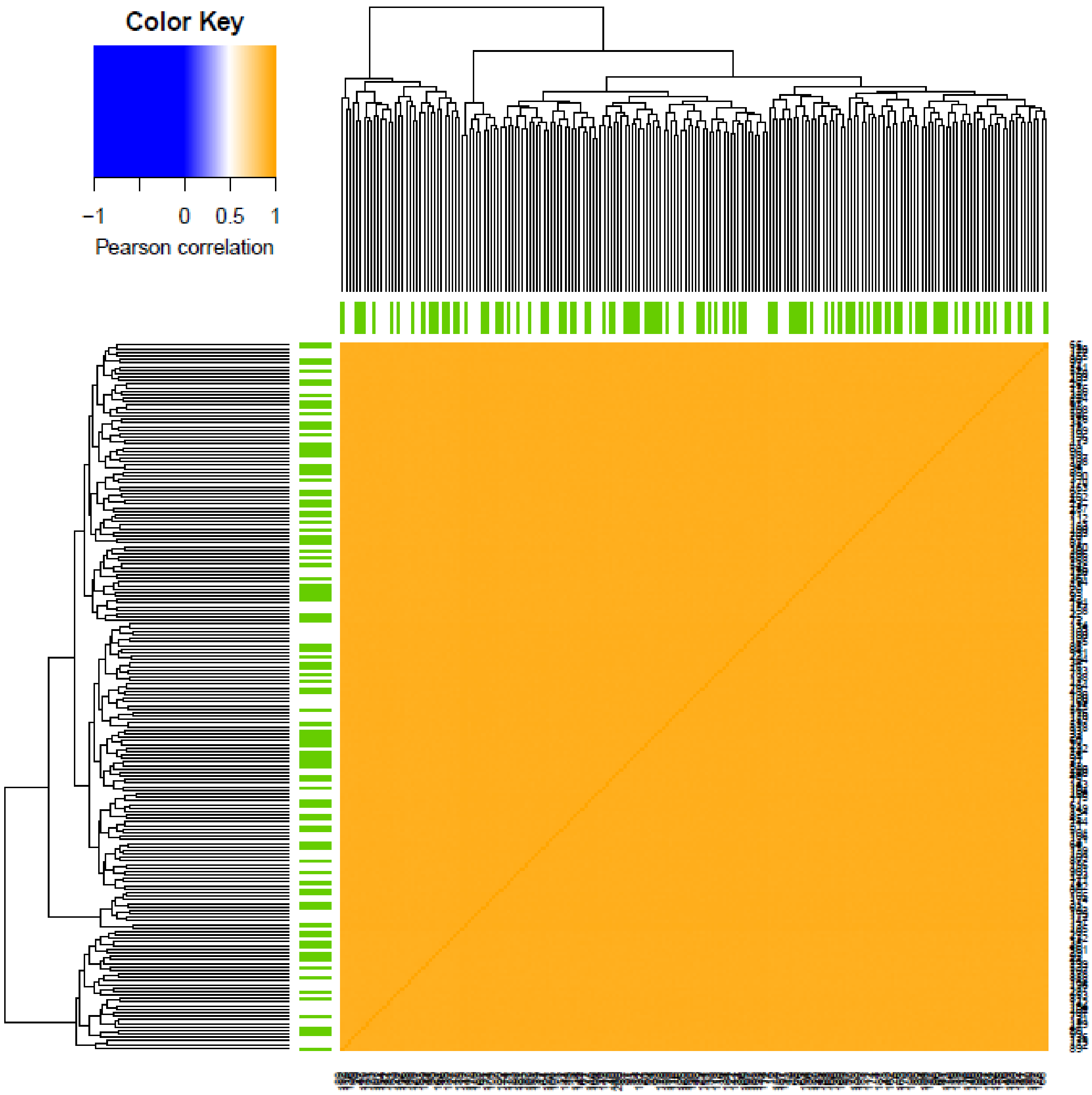

Figure S8: Similarity matrices of 100 simulated markers and 100 simulated false markers, with distance defined by the pairwise similarity statistic. Green sidebars indicate true markers.

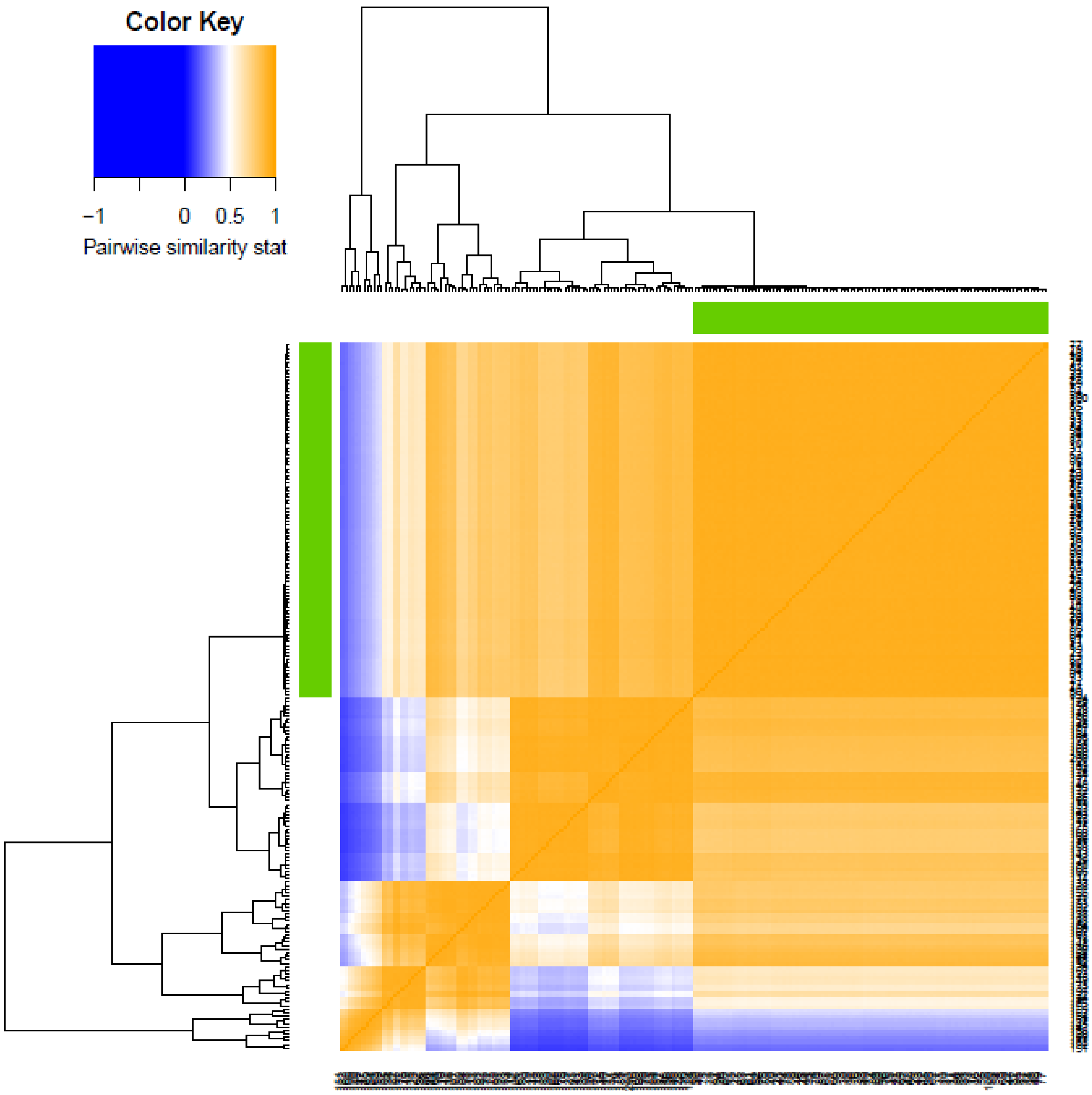

S2.7. Examples of pairwise similarity statistic

Figure S9 below displays the pairwise similarity statistic as applied to the TCGA bladder cancer data. For four cell types, we plot first a pair of selected marker genes and then a pair of one selected marker gene and one unselected candidate marker gene.

Figure S9: examples of the pairwise similarity statistic in the TCGA bladder cancer data. Each window plots two candidate markers against each other and reports their pairwise similarity statistic. Left side: pairs of selected marker genes. Right side: pairs of one selected and one rejected marker. Top row: B-cell genes. Second row: CD8 T cell genes. Third row: T cell genes. Fourth row: NK cell genes.

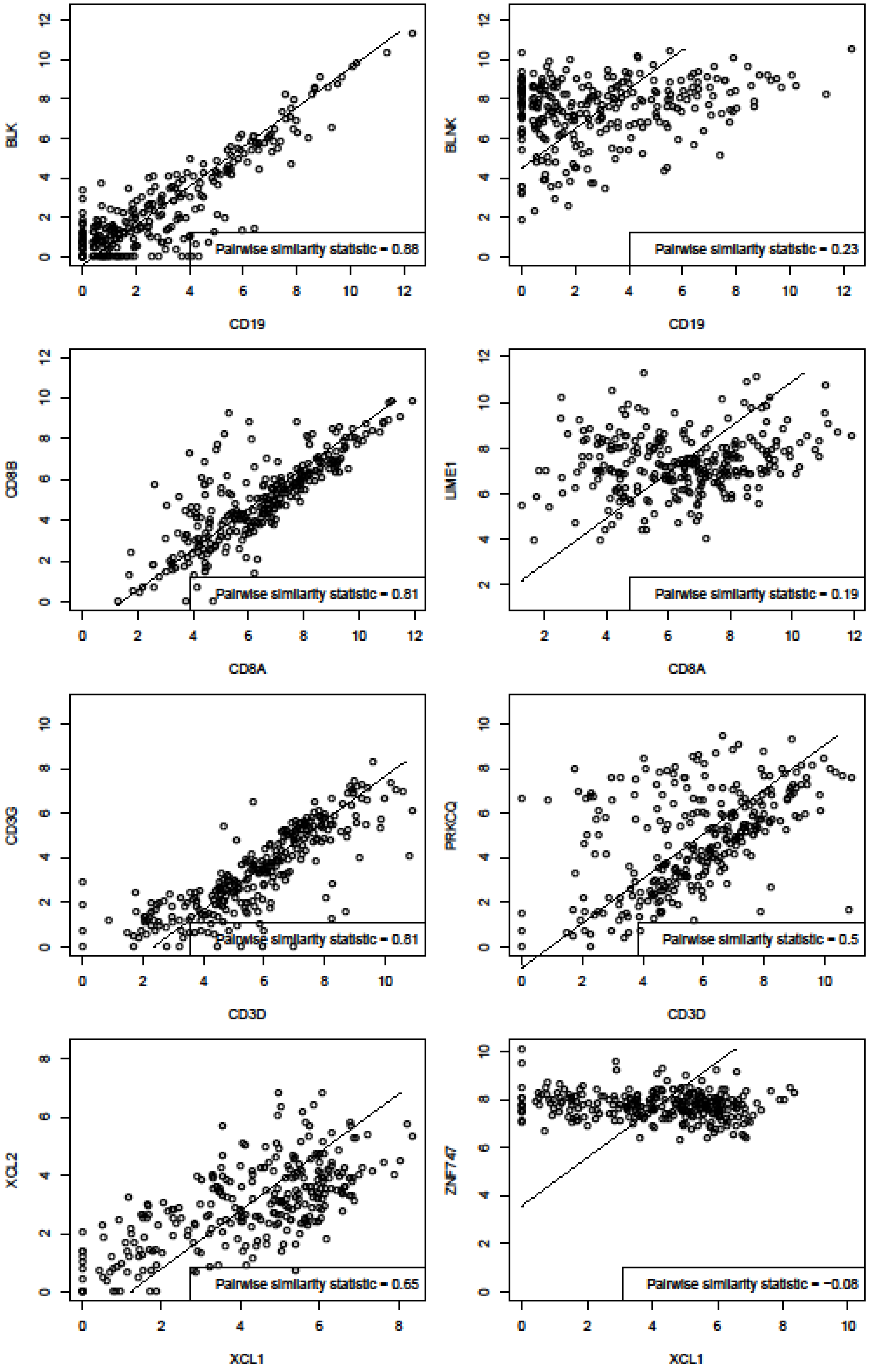

BLK
CD19
Pairwise similarity statistic = 0.88
BLNK
CD19
Pairwise similarity statistic = 0.23
CD8B
CD8A
Pairwise similarity statistic = 0.81
LIME1
CD8A
Pairwise similarity statistic = 0.19
CD3G
CD3D
Pairwise similarity statistic = 0.81
PRKCQ
CD3D
Pairwise similarity statistic = 0.5
XCL2
XCL1
Pairwise similarity statistic = 0.65
ZNF747
XCL1
Pairwise similarity statistic = −0.08

S3. Supplemental discussion

S3.1. Comparison to earlier gene expression cell type quantification methods

The TIL quantification method described here represents a substantial methodological departure from the standard deconvolution methodology, represented most prominently by Cibersort [2]. Deconvolution methods are powerful because they do not require genes that are entirely specific to one cell type, allowing them to exploit information from a wider gene set and in theory quantify closely related cell types.

However, deconvolution approaches depend strongly on the datasets used to train them, and they suffer when their training dataset is not representative of new data [3]. Both technical factors and the unusual biology of the tumor microenvironment have the potential to dissociate deconvolution training datasets from new datasets. Differences between gene expression platforms are likely the most damaging technical effect to deconvolution methods. If a new platform measures genes at different efficiencies than the platform used to generate a training dataset, it will introduce errors to the training dataset's cell type expression matrix and affect deconvolution results in hard to predict ways. The differential gene detection efficiencies caused by FFPE vs. frozen tissue and by different reagent batches pose similar problems.

Differential expression patterns of immune cells in the tumor microenvironment present a further risk to deconvolution methods, as immune cell expression in that setting could depart substantially from expression in the purified cells used to derive training datasets. Furthermore, it is well documented that tumors can express genes typically associated with immune cells [4]. Because deconvolution methods estimate all cell type prevalences jointly, a tumor that expresses even a few immune genes could distort the entire estimated cell type profile.

In contrast to deconvolution methods, our proposed method relies on a smaller set of highly cell type specific genes, and succeeds only to the extent that these genes are truly specific. However, our method is highly robust to platform differences and batch effects: if these effects are linear, they only change our cell type scores up to a constant. In other words, the ratio of two samples' cell type scores should be independent of platform under our method. A final benefit of the proposed method is that its motivating assumption – expression that is stable and specific to a single cell type – can be tested in each new dataset by assessing the marker genes' adherence to high correlation with unit slope. We detail a formal test for marker-like behavior in the Supplemental Methods.

The cell type quantification method of [5] is more similar to our method, with two key differences. First, they use the entire gene list from [6], while we use only the small subset of [6]'s gene list that we found to exhibit evidence for cell type specificity in TCGA. Although using more genes in theory leads to better technical stability, our results suggest that many of the genes identified in [6] perform poorly as cell type markers in the tumor microenvironment. Second, the method of [5] quantifies cells using single sample gene set enrichment analysis (ssGSEA), while we rely on a simple mean of each cell's marker genes. ssGSEA has the advantage of robustness to poor quality data and possibly to poor quality genes.

However, its output lacks a natural interpretation; in contrast, our method supports statements like "sample 1 has twice as much CD8 cell signal as sample 2."

We note that the proposed method is very similar in spirit to geNorm [7], the popular housekeeping/reference gene selection method. While we require cell type marker genes to have high correlation and a slope of 1, geNorm seeks reference genes whose ratios are constant across samples. In other words, we require the same co-expression pattern as geNorm, with the additional requirement that our genes are observed over a large enough range for correlation between them to be apparent. Thus our cell type markers can be thought of as reference genes for specific cell types.

References

1. Blankenstein T, Coulie PG, Gilboa E, Jaffee EM. The determinants of tumour immunogenicity. Nature Reviews Cancer. 2012;12(4):307-13.
2. Newman AM., et al. Robust enumeration of cell subsets from tissue expression profiles. Nature methods. 2015; 12(5):453-7.
3. Galon, Jérôme, et al. "The continuum of cancer immunosurveillance: prognostic, predictive, and mechanistic signatures." 2013;39(1):11-26.
4. He J, Hu Y, Hu M, Li B. Development of PD-1/PD-L1 pathway in tumor immune microenvironment and treatment for non-small cell lung cancer. Scientific reports. 2015;5.
5. Senbabaoglu Y, Winer AG, Gejman RS, Liu M, Luna A, Ostrovnaya I, Weinhold N, Lee W, Kaffenberger SD, Chen YB, Voss MH. The landscape of T cell infiltration in human cancer and its association with antigen presenting gene expression. bioRxiv. 2015;025908.
6. Bindea G, et al. Spatiotemporal dynamics of intratumoral immune cells reveal the immune landscape in human cancer. Immunity. 2013;39(4):782-95.
7. Vandesompele J, De Preter K, Pattyn F, Poppe B, Van Roy N, De Paepe A, Speleman F. Accurate normalization of real-time quantitative RT-PCR data by geometric averaging of multiple internal control genes. Genome biology. 2002;3(7):1-2.
