# Supplementary material for: Gene expression markers of Tumor Infiltrating Leukocytes
Source: J Immunother Cancer. 2017 Feb 21;5:18. doi: 10.1186/s40425-017-0215-8 (PMC5319024; doi:10.1186/s40425-017-0215-8)

Figure S10:
Tem: mean concordance across TCGA datasets

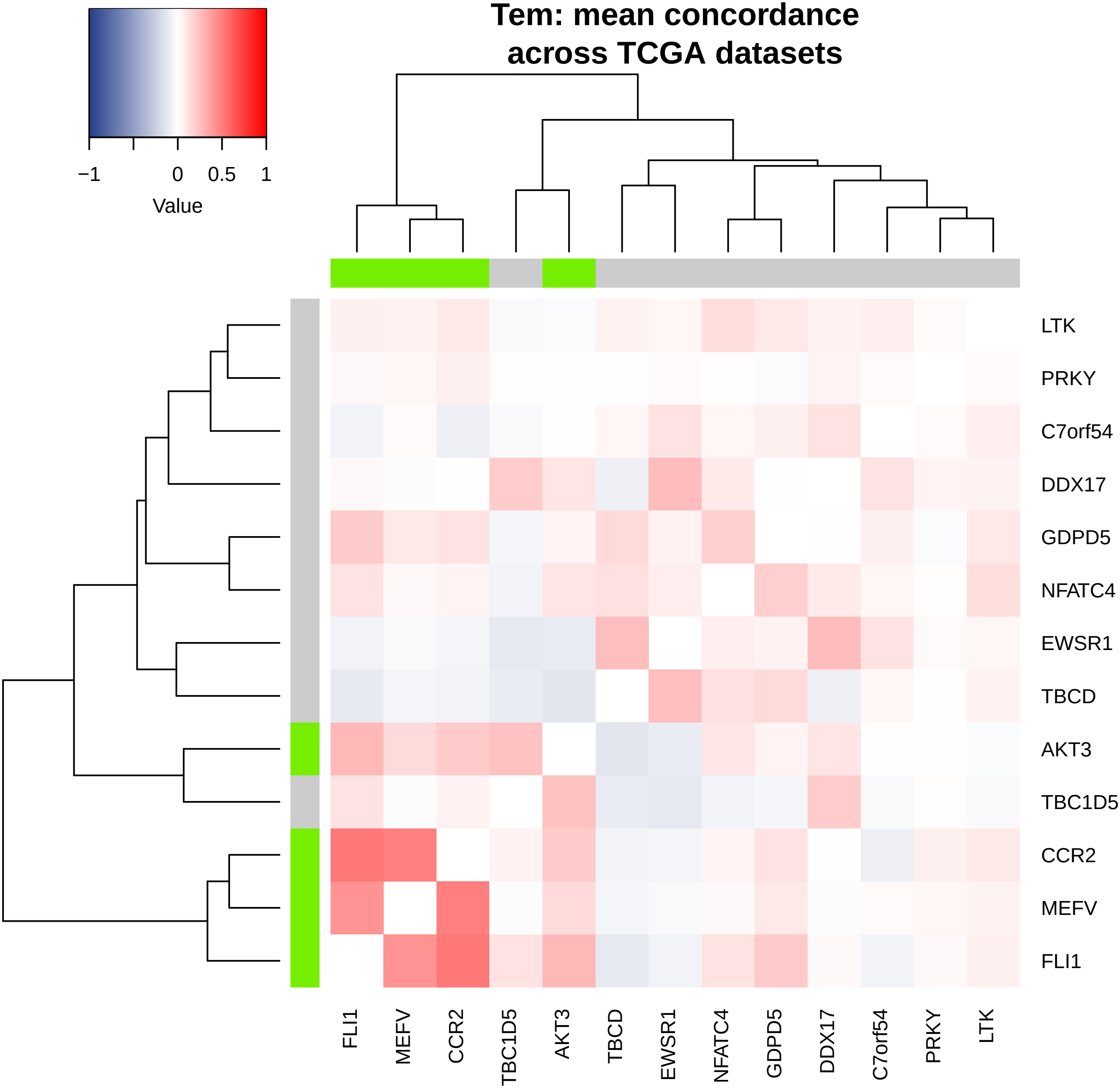

Figure S11:
T helper cells: mean concordance across TCGA datasets

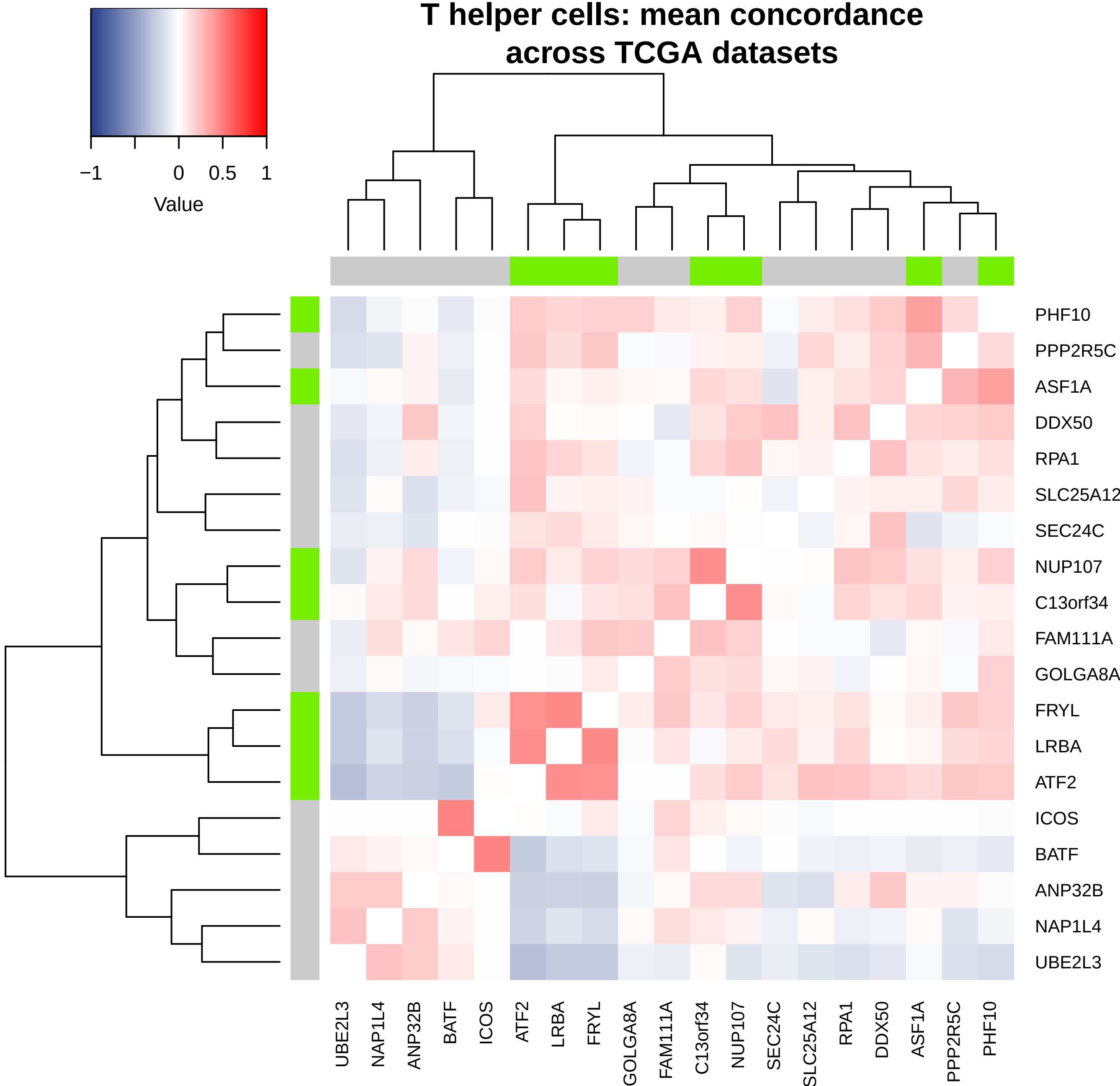

Figure S12:
Macrophages: mean concordance across TCGA datasets

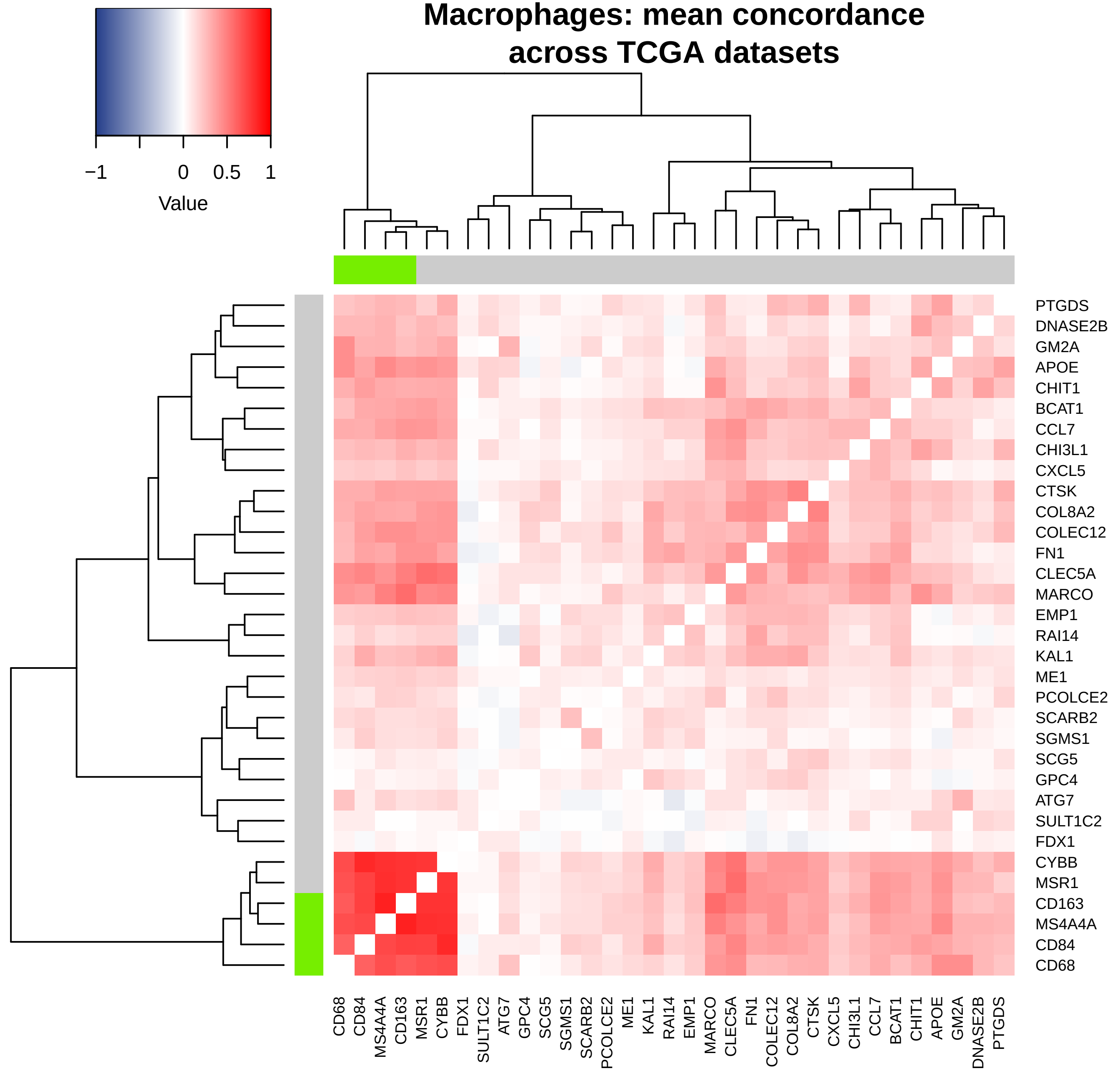

Figure S13: MDSC: mean concordance across TCGA datasets

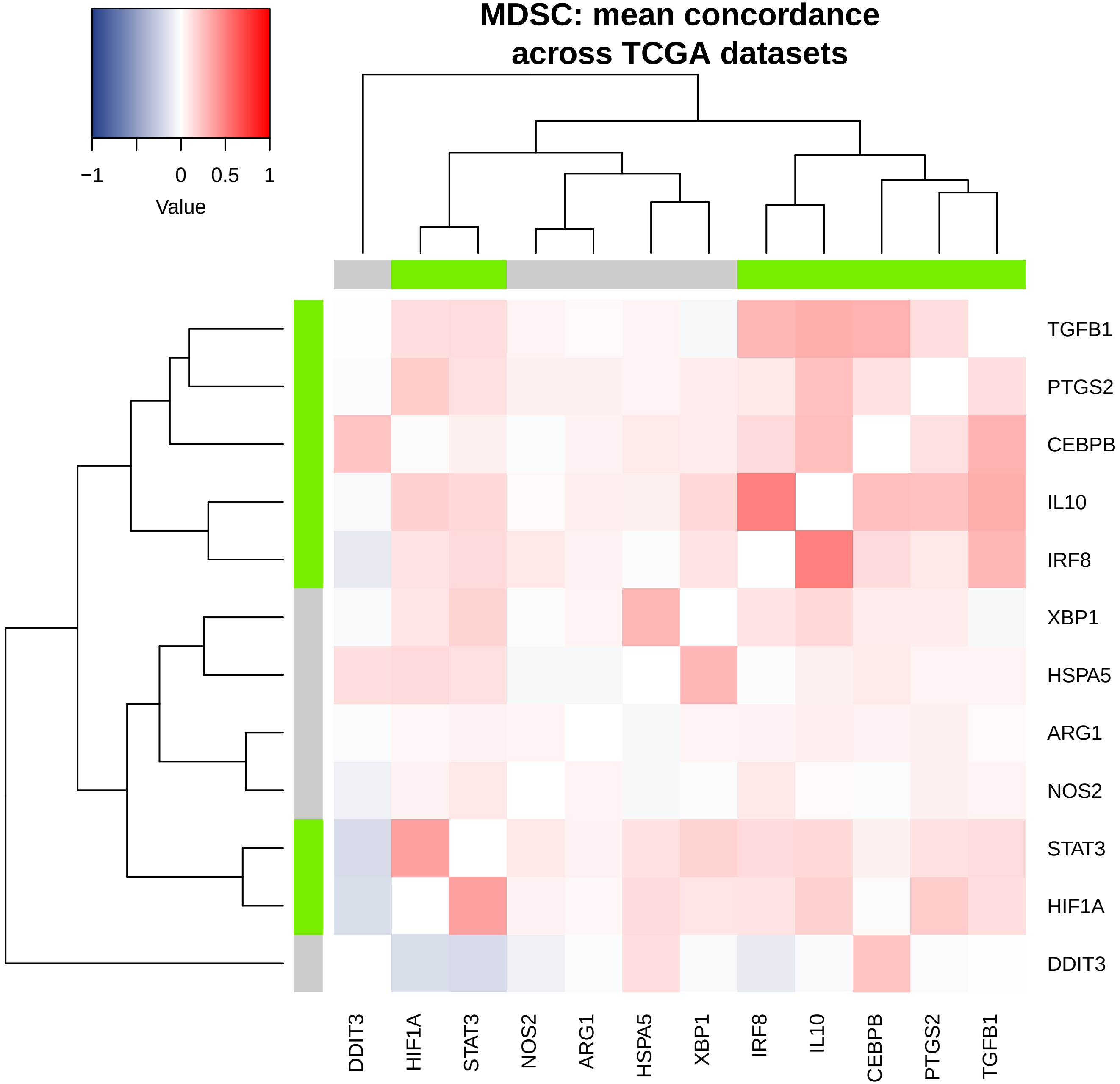

Figure S14:
Tcm: mean concordance across TCGA datasets

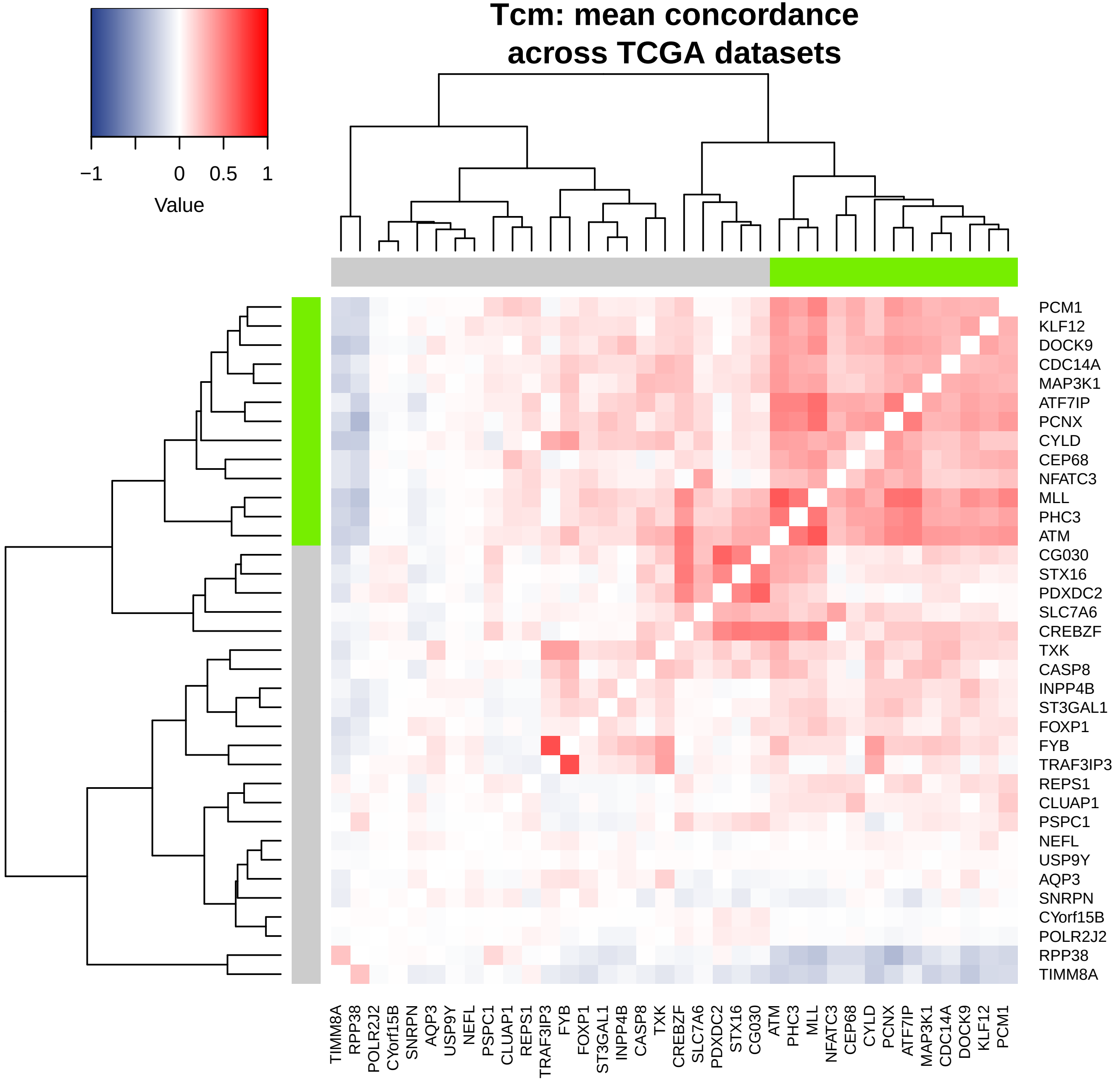

Figure S15: NK cells: mean concordance across TCGA datasets

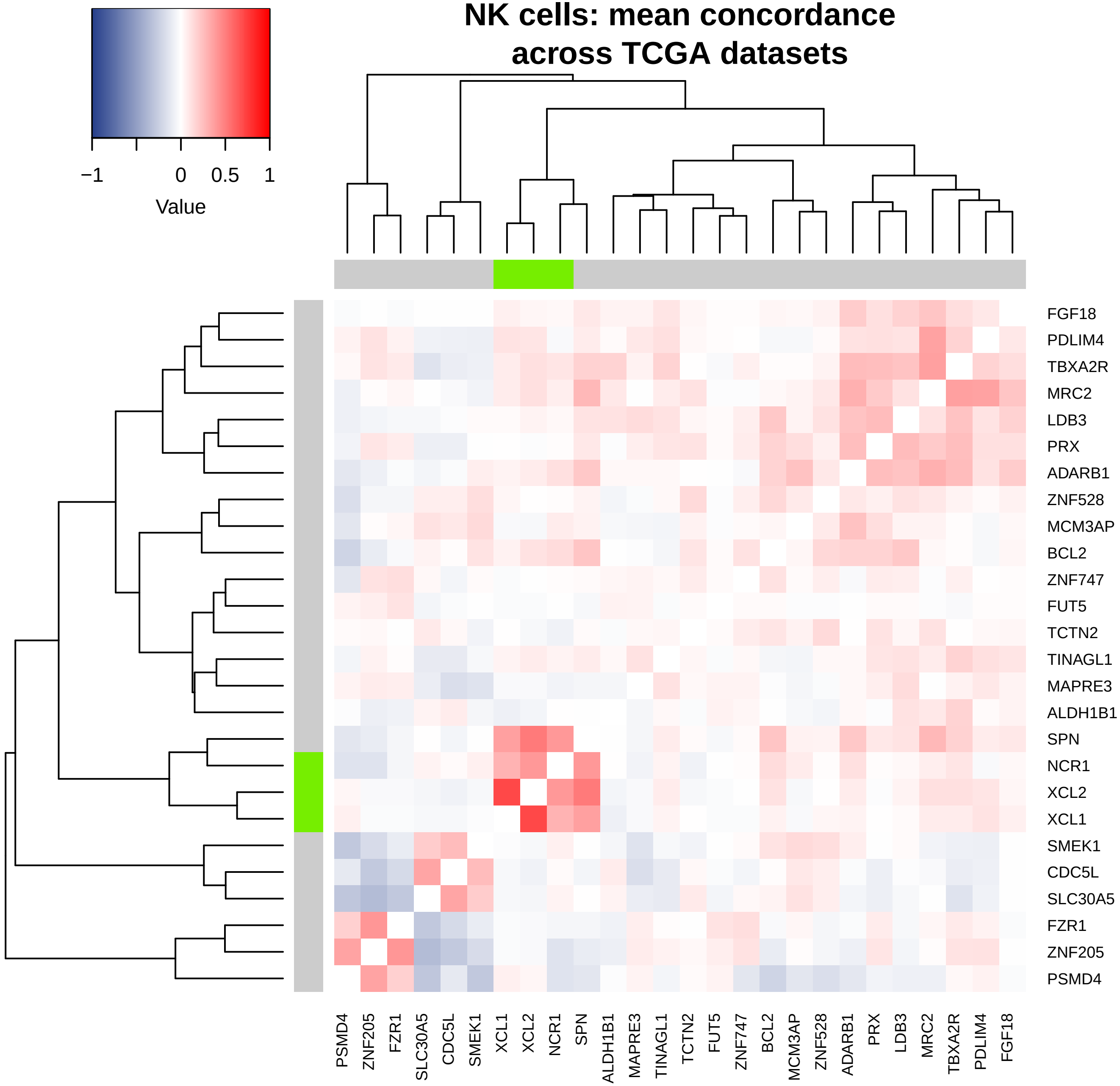

Figure S16:
Th2 cells: mean concordance across TCGA datasets

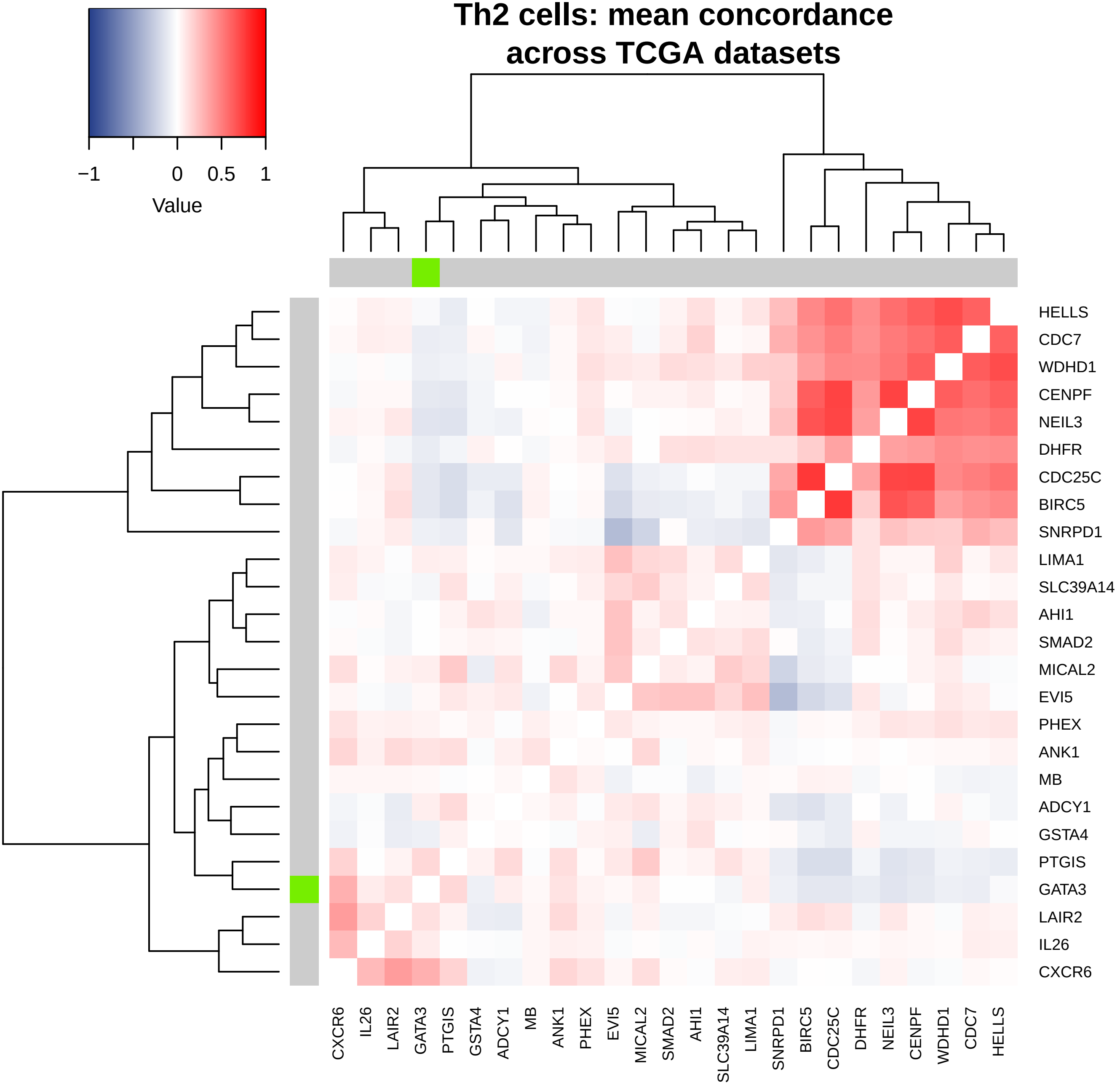

Figure S17: B−cells: mean concordance across TCGA datasets

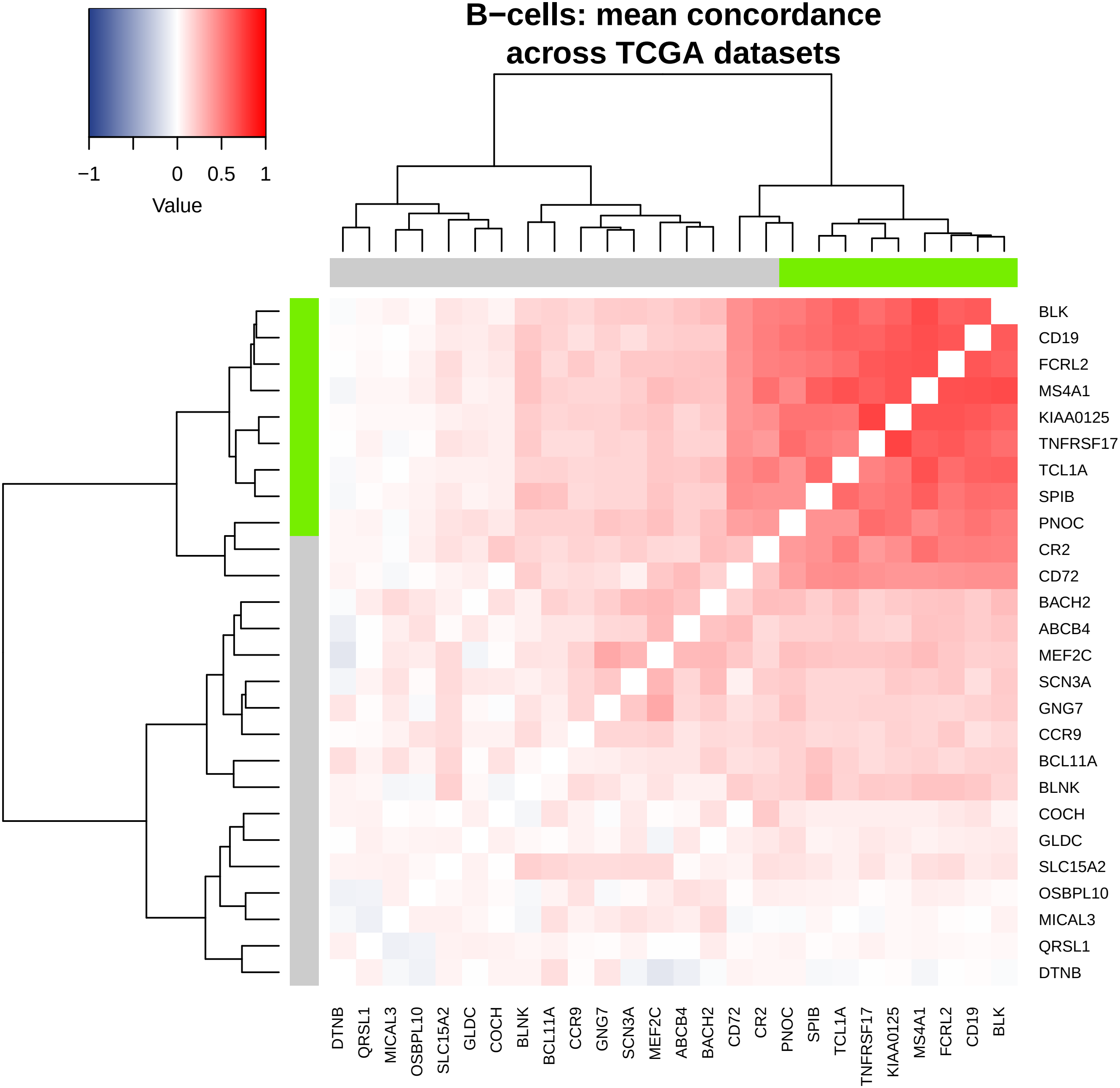

Figure S18:
Neutrophils: mean concordance across TCGA datasets

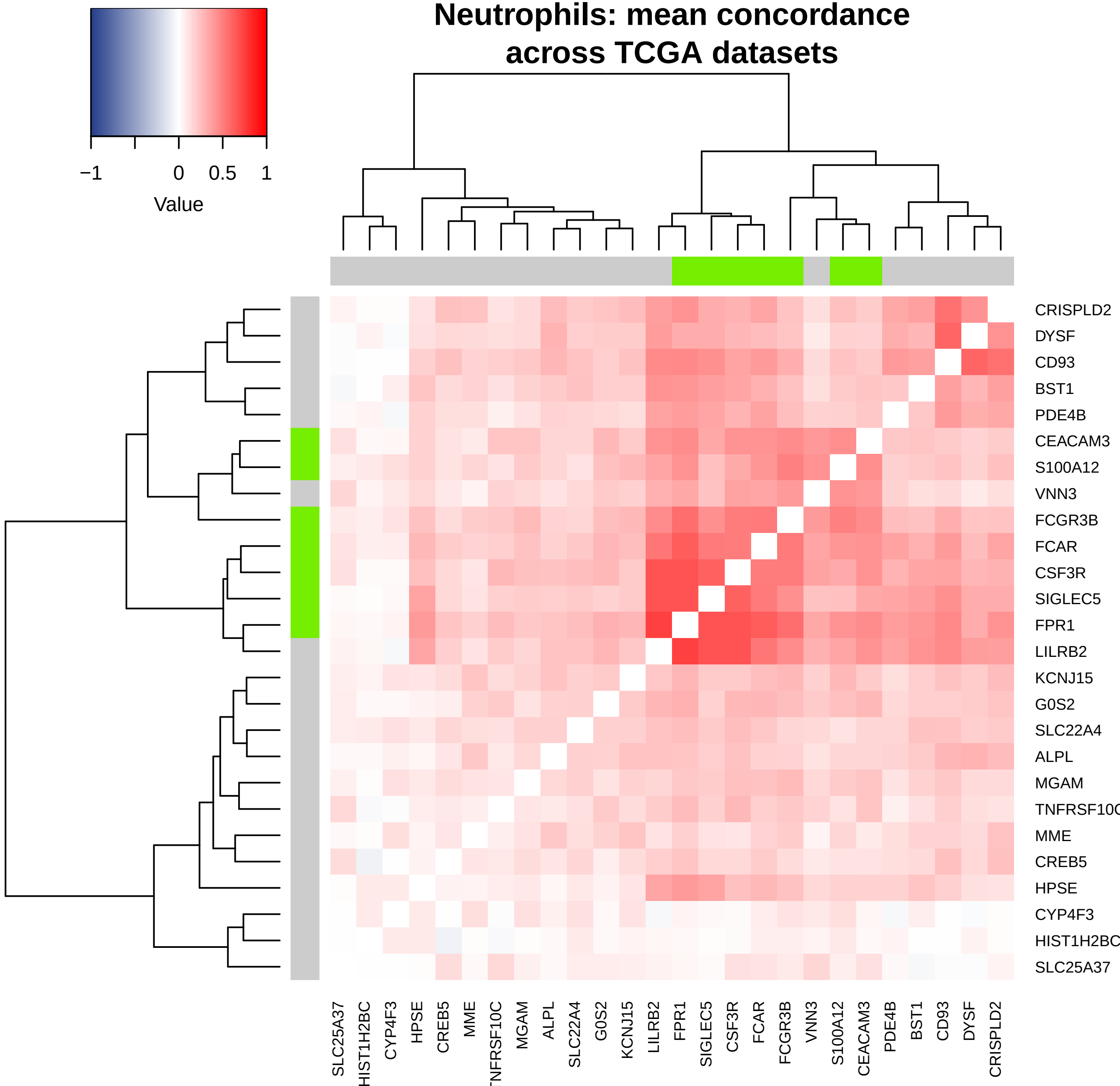

Figure S19:
Th1 cells: mean concordance across TCGA datasets

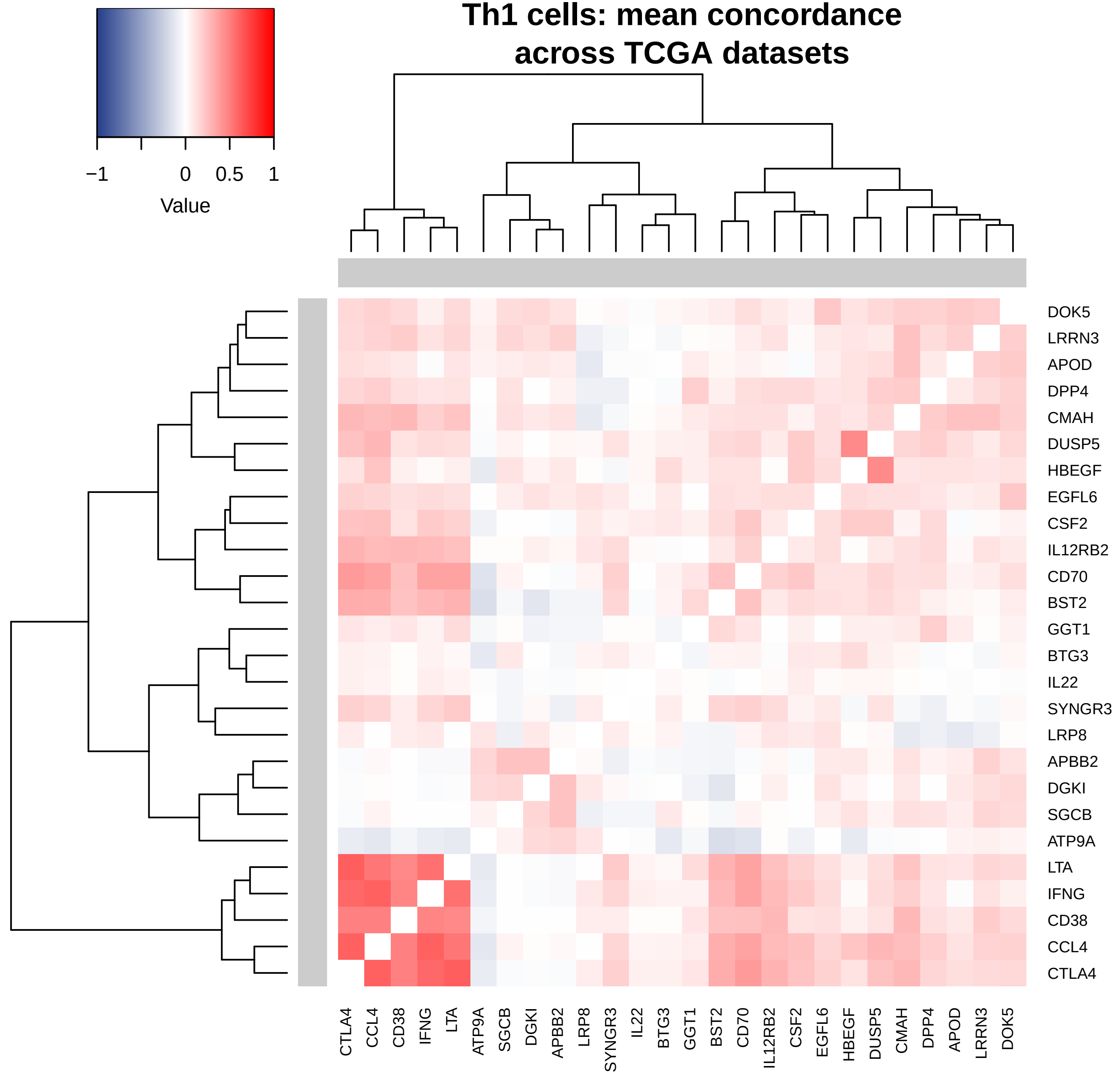

Figure S20:
Normal mucosa: mean concordance across TCGA datasets

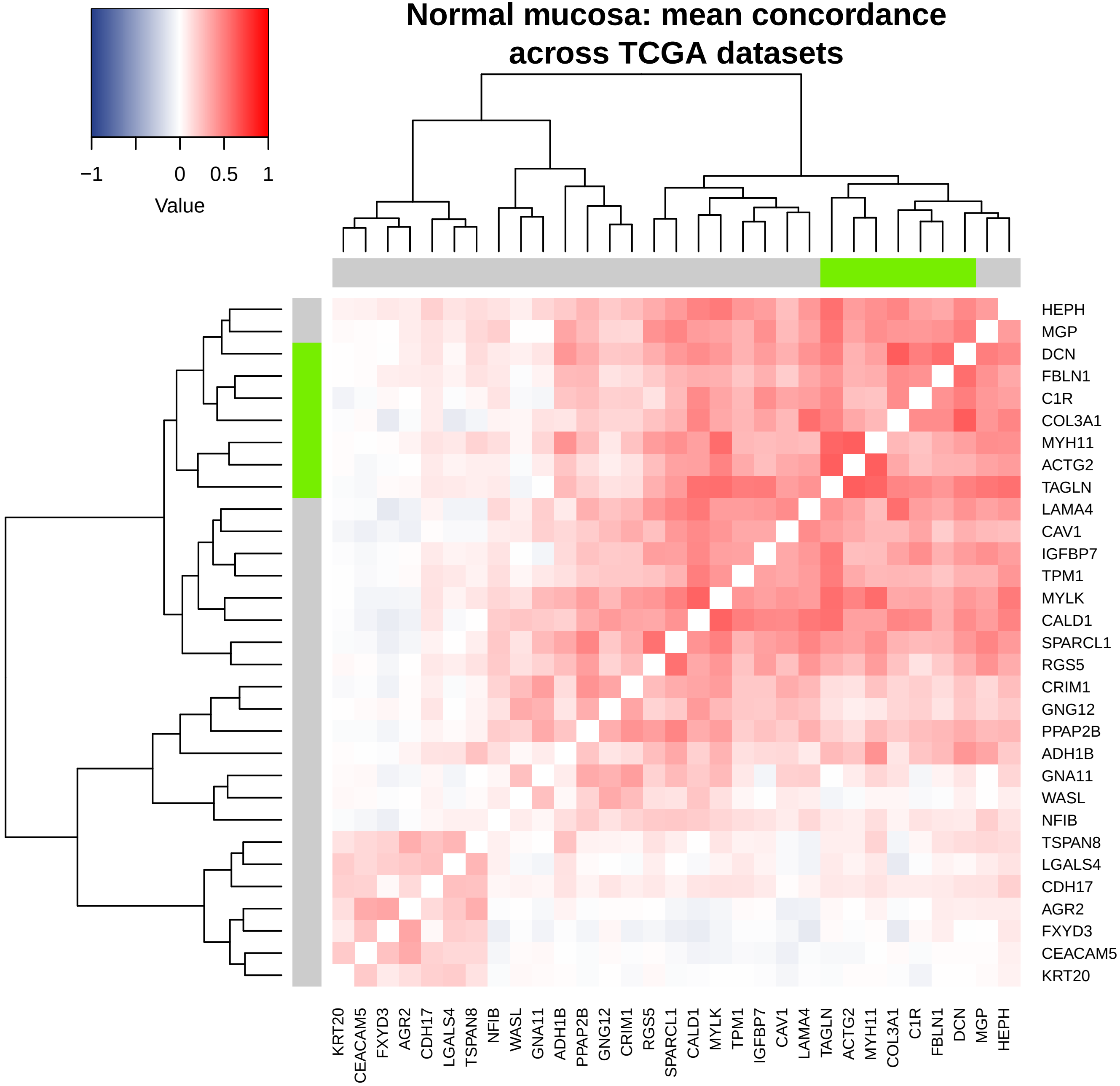

Figure S21: iDC: mean concordance across TCGA datasets

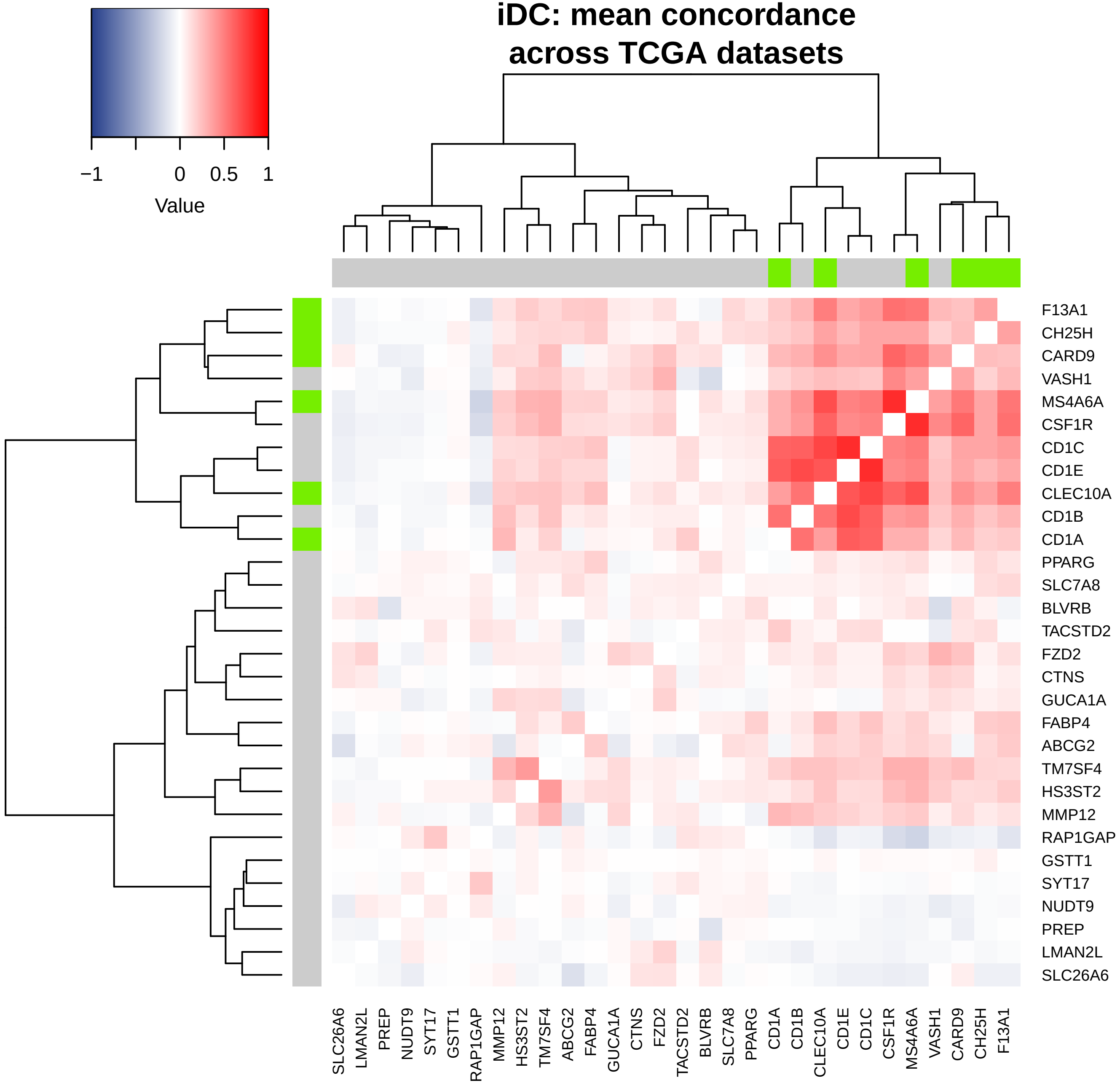

Figure S22:
aDC: mean concordance across TCGA datasets

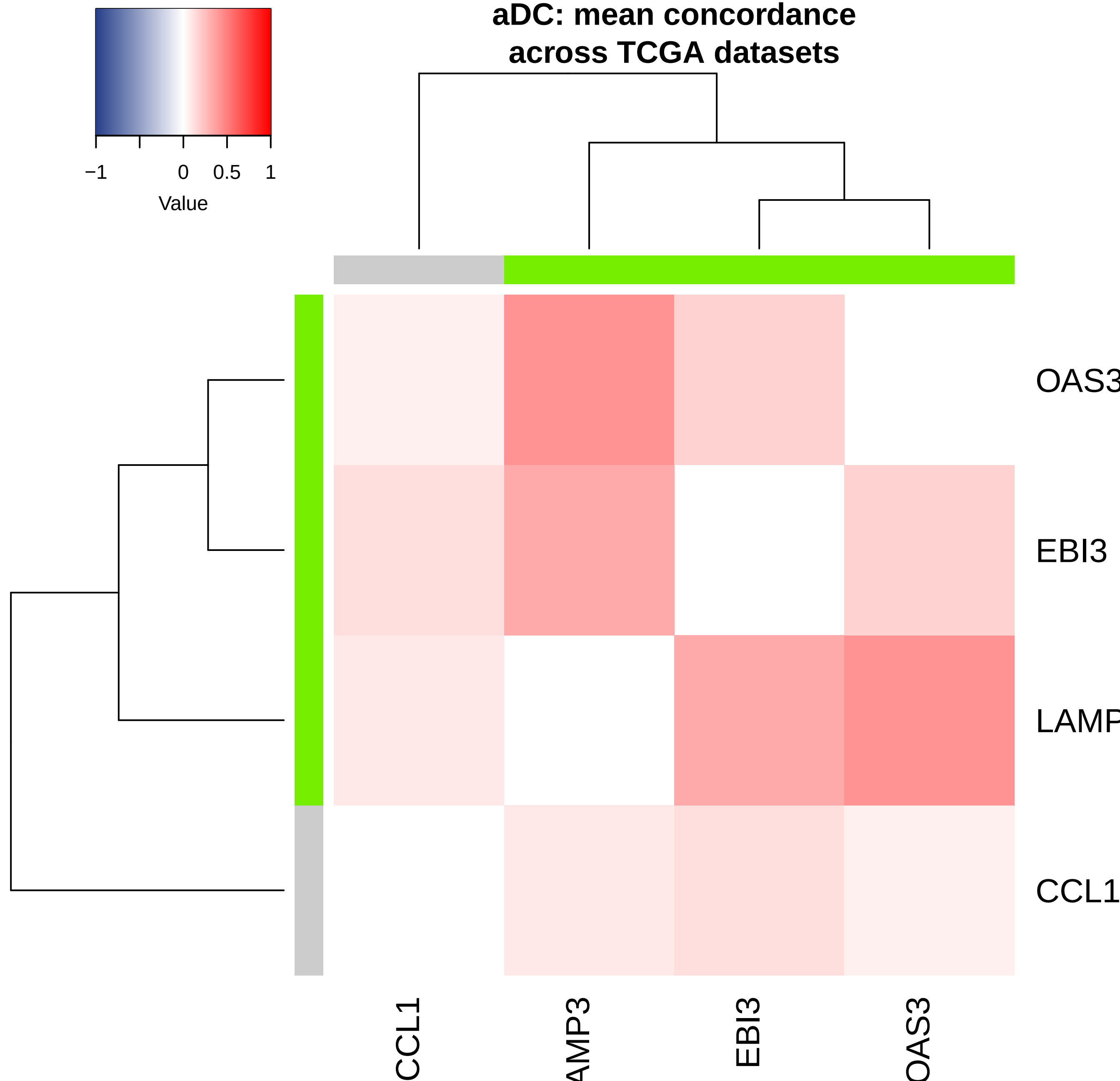

Figure S23:
DC: mean concordance across TCGA datasets

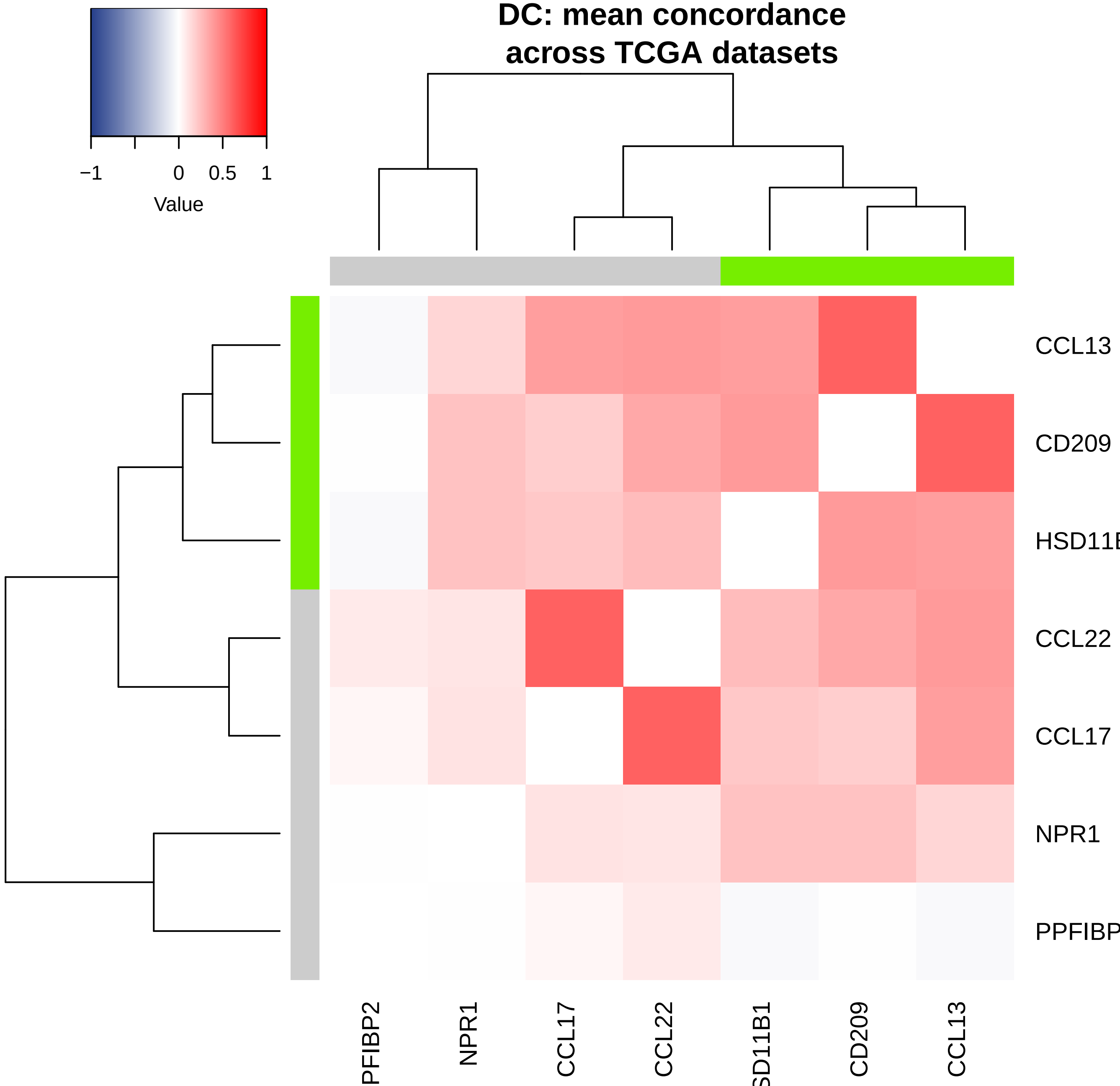

Figure S24:
Eosinophils: mean concordance across TCGA datasets

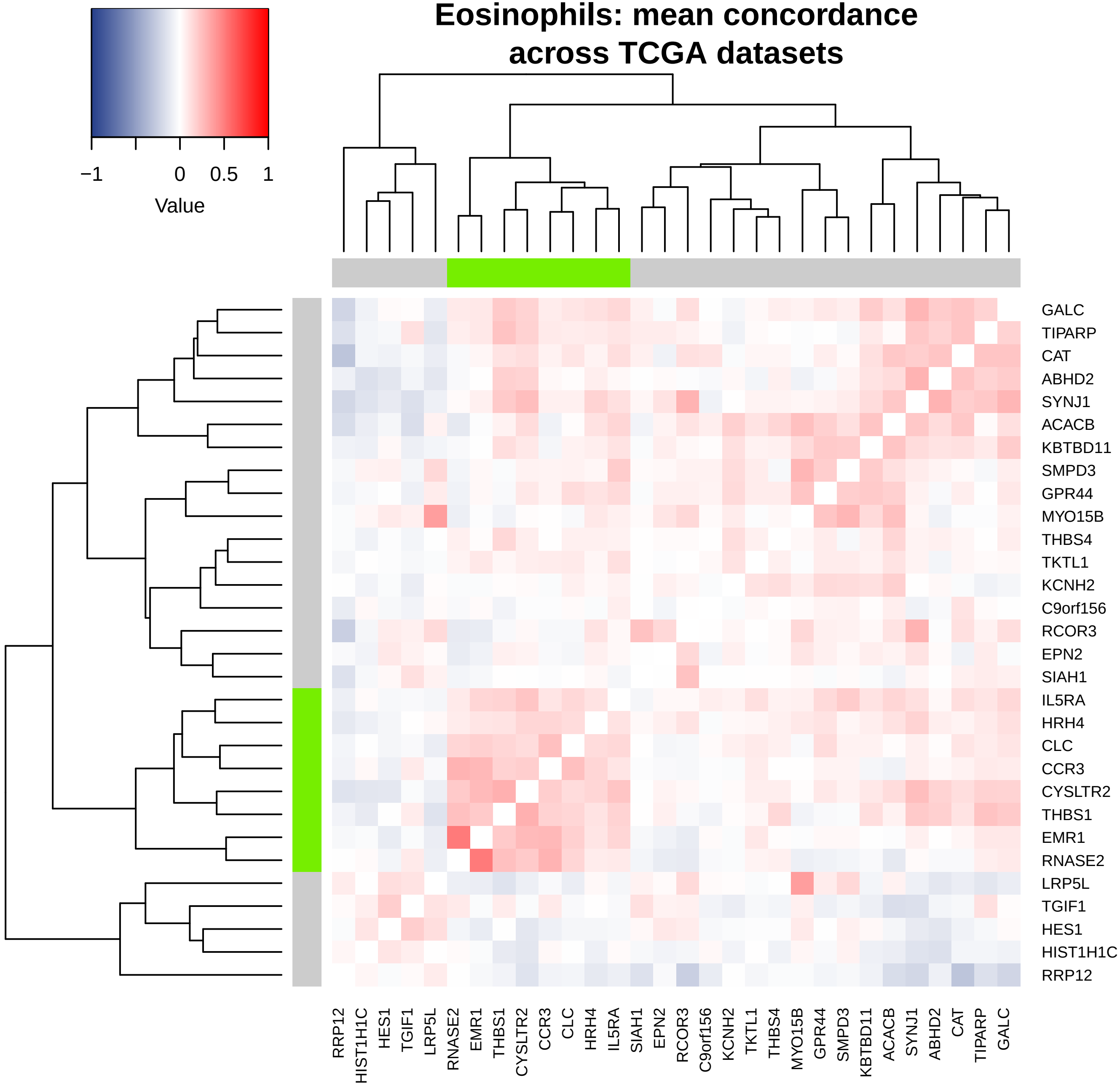

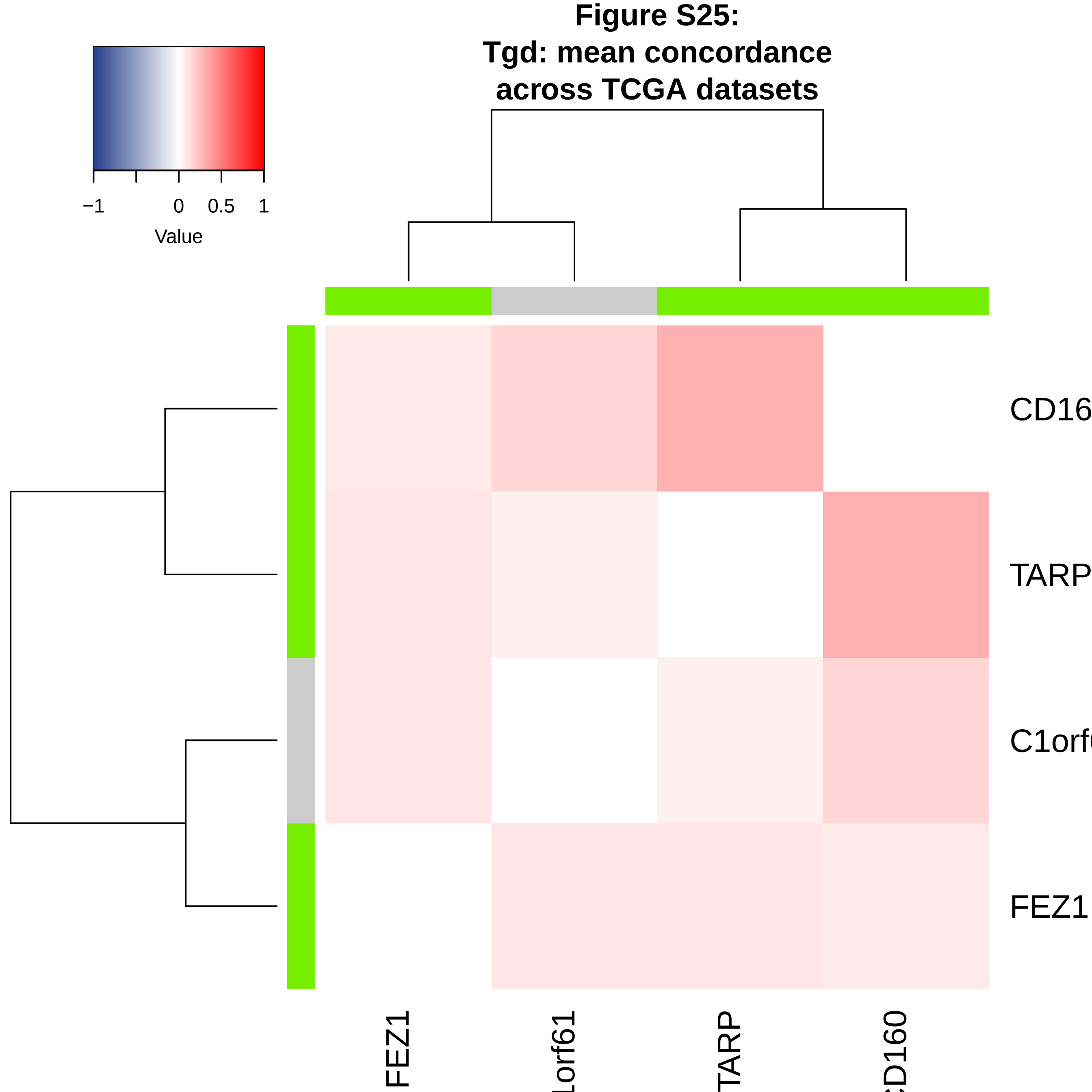

Figure S25:
Tgd: mean concordance
across TCGA datasets
−1
0
0.5
1
Value
CD16
TARP
C1orf
FEZ1
FEZ1
1orf61
TARP
CD160

Figure S26: T−cells: mean concordance across TCGA datasets

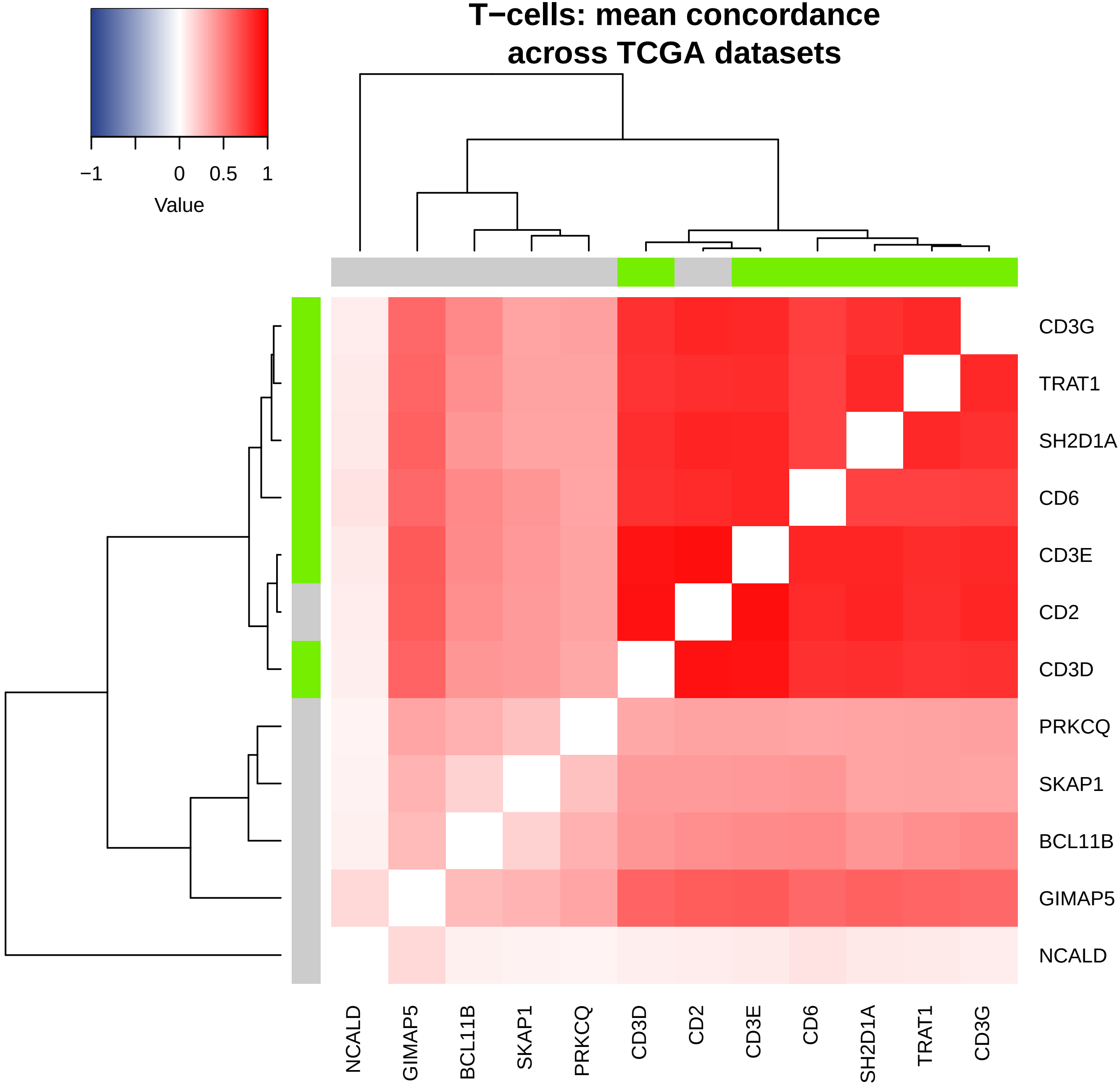

Figure S27:
Exhausted CD8: mean concordance across TCGA datasets

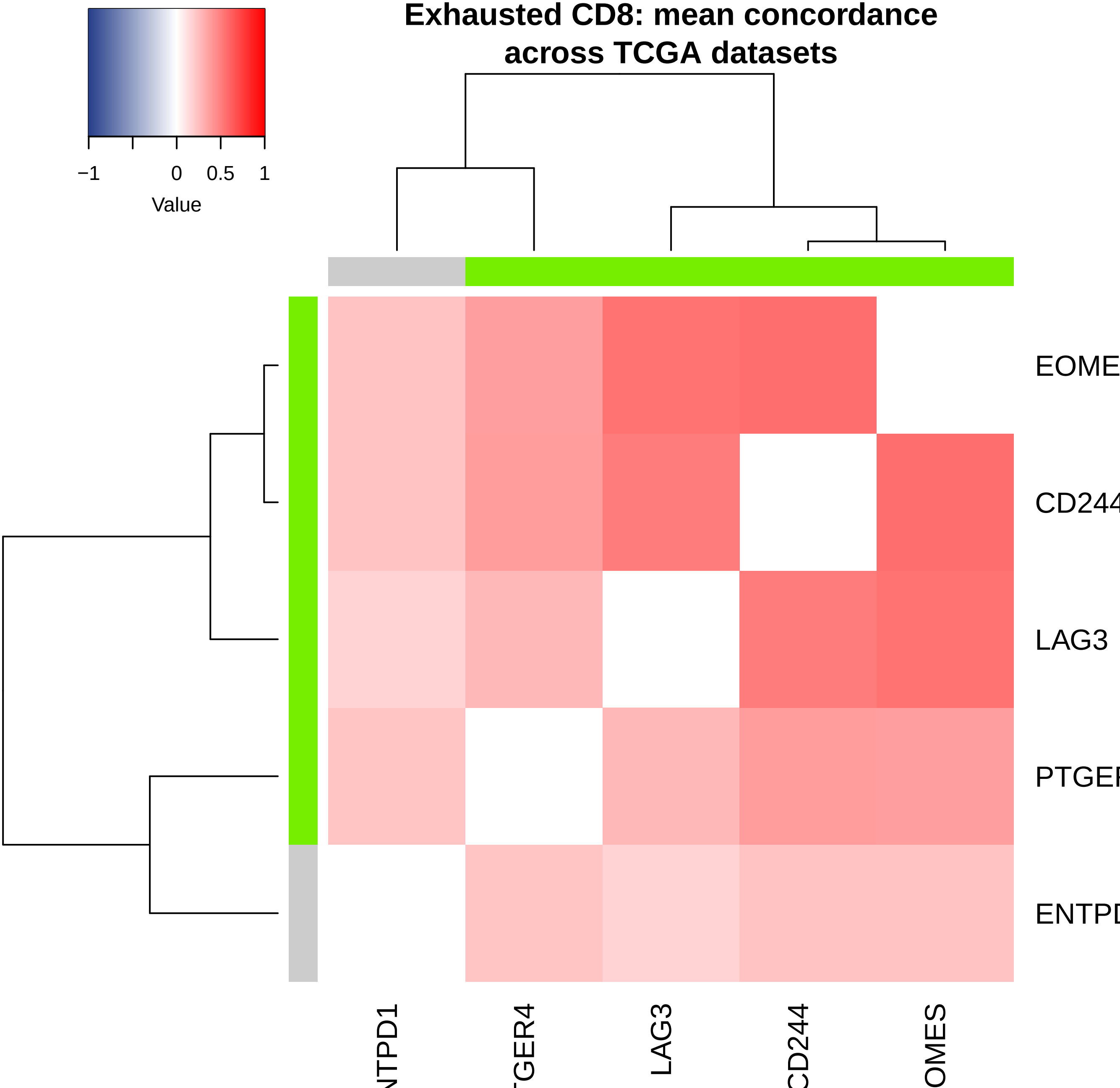

Figure S28: CD8 T cells: mean concordance across TCGA datasets

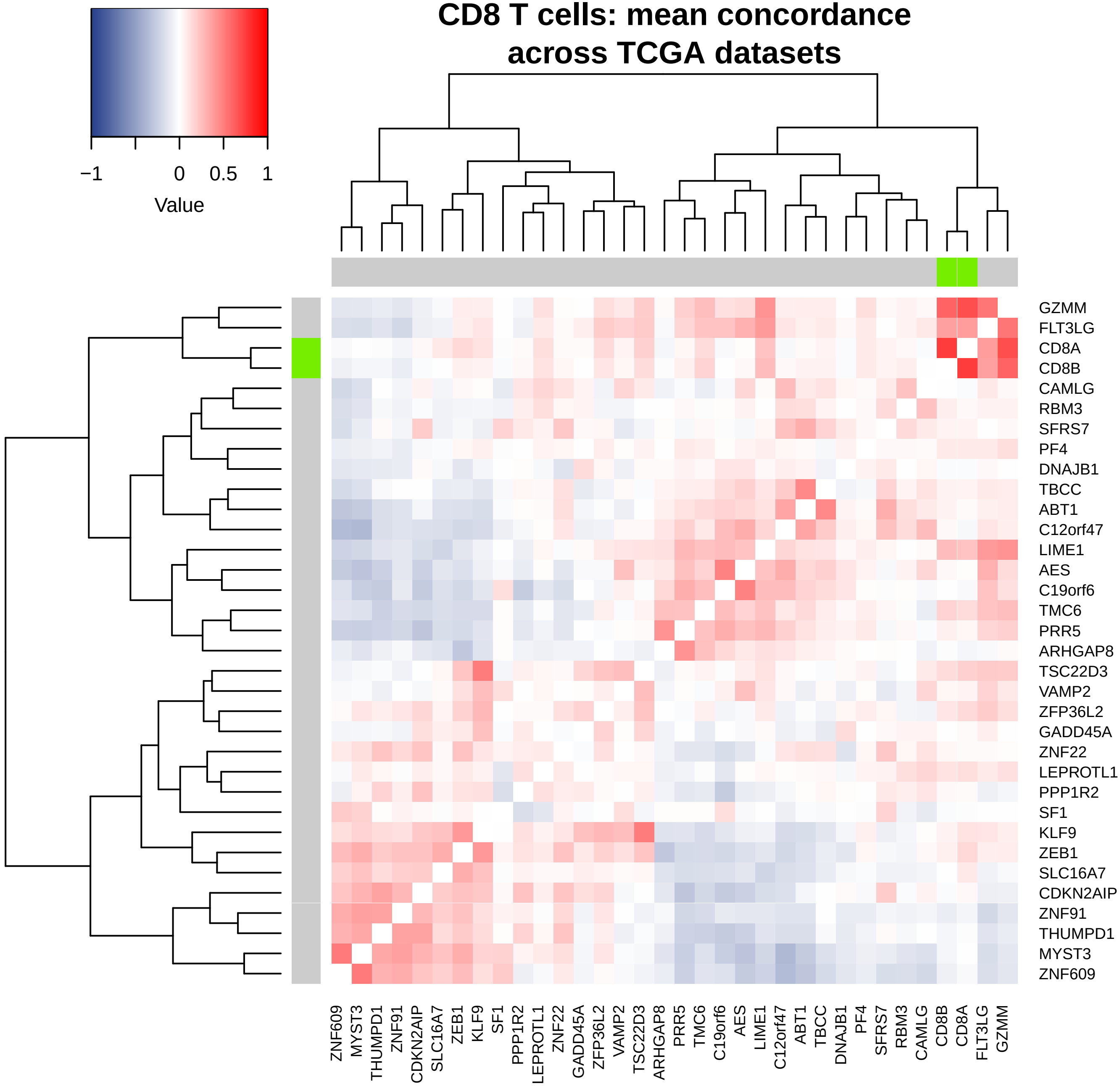

Figure S29: Mast cells: mean concordance across TCGA datasets

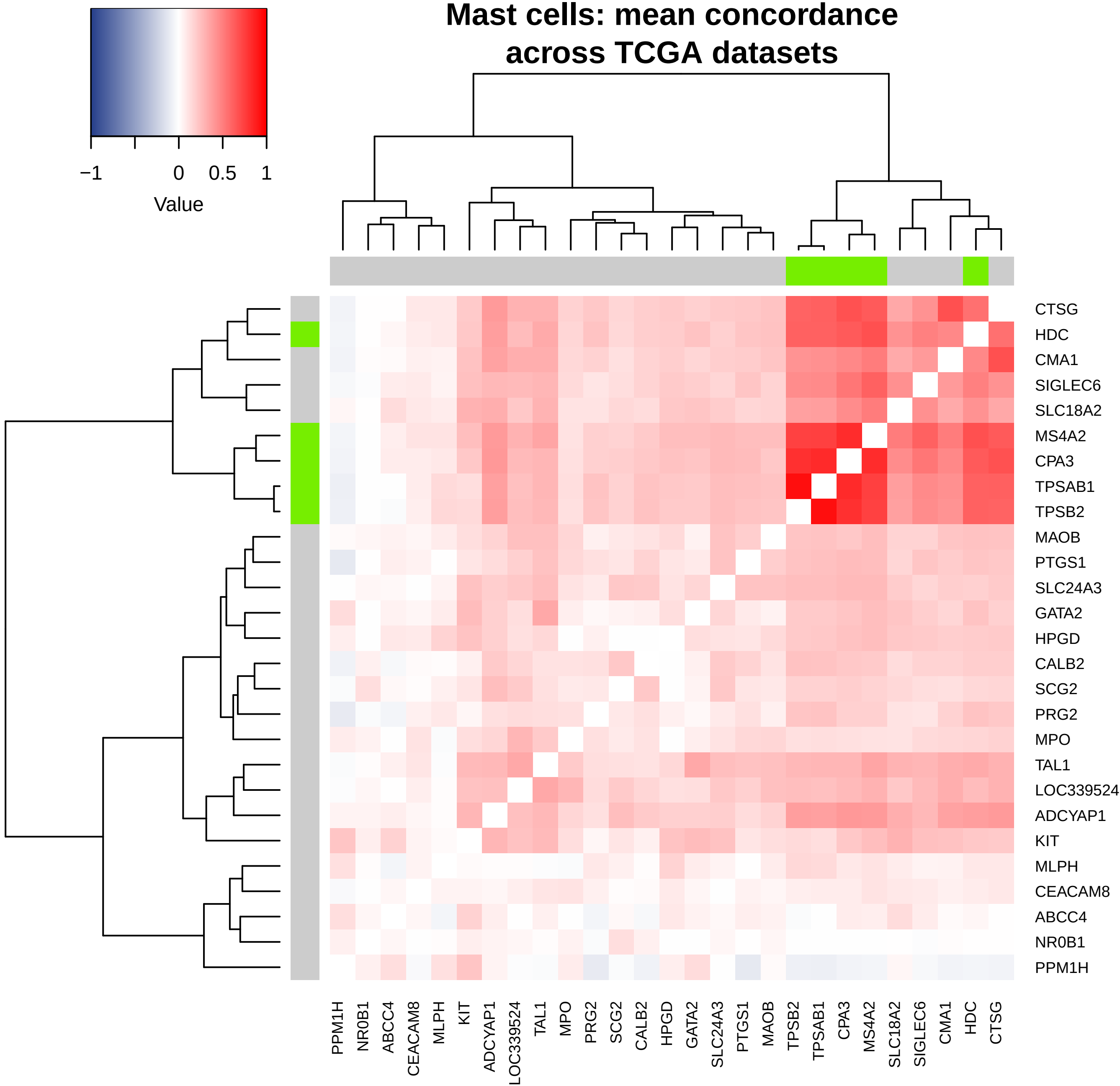

Figure S30: Treg: mean concordance across TCGA datasets

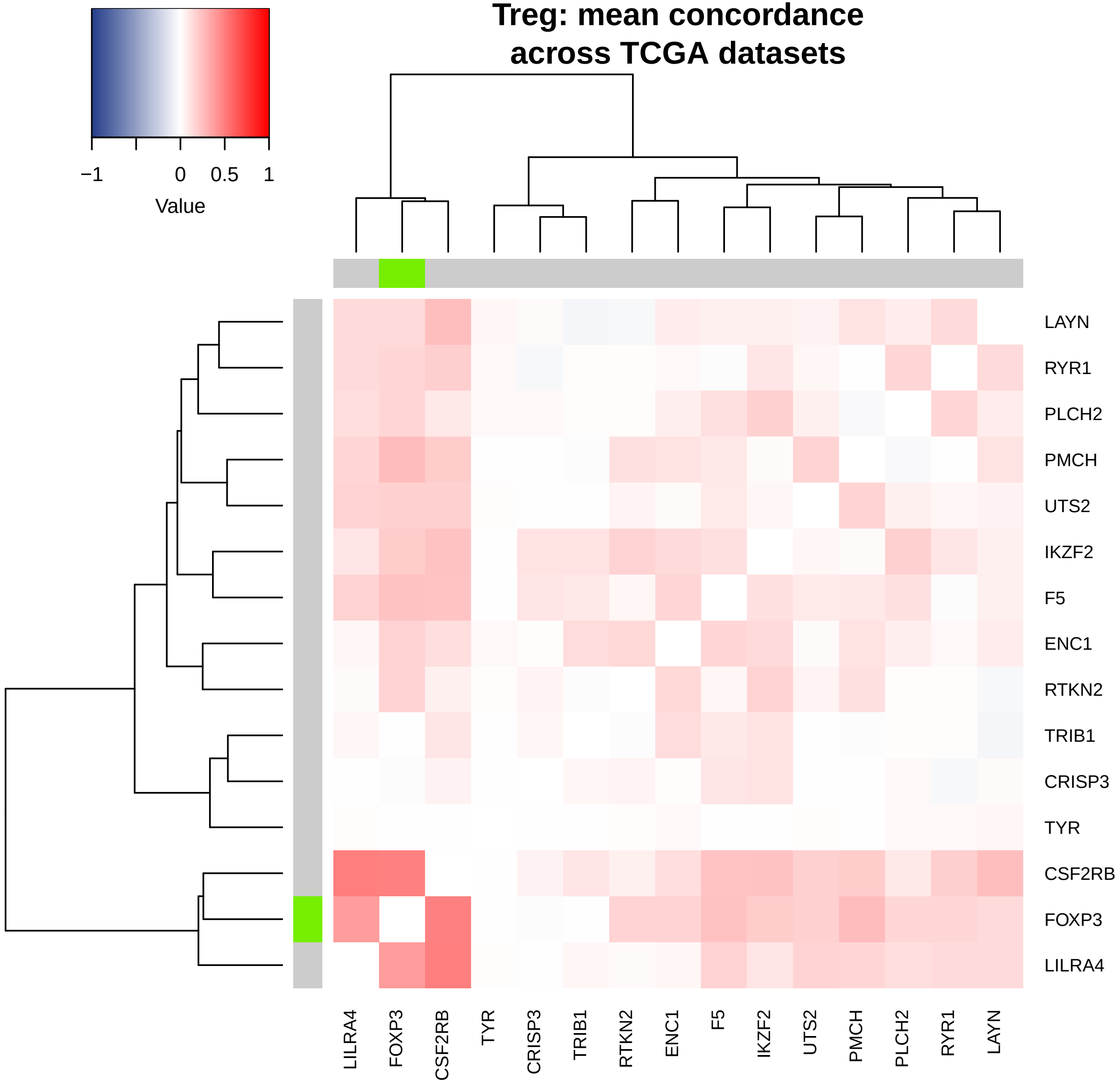

Figure S31:
Cytotoxic cells: mean concordance across TCGA datasets

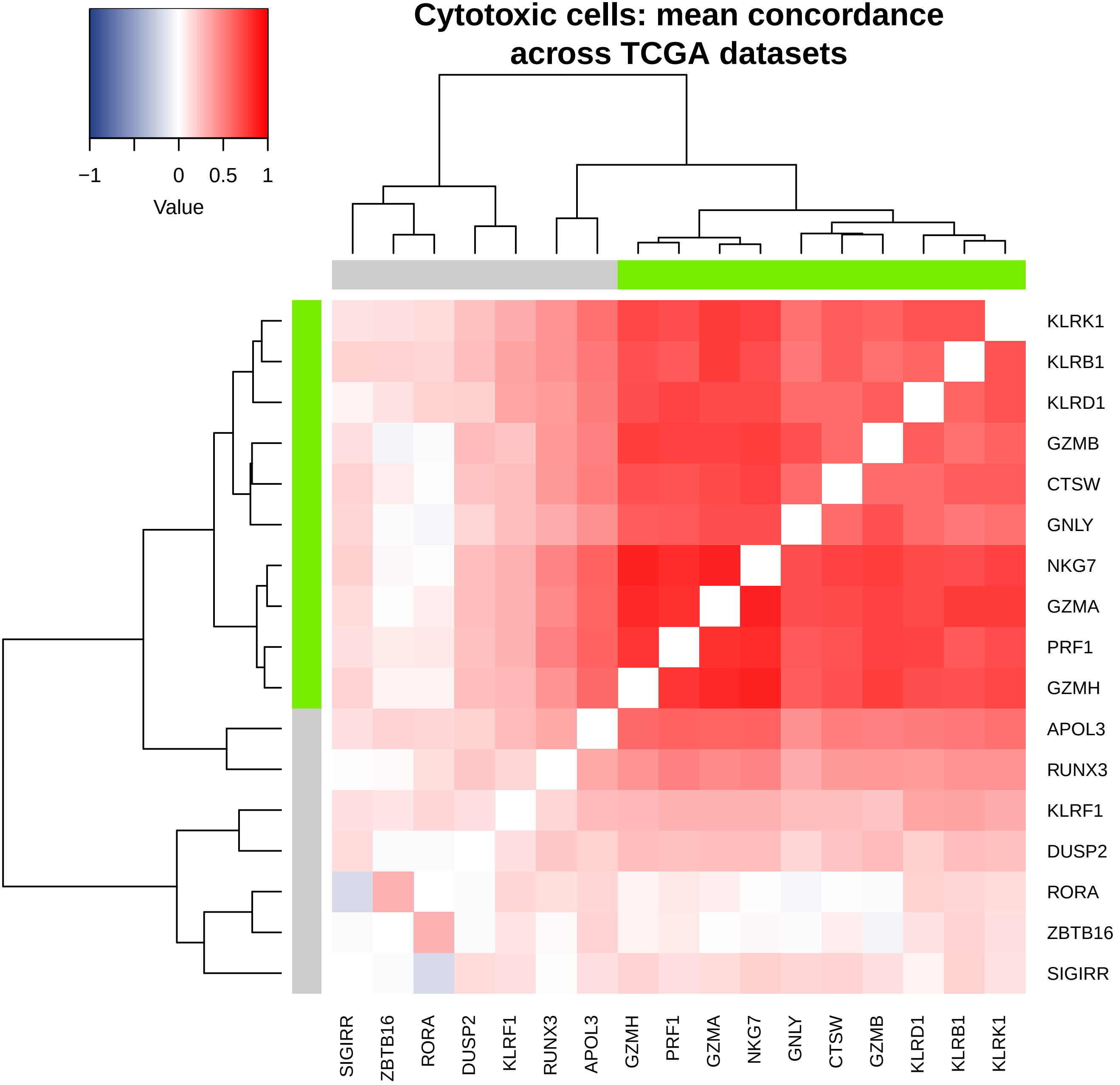

Figure S32:
TFH: mean concordance across TCGA datasets

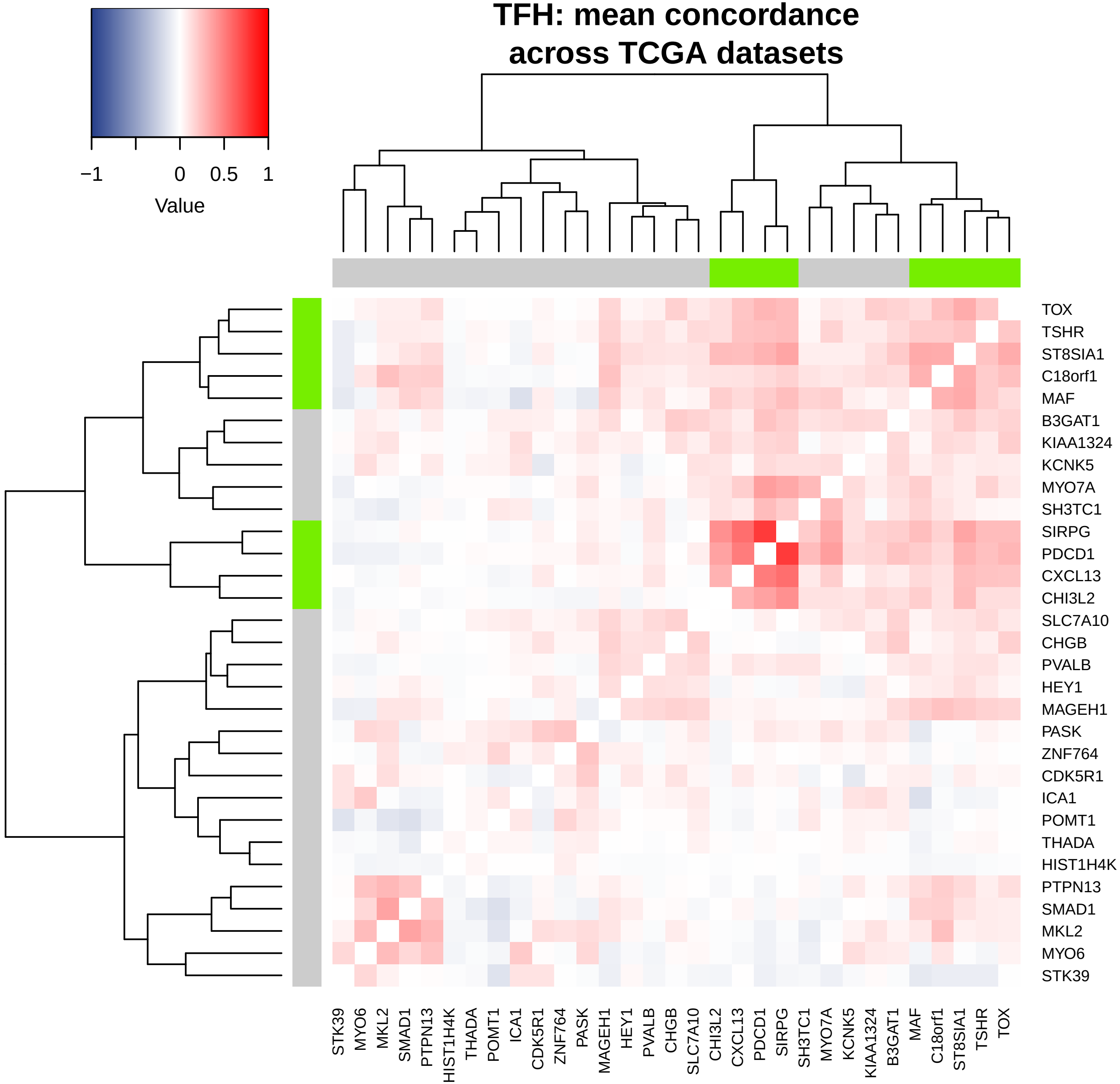

Figure S33:
NK CD56bright cells: mean concordance across TCGA datasets

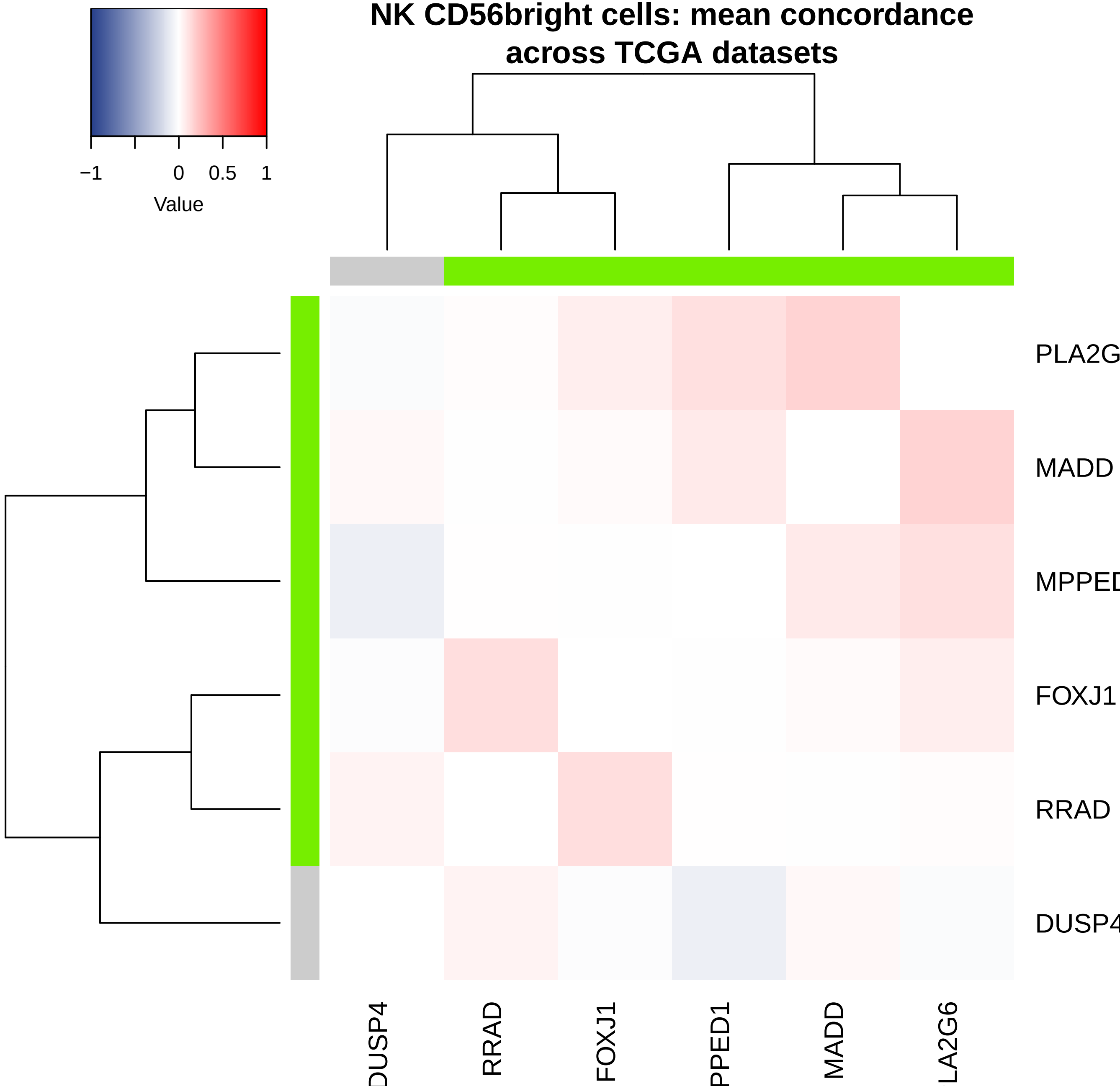

Figure S34:
SW480 cancer cells: mean concordance across TCGA datasets

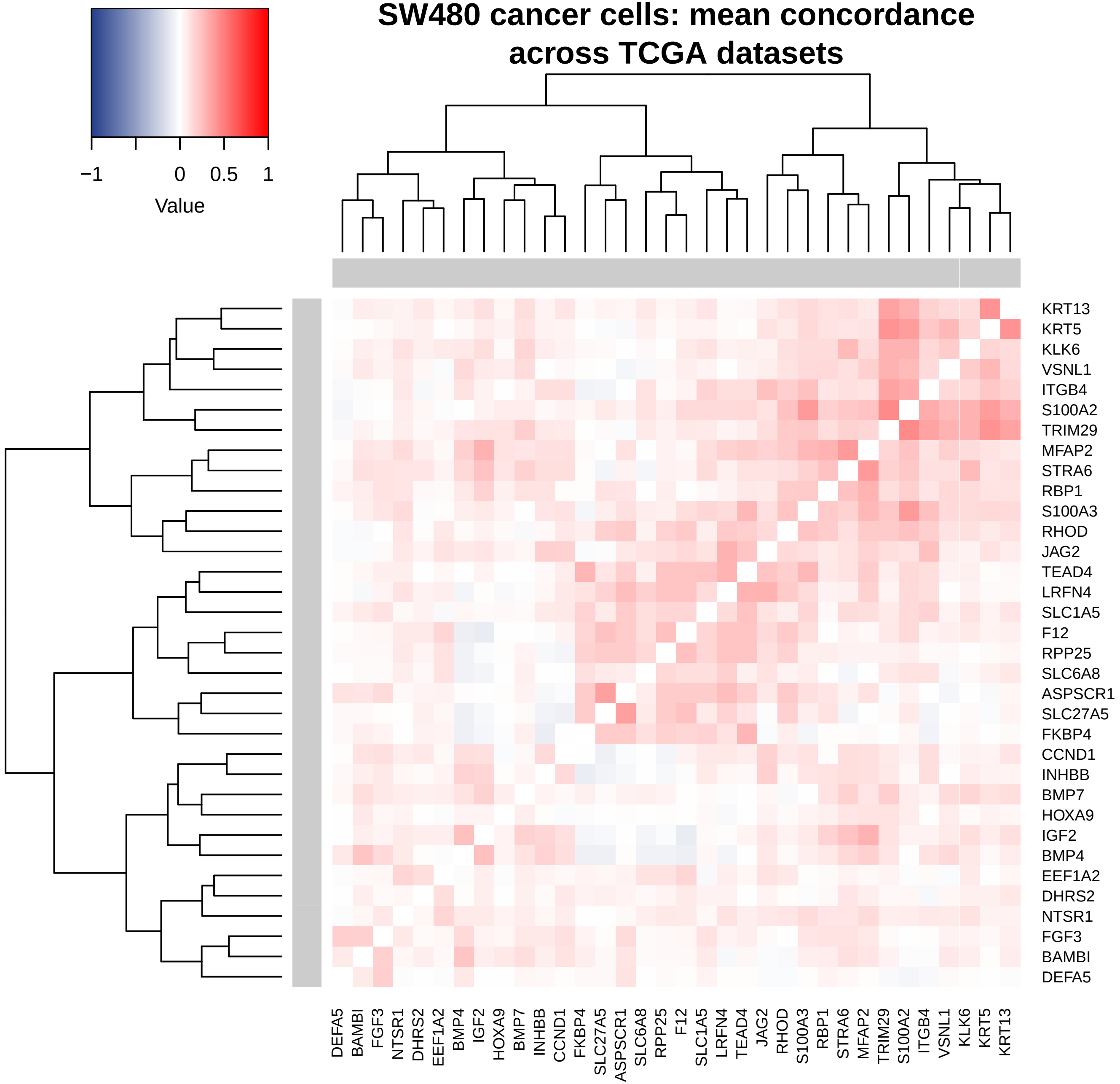

Figure S35:
NK CD56dim cells: mean concordance across TCGA datasets

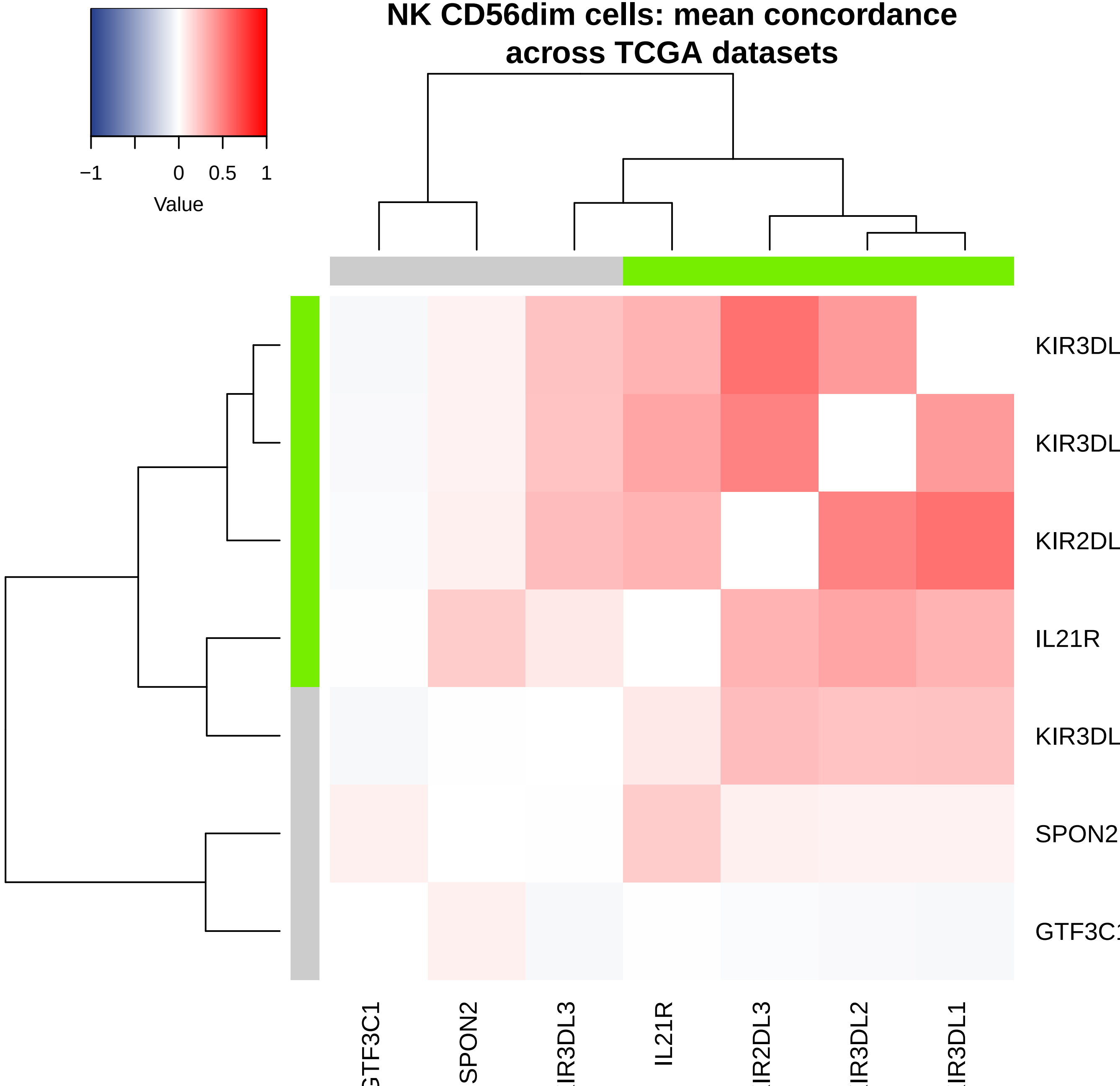

Figure S36:
Th17 cells: mean concordance across TCGA datasets

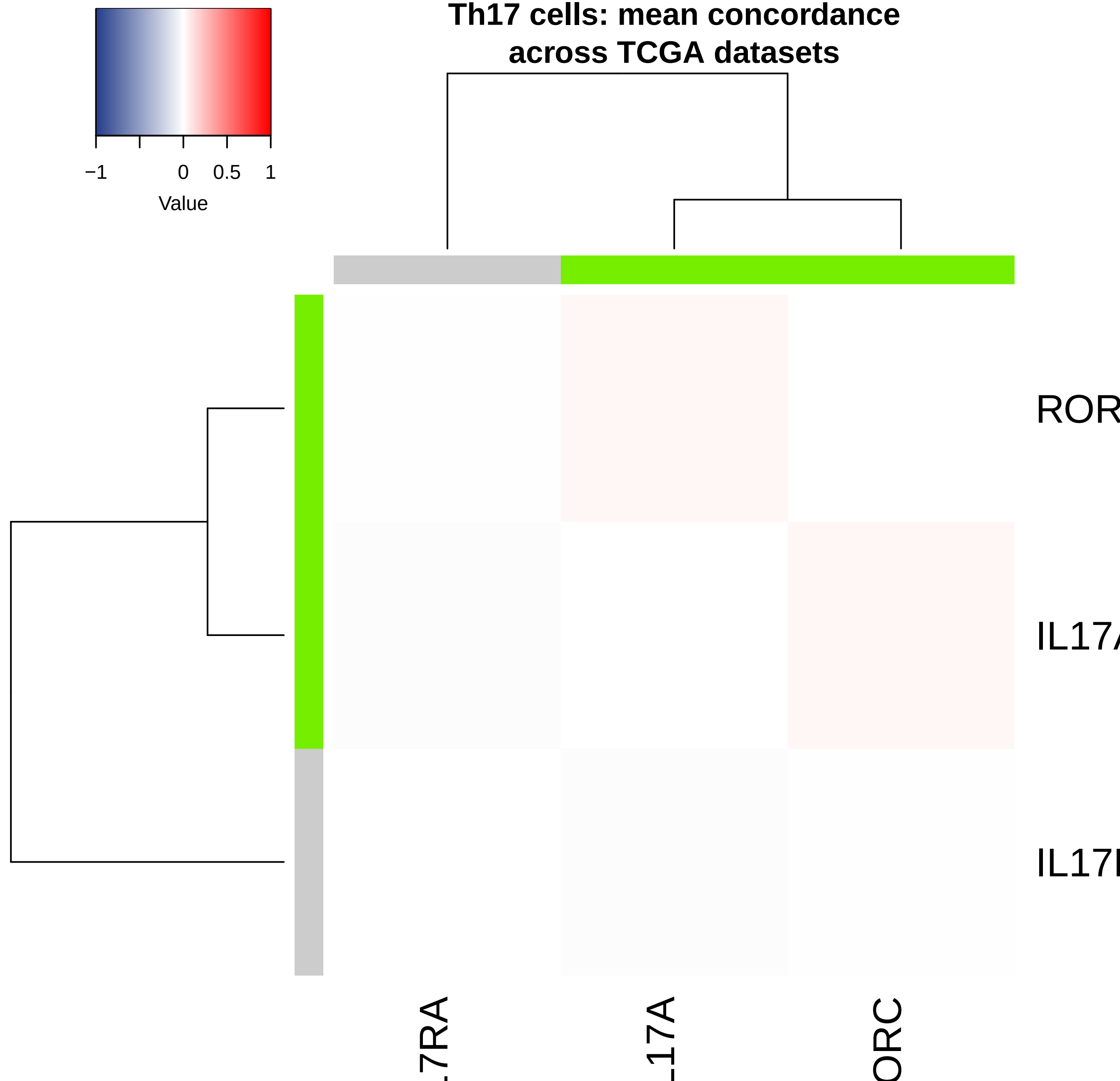

Figure S37:
Lymph vessels: mean concordance across TCGA datasets

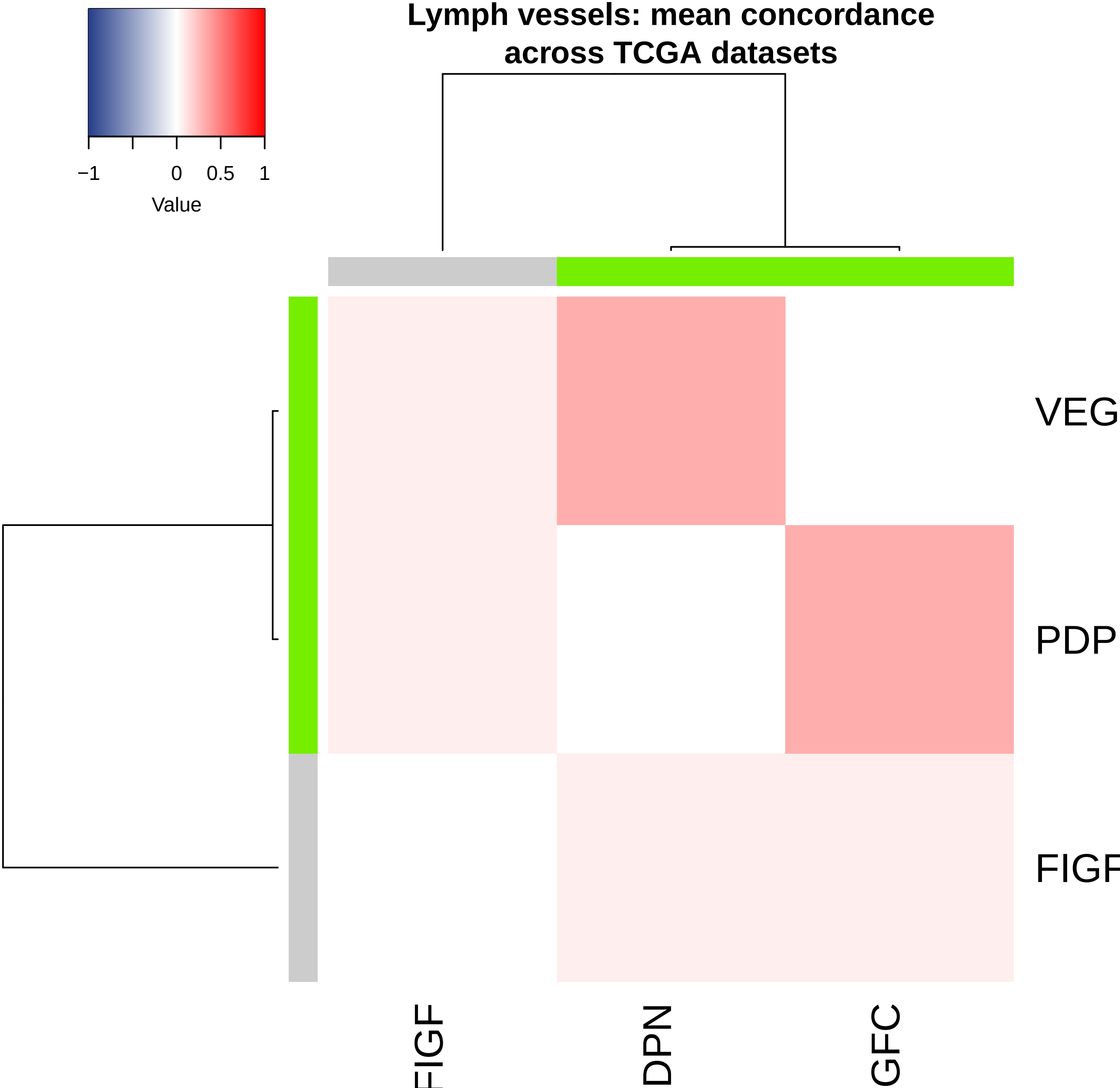

Figure S38:
Plasma cells: mean concordance across TCGA datasets

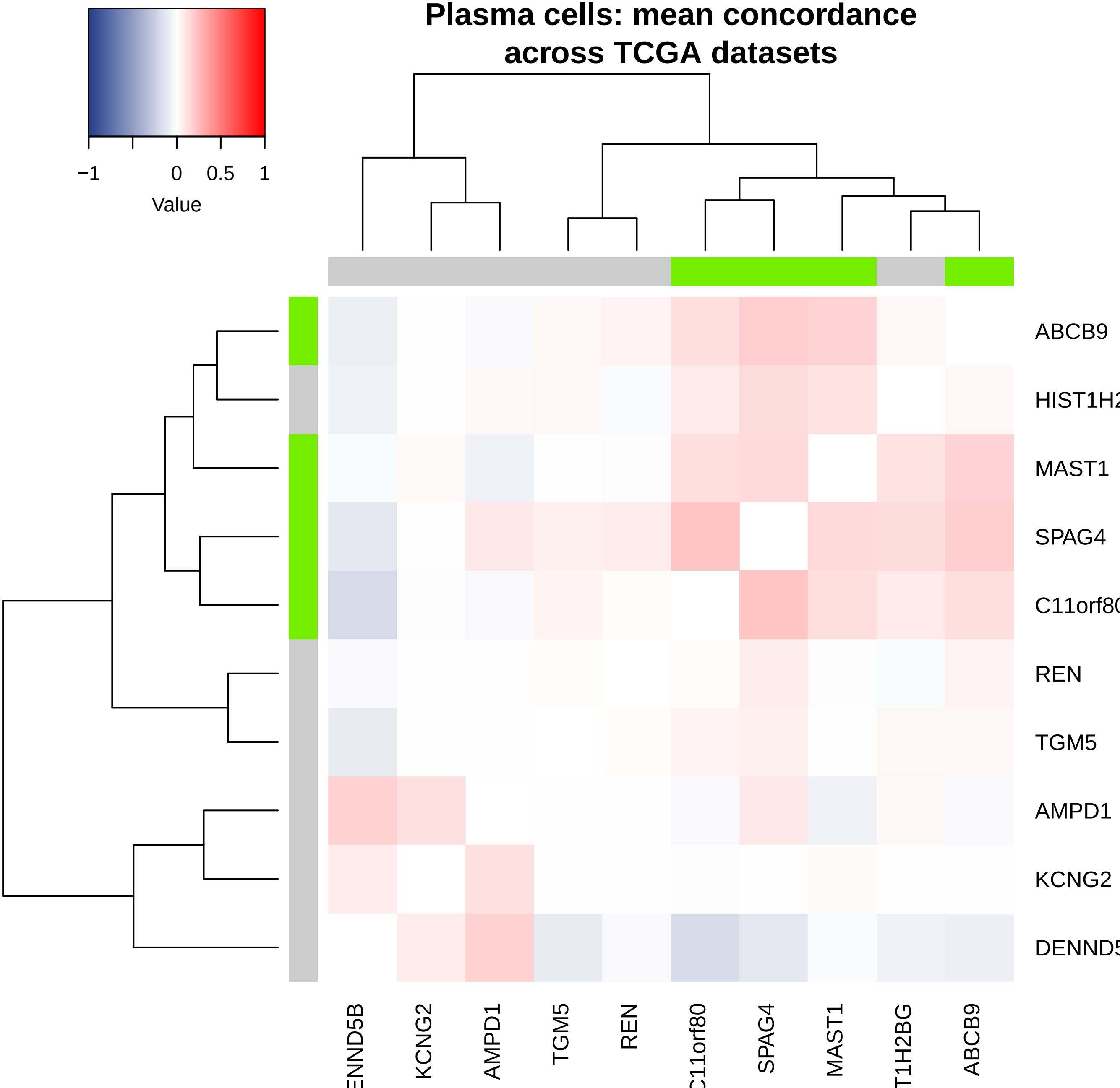

Supplement: Supplementary file 3 — Figure S10. Tem: mean concordance of cadidate cell type markers across TCGA datasets. Figure S11. T helper cells: mean concordance. Figure S12. Macrophages: mean concordance. Figure S13. MDSC: mean concordance. Figure S14. Tcm: mean concordance. Figure S15. NK cells: mean concordance. Figure S16. Th2 cells: mean concordance. Figure S17. B − cells: mean concordance. Figure S18. Neutrophils: mean concordance. Figure S19. Th1 cells: mean concordance. Figure S20. Normal mucosa: mean concordance. Figure S21. iDC: mean concordance. Figure S22. aDC: mean concordance. Figure S23. DC: mean concordance. Figure S24. Eosinophils: mean concordance. Figure S25. Tgd: mean concordance. Figure S26. T − cells: mean concordance. Figure S27. Exhausted CD8: mean concordance. Figure S28. CD8 T cells: mean concordance Figure S29. Mast cells: mean concordance. Figure S30. Treg: mean concordance. Figure S31. Cytotoxic cells: mean concordance. Figure S32. TFH: mean concordance. Figure S33. NK CD56bright cells: mean concordance. Figure S34. SW480 cancer cells: mean concordance. Figure S35. NK CD56dim cells: mean concordance. Figure S36. Th17 cells: mean concordance. Figure S37. Lymph vessels: mean concordance. Figure S38. Plasma cells: mean concordance. (PDF 949 kb) [file 40425_2017_215_MOESM3_ESM.pdf]
