# Supplementary material for: Gene expression markers of Tumor Infiltrating Leukocytes
Source: J Immunother Cancer. 2017 Feb 21;5:18. doi: 10.1186/s40425-017-0215-8 (PMC5319024; doi:10.1186/s40425-017-0215-8)
Supplement: Supplementary file 5 — All code and data. (ZIP 493984 kb) [file 40425_2017_215_MOESM5_ESM.zip › add5/Chen 2016 analysis.zip!/Chen 2016 analysis/chen results.pdf]

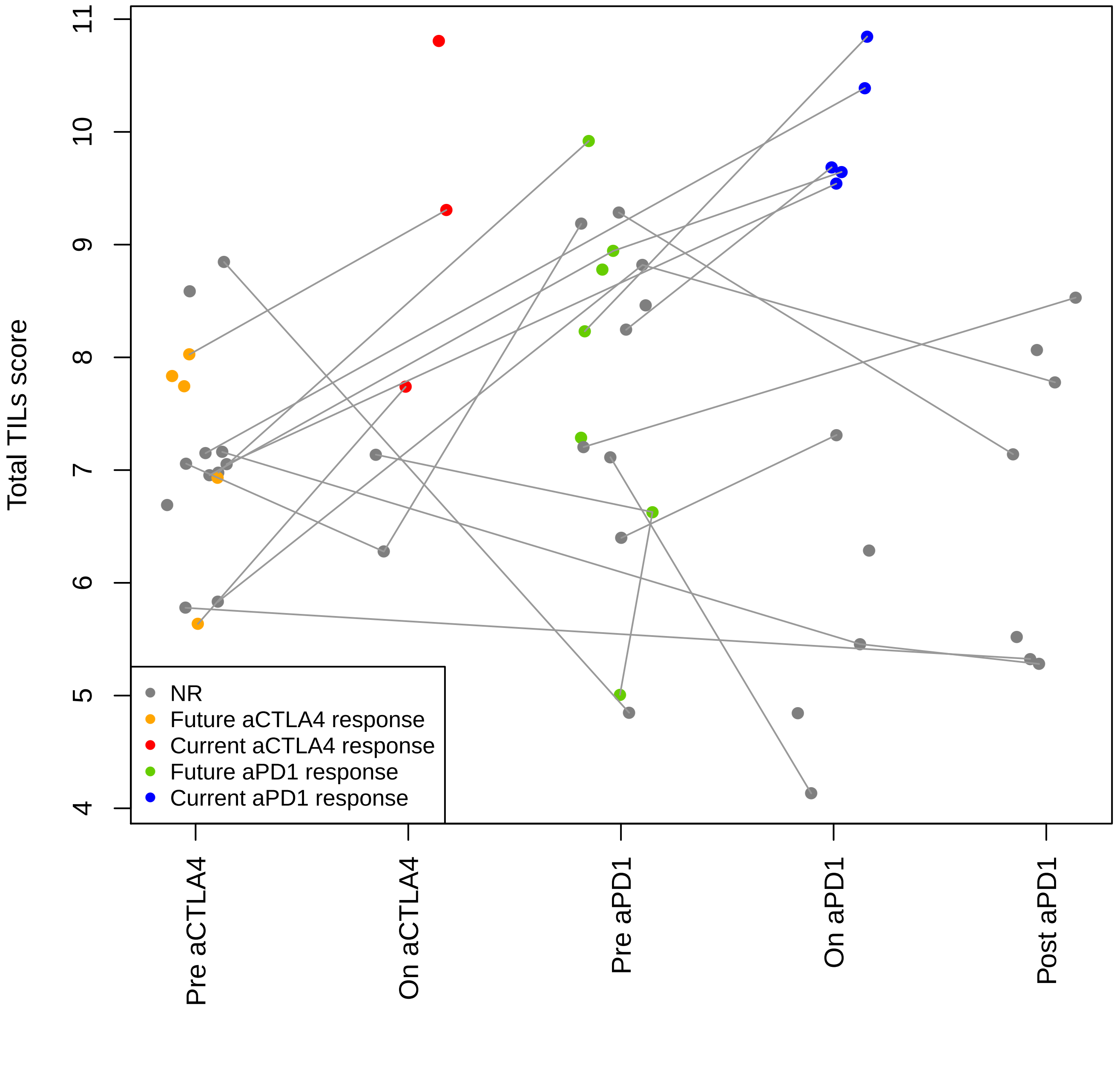
Total TILs score
11
10
9
8
7
6
5
4
Pre aCTLA4
On aCTLA4
Pre aPD1
On aPD1
Post aPD1
NR
Future aCTLA4 response
Current aCTLA4 response
Future aPD1 response
Current aPD1 response

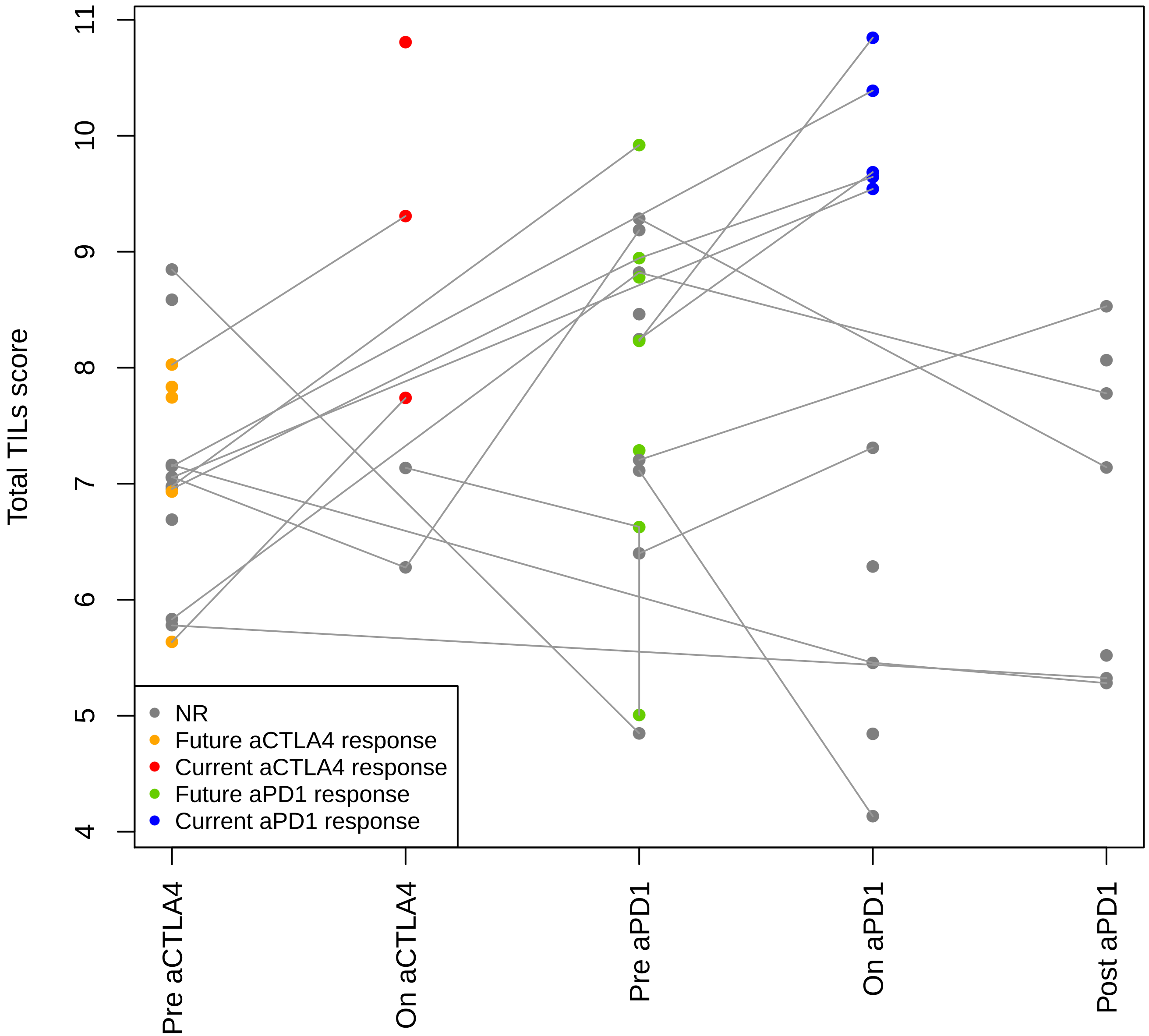

Total TILs score
11
10
9
8
7
6
5
4
NR
Future aCTLA4 response
Current aCTLA4 response
Future aPD1 response
Current aPD1 response
Pre aCTLA4
On aCTLA4
Pre aPD1
On aPD1
Post aPD1

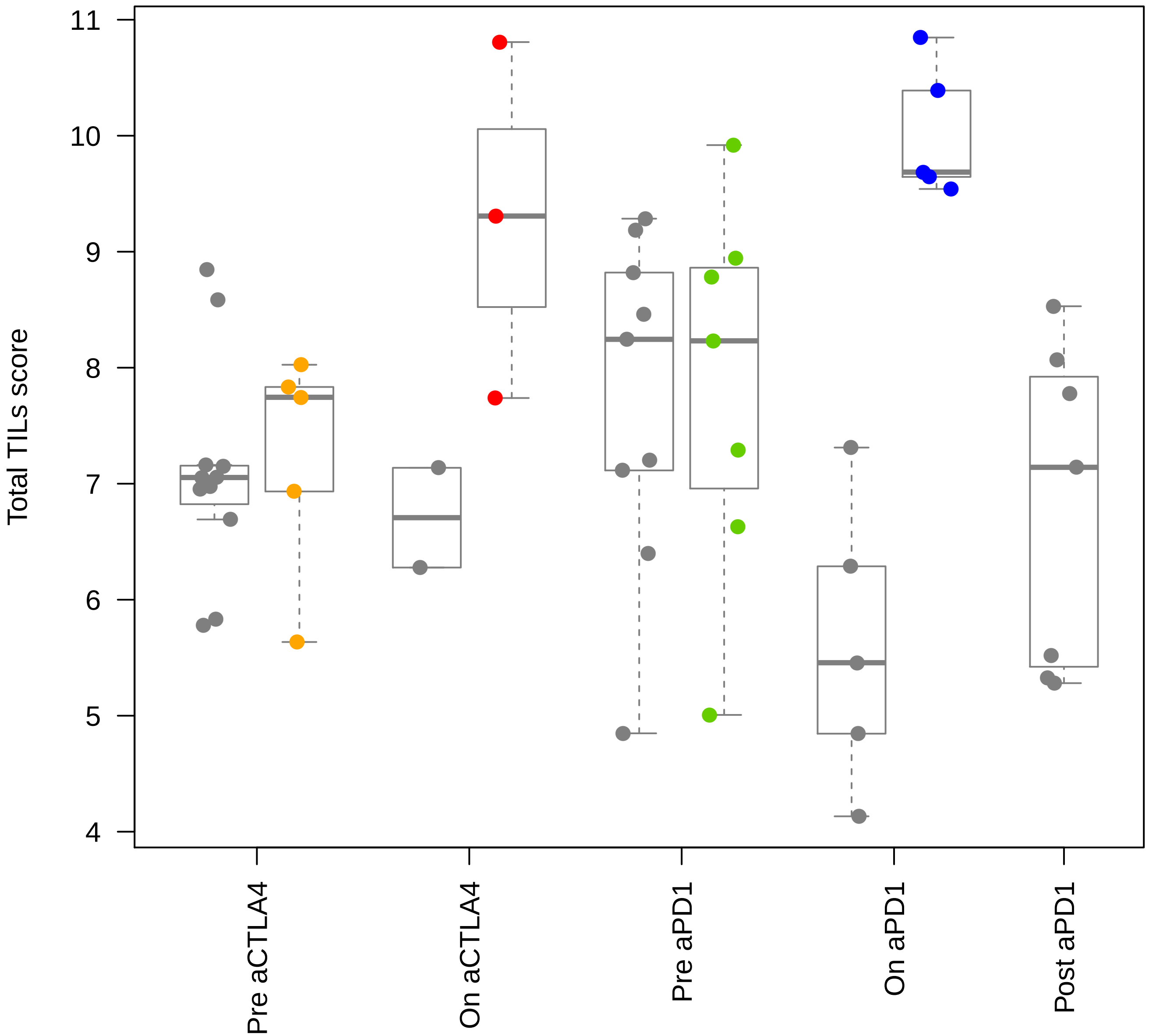

Total TILs score
11
10
9
8
7
6
5
4
Pre aCTLA4
On aCTLA4
Pre aPD1
On aPD1
Post aPD1

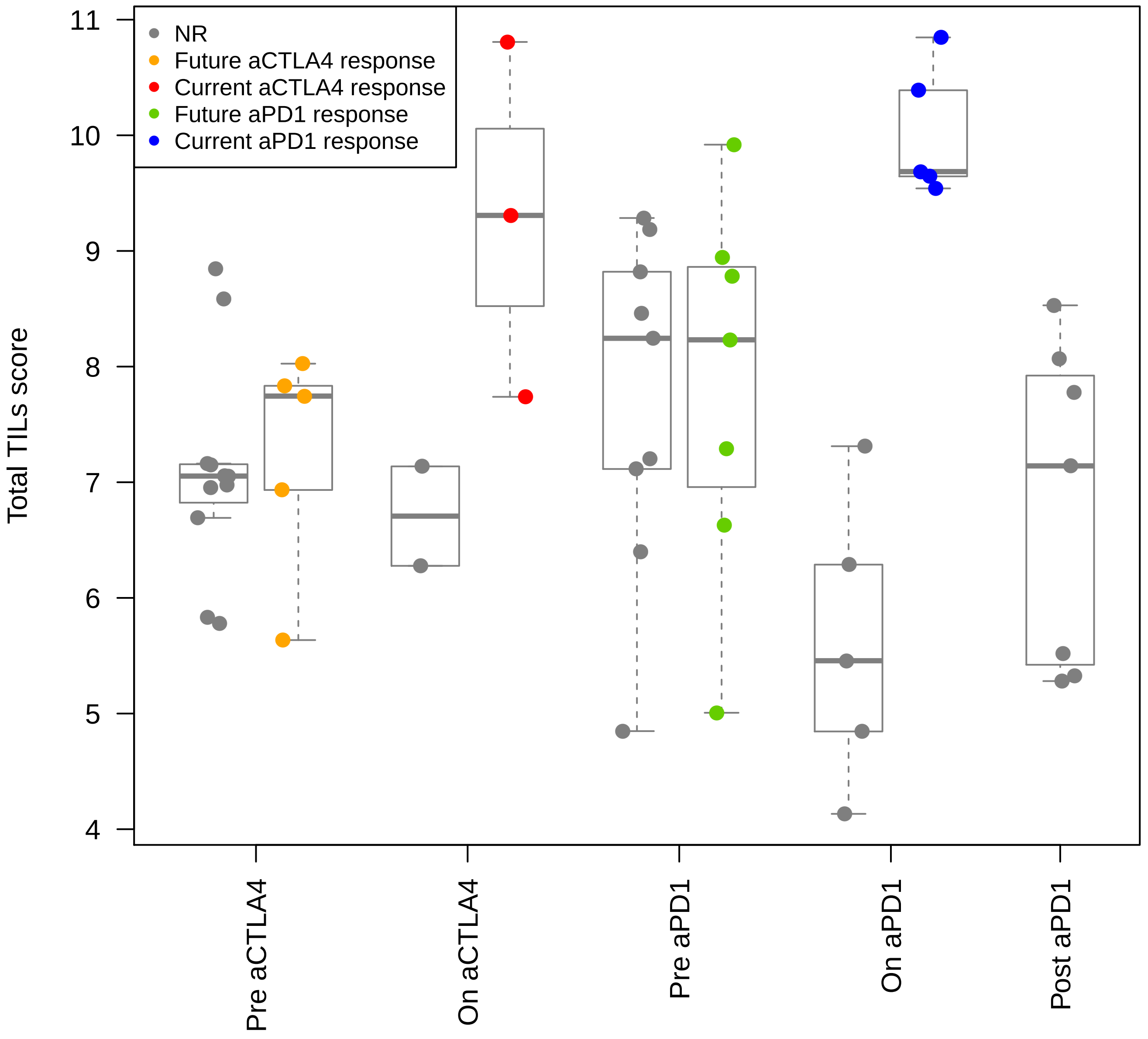

NR
Future aCTLA4 response
Current aCTLA4 response
Future aPD1 response
Current aPD1 response
Total TILs score
11
10
9
8
7
6
5
4
Pre aCTLA4
On aCTLA4
Pre aPD1
On aPD1
Post aPD1

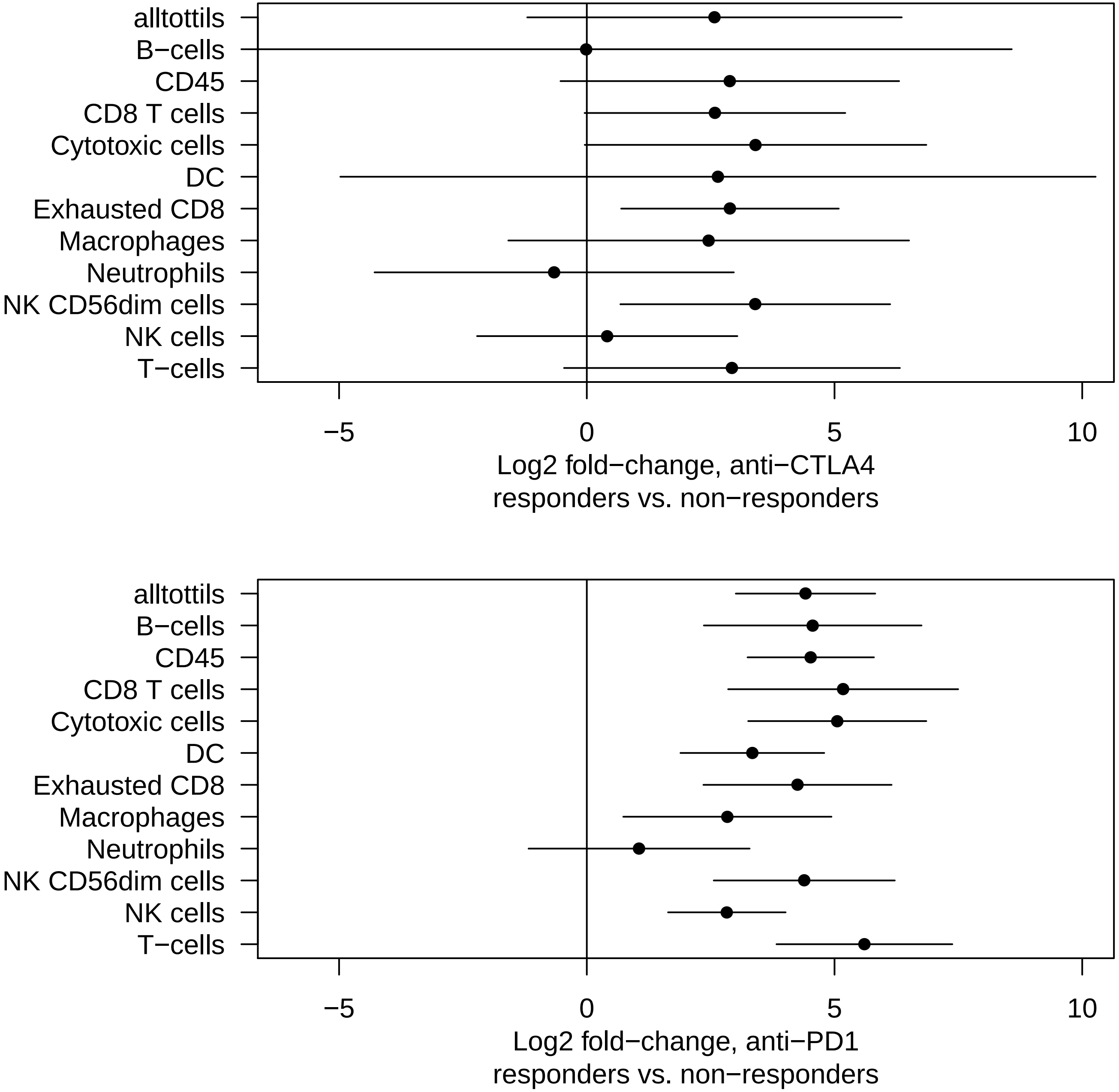
alltottils
B−cells
CD45
CD8 T cells
Cytotoxic cells
DC
Exhausted CD8
Macrophages
Neutrophils
NK CD56dim cells
NK cells
T−cells
−5
0
5
10
Log2 fold−change, anti−CTLA4
responders vs. non−responders
alltottils
B−cells
CD45
CD8 T cells
Cytotoxic cells
DC
Exhausted CD8
Macrophages
Neutrophils
NK CD56dim cells
NK cells
T−cells
−5
0
5
10
Log2 fold−change, anti−PD1
responders vs. non−responders

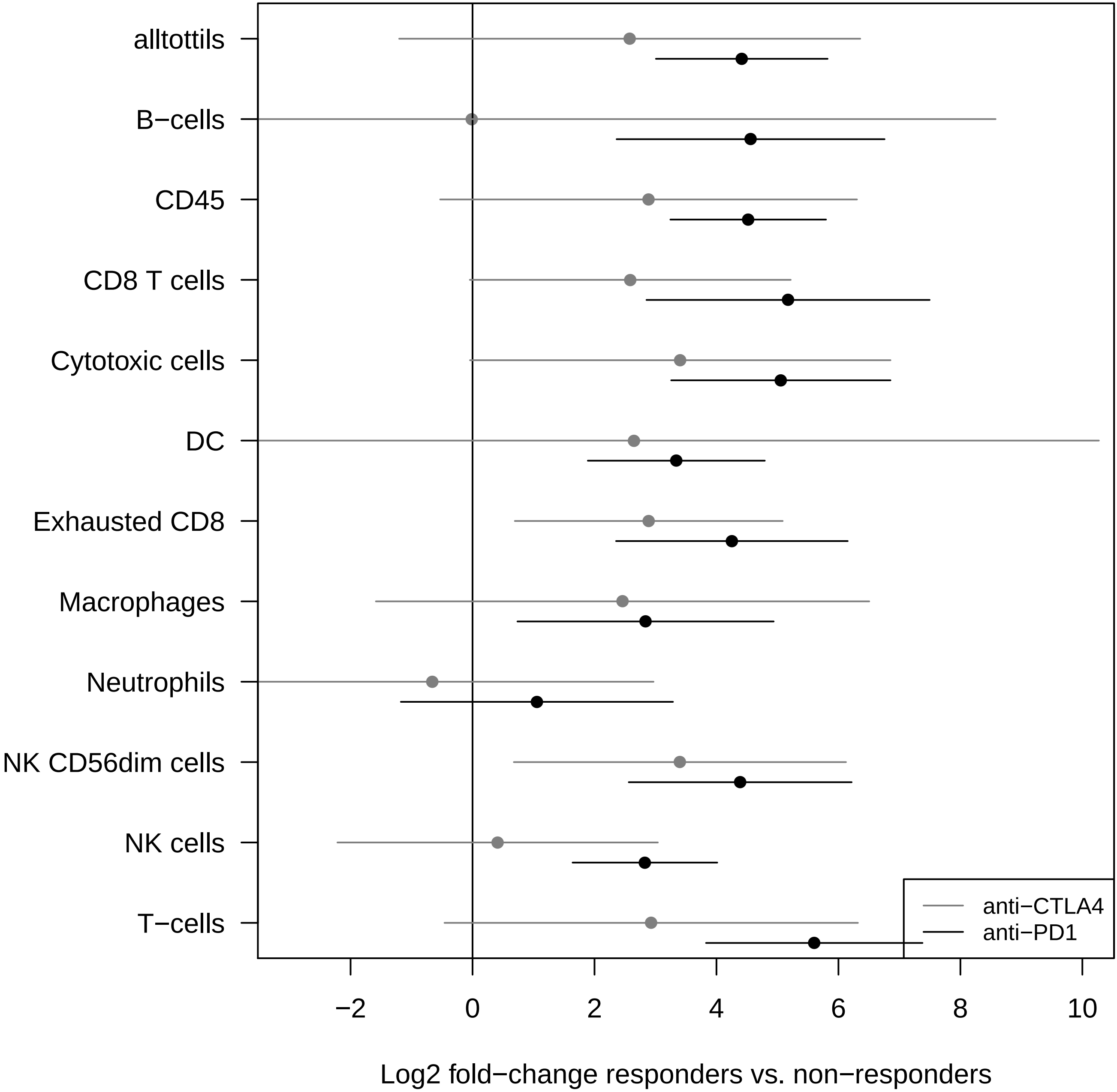
alltottils
B−cells
CD45
CD8 T cells
Cytotoxic cells
DC
Exhausted CD8
Macrophages
Neutrophils
NK CD56dim cells
NK cells
T−cells
anti−CTLA4
anti−PD1
−2
0
2
4
6
8
10
Log2 fold−change responders vs. non−responders

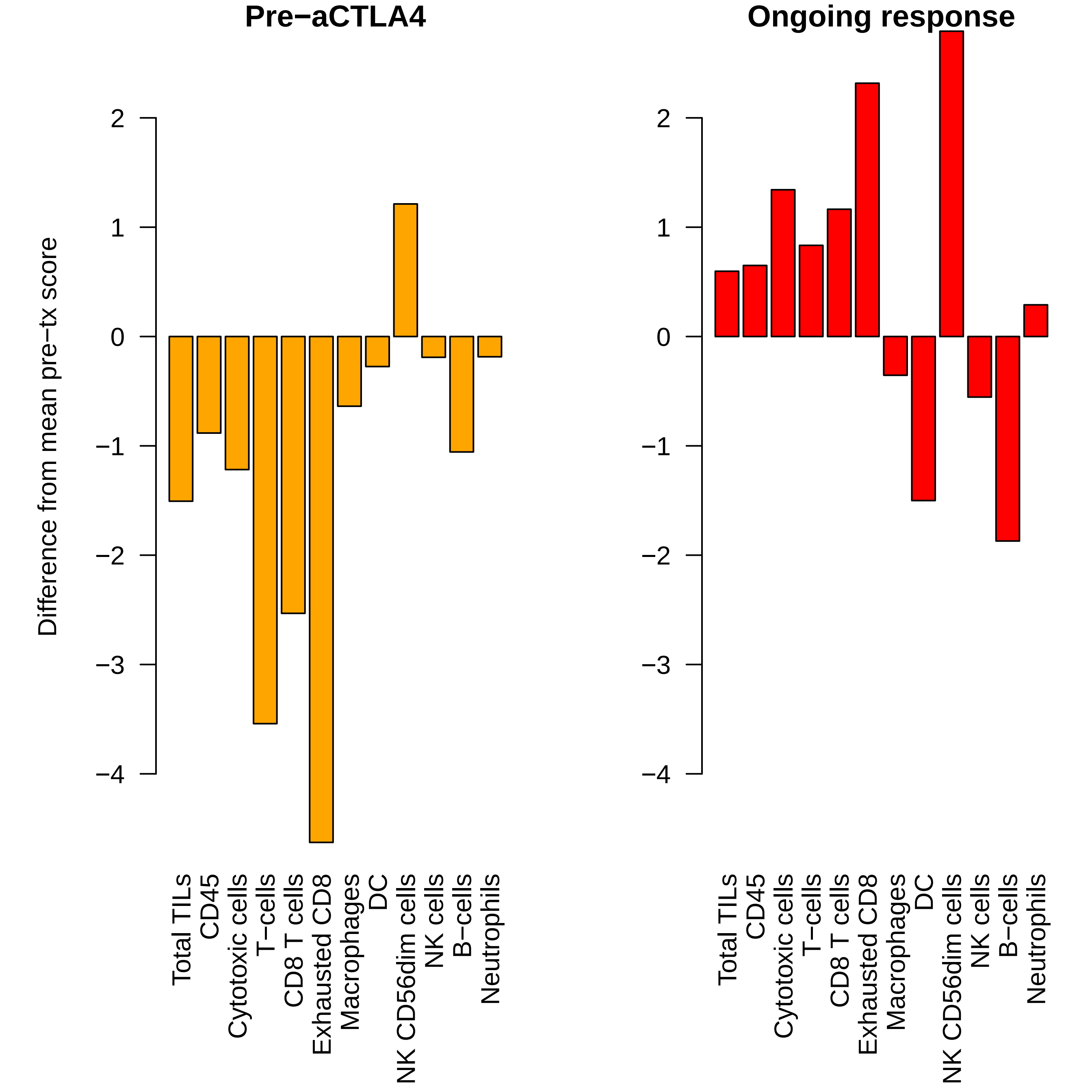
Pre−aCTLA4
Ongoing response
Difference from mean pre−tx score
2
1
0
−1
−2
−3
−4
Total TILs
CD45
Cytotoxic cells
T−cells
CD8 T cells
Exhausted CD8
Macrophages
DC
NK CD56dim cells
NK cells
B−cells
Neutrophils

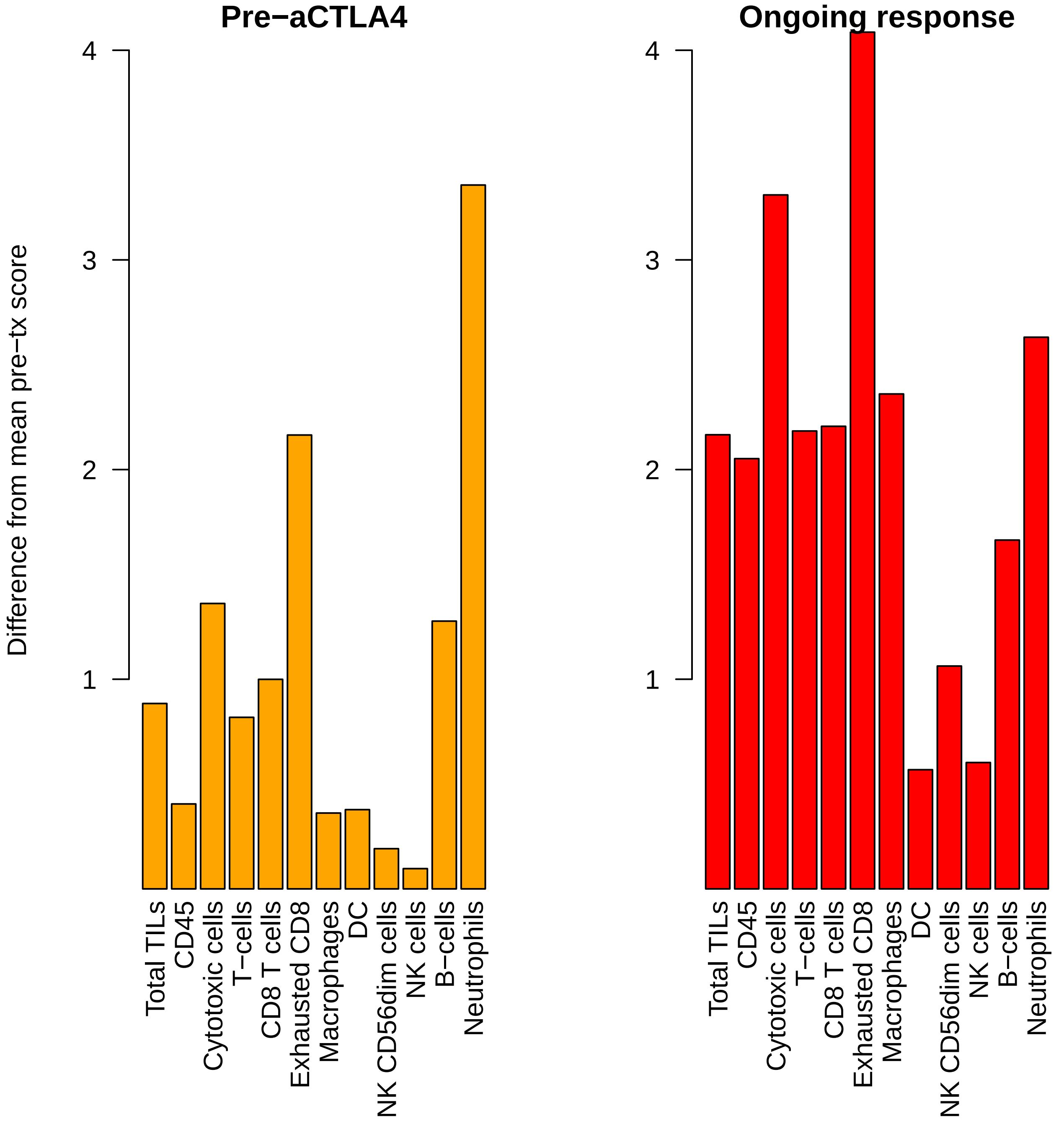

Pre−aCTLA4
Ongoing response
Difference from mean pre−tx score
4
3
2
1
Total TILs
CD45
Cytotoxic cells
T−cells
CD8 T cells
Exhausted CD8
Macrophages
DC
NK CD56dim cells
NK cells
B−cells
Neutrophils
